# Stress-induced epinephrine enhances lactate dehydrogenase A and promotes breast cancer stem-like cells

**DOI:** 10.1172/JCI121685

**Published:** 2019-01-28

**Authors:** Bai Cui, Yuanyuan Luo, Pengfei Tian, Fei Peng, Jinxin Lu, Yongliang Yang, Qitong Su, Bing Liu, Jiachuan Yu, Xi Luo, Liu Yin, Wei Cheng, Fan An, Bin He, Dapeng Liang, Sijin Wu, Peng Chu, Luyao Song, Xinyu Liu, Huandong Luo, Jie Xu, Yujia Pan, Yang Wang, Dangsheng Li, Peng Huang, Qingkai Yang, Lingqiang Zhang, Binhua P. Zhou, Suling Liu, Guowang Xu, Eric W.-F. Lam, Keith W. Kelley, Quentin Liu

**Affiliations:** 1 Institute of Cancer Stem Cell, Dalian Medical University, Dalian, China.; 2 State Key Laboratory of Oncology in South China, Cancer Center, Sun Yat-sen University, Guangzhou, China.; 3 CAS Key Laboratory of Separation Science for Analytical Chemistry, Dalian Institute of Chemical Physics, Chinese Academy of Sciences, Dalian, China.; 4 Center for Molecular Medicine, School of Life Science and Biotechnology, Dalian University of Technology, Dalian, China.; 5 Department of Oncology, The First Affiliated Hospital of Dalian Medical University, Dalian, China.; 6 Shanghai Information Center for Life Sciences, Shanghai Institutes for Biological Sciences, Chinese Academy of Sciences, Shanghai, China.; 7 State Key Laboratory of Proteomics, National Center of Protein Sciences, Beijing Institute of Lifeomics, Beijing, China.; 8 Department of Molecular and Cellular Biochemistry, Markey Cancer Center, University of Kentucky, College of Medicine, Lexington, Kentucky, USA.; 9 Fudan University Shanghai Cancer Center and Institutes of Biomedical Sciences, Shanghai, China.; 10 Department of Surgery and Cancer, Imperial College London, London, United Kingdom.; 11 Laboratory of Immunophysiology, Department of Animal Sciences, College of Agricultural, Consumer and Environmental Sciences, and Department of Pathology, College of Medicine, University of Illinois at Urbana-Champaign, Urbana, Illinois, USA.

**Keywords:** Metabolism, Oncology, Breast cancer

## Abstract

Chronic stress triggers activation of the sympathetic nervous system and drives malignancy. Using an immunodeficient murine system, we showed that chronic stress–induced epinephrine promoted breast cancer stem-like properties via lactate dehydrogenase A–dependent (LDHA-dependent) metabolic rewiring. Chronic stress–induced epinephrine activated LDHA to generate lactate, and the adjusted pH directed USP28-mediated deubiquitination and stabilization of MYC. The *SLUG* promoter was then activated by MYC, which promoted development of breast cancer stem-like traits. Using a drug screen that targeted LDHA, we found that a chronic stress–induced cancer stem-like phenotype could be reversed by vitamin C. These findings demonstrated the critical importance of psychological factors in promoting stem-like properties in breast cancer cells. Thus, the LDHA-lowering agent vitamin C can be a potential approach for combating stress-associated breast cancer.

## Graphical abstract

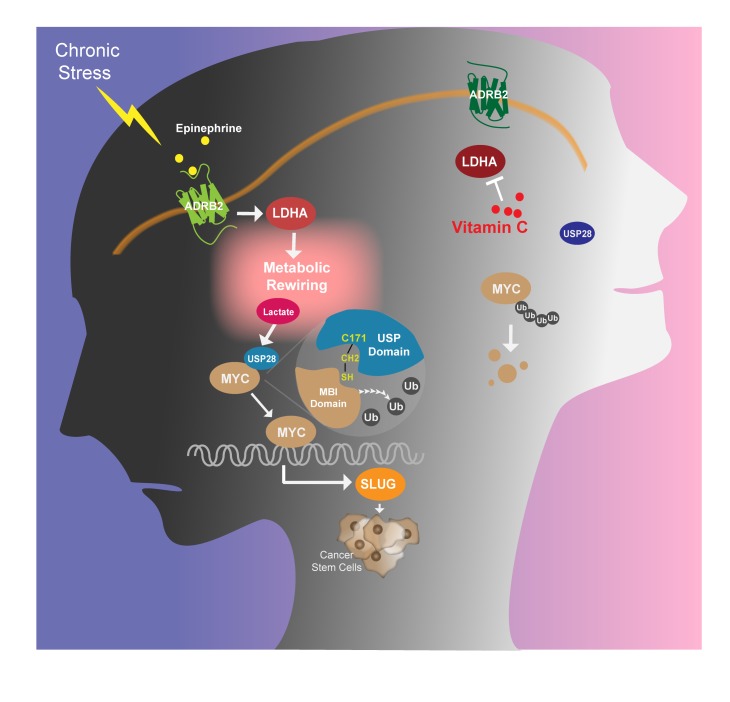

## Introduction

Patients suffering from cancer often experience a variety of chronic emotional stressors (1), including depression, anxiety, and fear (2, 3). These serve as risk factors by facilitating tumor growth and increasing expression of invasion-related genes that promote cancer progression (4). Indeed, chronic stress increases catecholamine levels and promotes tumor burden and invasive growth of ovarian carcinoma cells in vivo (5). Moreover, stress-induced hormones have been shown to increase cancer cell dissemination in pancreatic cancer (4). Immune activity has long been established as being suppressed by chronic stress and is considered to be responsible for promoting cancer (6, 7). Yet, the direct signaling network between stress pathways and a cancer-propagating program remains almost completely unknown.

Cancer stem-like cells (CSCs) are characterized by an increased capability of self-renewal (8) and tumor reconstitution (9). They are able to generate heterogeneous lineages of cancer cells that constitute tumors. These CSCs are important for the initiation, maintenance, and clinical outcome of many cancers. Previous studies have demonstrated that transcription factors such as MYC, SLUG, and SOX2 are responsible for tumorigenesis and can reprogram cells from a differentiated to a stem-like state in a variety of cancers (10–12). Indeed, the transcription factor MYC plays key roles in oncogenesis and is involved in many cancer networks (13). MYC increases SOX2 transcriptional activity, forming a positive-feedback loop involving the Wnt/β-catenin/MYC/SOX2 axis, which defines a highly tumorigenic cell subpopulation in ALK-positive anaplastic large cell lymphomas (14). Moreover, SOX2 represses microRNA-452 (miR-452), which acts as a metastasis suppressor to directly target the *SLUG* 3′-untranslated region (3′-UTR). Taken together with miR-452 loss and SLUG upregulation, SOX2 provides a potentially novel mechanism by which CSCs acquire metastatic potential (15).

Lactate dehydrogenase A (LDHA) executes the final step of the Warburg effect by converting pyruvate to lactate. Moreover, LDHA-associated lactic acid production leads to a relatively low pH, allowing cancer cells to survive immune evasion via diminishing nuclear factor of activated T cells (NFAT) levels and T and NK cell activation (16, 17). Deregulation of LDHA has been reported in a number of malignancies, including prostate, breast, hepatocellular, and gastrointestinal cancers (18–20). Inhibition of LDHA reduces malignant transformation and delays tumor formation, indicating an important role for LDHA in tumor initiation and progression (21). As might be predicted, LDHA consistently elevates “stemness” properties of CSCs and enhances spheroid formation in hepatocellular cancer (22). In this work, we define what to our knowledge is a novel molecular pathway by which chronic stress acts via β_2_-adrenergic receptor to elevate LDHA. This leads to a switch to lactate production, and the adjusted pH then directs USP28-mediated deubiquitination and stabilization of MYC, thereby promoting stem-like traits in breast cancer. These data provide what to our knowledge is a novel pathway that explains how chronic stress promotes breast cancer progression by acting directly on CSCs.

## Results

### Chronic stress promotes breast cancer stem-like traits via epinephrine-ADRB2.

**Figure 1:**
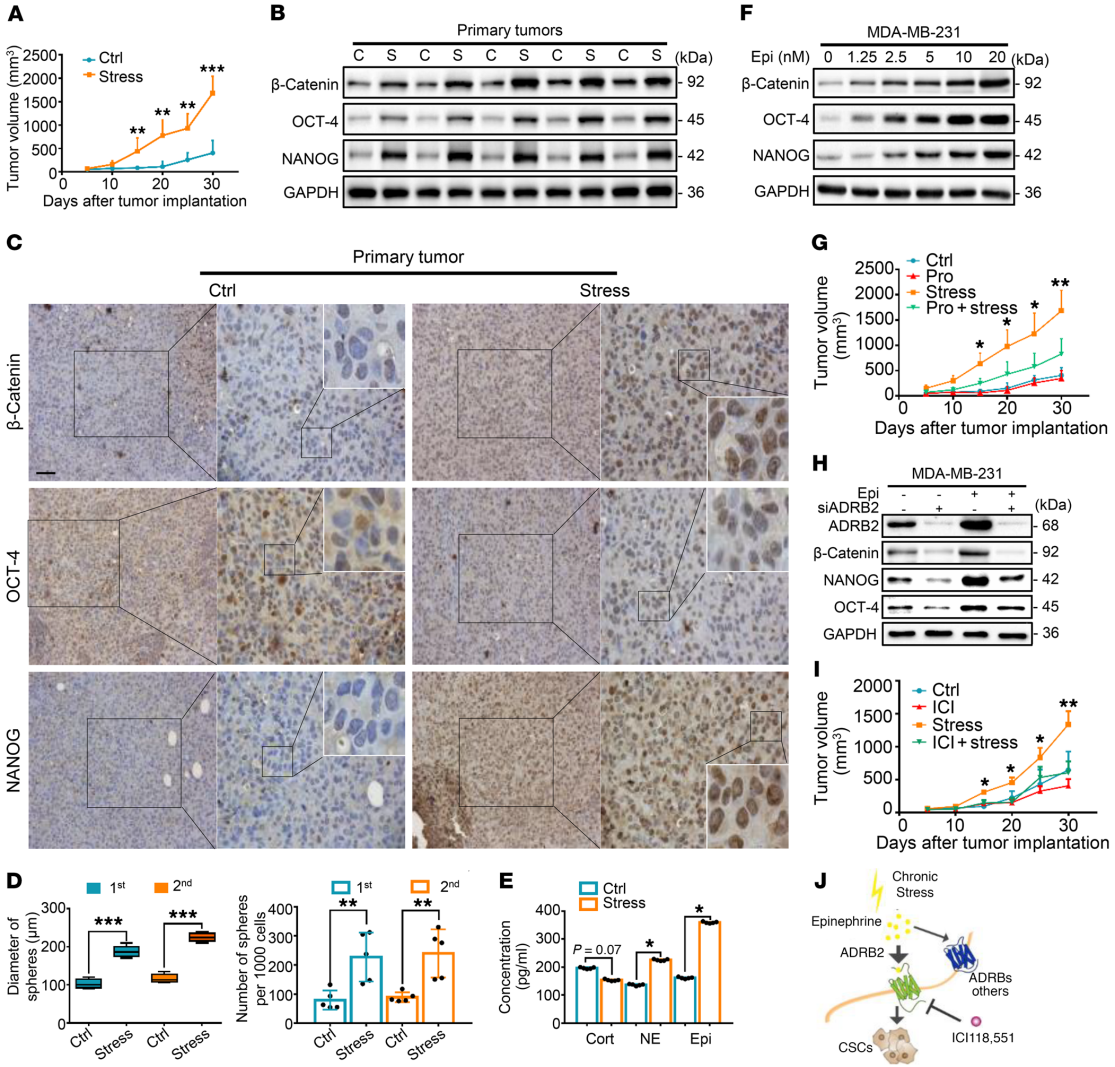
Chronic stress promotes ADRB2-dependent cancer stem cell–like properties in vivo. (**A**) Tumor growth of MDA-MB-231 tumors in control (Ctrl) and stressed mice; *n* = 5 (1-way ANOVA). (**B**–**D**) Primary MDA-MB-231 tumors from the Ctrl and stress groups were subjected to immunoblot (C, control; S, stressed) (**B**), immunohistochemical staining (scale bar: 50 μm; original magnification, ×20, ×40, ×96 [insets]) (**C**), and primary and secondary spheroid formation; *n* = 5 (1-way ANOVA) (**D**). (**E**) Concentrations (pg/ml) of cortisol (Cort), norepinephrine (NE), and epinephrine (Epi) in serum of Ctrl and stress mice after the last day of stress; *n* = 5 (Student’s *t* test). (**F**) Immunoblot analysis of indicated antibodies in MDA-MB-231 cells treated with indicated concentrations of Epi. (**G**) Growth of Ctrl, propranolol (Pro), stress, and stress-induced propranolol-treated (Pro + stress) MDA-MB-231 tumors in mice; *n* = 6 (1-way ANOVA). (**H**) MDA-MB-231 cells were transfected with siADRB2 and then treated with Epi for 5 days. Expression of proteins was determined by immunoblot analysis. (**I**) Growth of MDA-MB-231 tumors in Ctrl and stress mice in the presence or absence of ICI118,551 (ICI); *n* = 5 (1-way ANOVA). (**J**) Model of chronic stress–mediated cancer stem-like traits mediated by β_2_-adrenergic receptor (ADRB2) signaling. Data are representative of at least 3 independent experiments. Data represent mean ± SEM; *P < 0.05, **P < 0.01, ***P < 0.001.

As described previously (5), we adapted an accepted chronic stress model to nonobese diabetic–severe combined immunodeficient (NOD/SCID) mice and examined the effects of stress on both tumor growth and CSC self-renewal ability (Supplemental Figure 1A; supplemental material available online with this article; https://doi.org/10.1172/JCI121685DS1). Beginning from 15 days after cancer cell implantation, tumors from stressed mice were larger than those from control mice (Figure 1A and Supplemental Figure 1B). Even though there was no difference in body weight between the control and stressed groups (Supplemental Figure 1C), tumors from the chronic stress group continued to increase throughout the entire 30-day stress paradigm. Subsequently, mice were subjected to behavioral assays using both the tail suspension test and the open field test. Chronically stressed mice exhibited more anxiogenic and depression-like behaviors than control mice (Supplemental Figure 1, D and E). Consistently, C57BL/6 mice, the immunocompetent mice, were injected with E0771 and Py8119 cells under stress. The results indicated that stress enhanced the tumor burden in the C57BL/6 mouse model (Supplemental Figure 1F).

After euthanasia in order to collect the xenografted tumors, we found that stress-induced tumors expressed significantly higher levels of self-renewal genes. These included *CTNNB*, *POU5F1*, and *NANOG*, as measured by both mRNA (Supplemental Figure 1G) and protein expression (Figure 1B and Supplemental Figure 1H). Similar results were also observed by immunohistochemical analyses (Figure 1C and Supplemental Figure 1I). Next, single-cell suspensions were prepared by enzymatic digestion from xenografts of both control and stressed mice. Mammosphere transplantation assays were used to assess their self-renewal abilities in vitro. Tumor cells from the stressed group displayed greater mammosphere-forming efficiencies in both the primary and secondary generations, as indicated by a significant increase in both spheroid diameter and number (Figure 1D and Supplemental Figure 1J). Stress significantly increased sphere formation frequencies of primary tumor cells as examined by in vitro limiting dilution assays (Supplemental Figure 1K). In vivo, serially diluted primary tumor cells were subcutaneously inoculated at 4 different sites into each group of secondary mice (Supplemental Figure 1L). Notably, tumor formation rates of stressed mice were increased. At the lowest number of implanted tumor cells (10^2^), primary stressed tumor cells increased tumor formation efficiency from 20% to 70% in secondary control mice, and enhanced tumor formation efficiency to 100% in secondary stressed mice (Table 1). As expected, tumor formation in control and stressed mice did not differ at the highest dose of tumor cells (10^5^; 80% in control vs. 100% in stressed mice).

Immediately after the last day of stress, we collected blood from all mice. Subsequently, we examined the serum concentration of the major adrenal stress hormones, including cortisol, norepinephrine, and epinephrine (23). Serum levels of epinephrine displayed a sharp increase in the stress-induced group compared with those with control treatment, whereas serum levels of cortisol did not differ significantly between groups (Figure 1E). We then monitored xenograft growth in NOD/SCID mice injected with epinephrine. We found that tumors from epinephrine-treated mice were larger than those from PBS-treated mice, an effect that occurred as soon as 15 days following tumor implantation (Supplemental Figure 2A). Both diameter and number of mammospheres derived from the epinephrine-injected group were significantly increased compared with those from PBS-treated control mice (Supplemental Figure 2B). Consistently, epinephrine increased the expression of self-renewal factors in a dose-dependent manner (Figure 1F). Norepinephrine marginally enhanced but cortisol had no effect on the expression of self-renewal factors (Supplemental Figure 2, C and D).

Following injection of propranolol, an antagonist of adrenergic β-receptors (ADRBs), stressed mice demonstrated a significant decrease in tumor volume when compared with the stress-only control group (Figure 1G). Notably, treatment with propranolol caused a substantial reduction in tumor formation efficiency and blocked stress-induced tumorigenesis (Tables 2 and 3). The potential role of ADRB1 or ADRB2 was then determined by silencing of ADRB1 and ADRB2 with small interfering RNA (siRNA). This approach significantly blunted the stem-like cell phenotype (Supplemental Figure 2, E and F). ADRB2 depletion efficiently blocked the epinephrine-induced enhancement of breast CSCs (BCSCs) (Figure 1H and Supplemental Figure 2G), whereas ADRB1 knockdown did not (Supplemental Figure 2H). Similarly, the ADRB2 inhibitor ICI118,551 blocked the epinephrine-increased β-catenin, OCT-4, and NANOG expression, whereas the ADRB1 inhibitor atenolol only had a marginal effect (Supplemental Figure 2I). Further investigation demonstrated a similar reduction in tumor burden with the ADRB2 antagonist ICI118,551 (Figure 1I and Supplemental Figure 2J).

Following stress treatment for 5 weeks in the metastatic mouse model, we found that stressed mice displayed more lung metastatic lesions as assessed by CT scans. Nodules on the surface of lungs were counted, which revealed more nodules in the lungs of stressed compared with control mice (Supplemental Figure 2K). In contrast, shMYC- and shSLUG-mediated depletion reversed the ability of stress to enhance lung metastasis. These data indicate that chronic stress promotes the potential metastasis of cancer via MYC and SLUG. Furthermore, epinephrine-treated cells showed increased migration, invasion, and wound-healing abilities (Supplemental Figure 2, L and M). We also performed immunohistochemistry to examine changes in SNAIL1 and TWIST expression in stressed tumor tissues. The results indicated enhanced expression of SNAIL1 and TWIST in stressed tumors compared with control tumors (Supplemental Figure 2N). Collectively, these data demonstrate that chronic stress–induced epinephrine promotes breast cancer stem-like properties by acting through ADRB2 (Figure 1J).

### Chronic stress–induced MYC activates SLUG transcription to stimulate CSCs.

**Figure 2:**
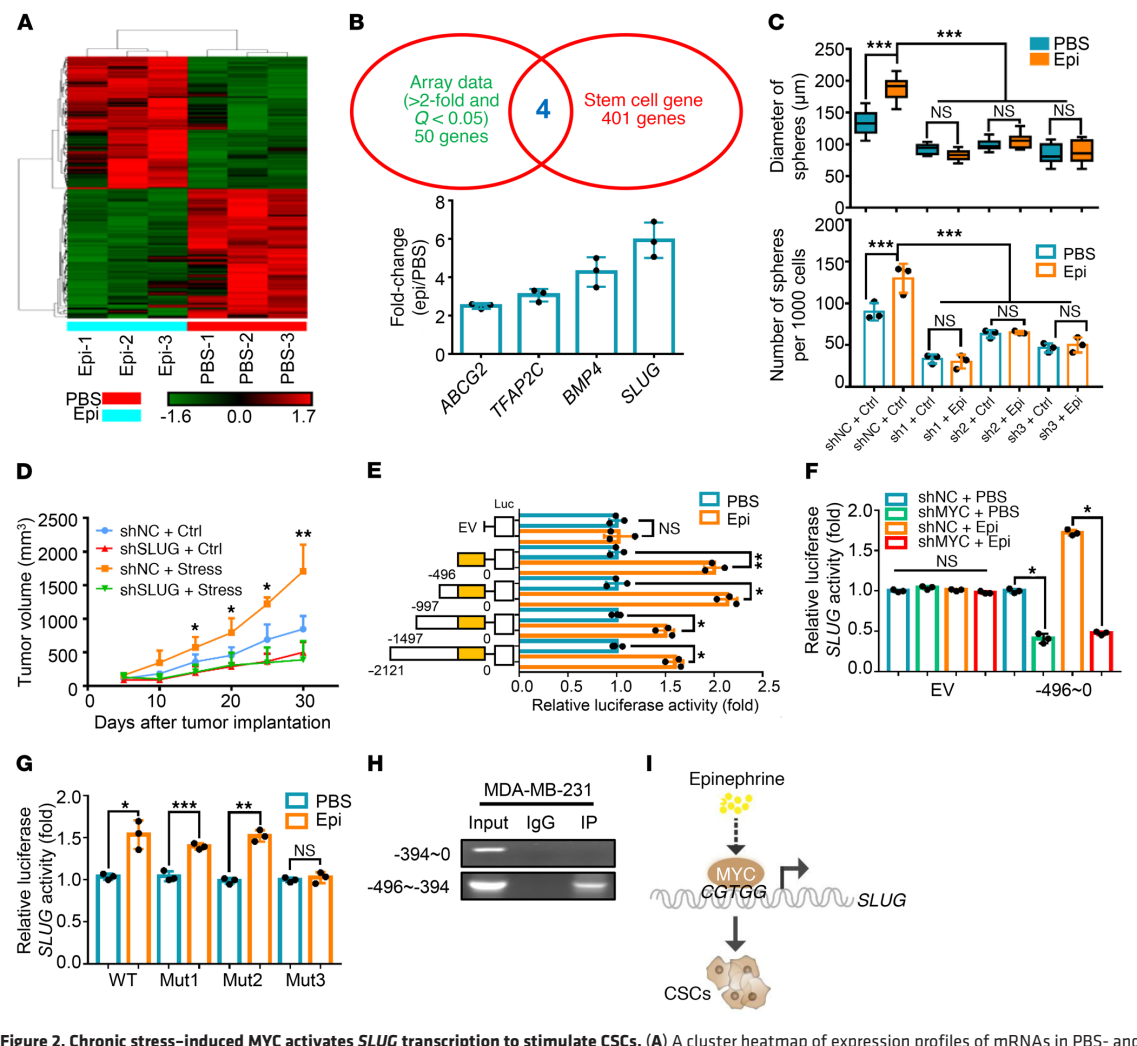
Chronic stress–induced MYC activates *SLUG* transcription to stimulate CSCs. (**A**) A cluster heatmap of expression profiles of mRNAs in PBS- and Epi-treated MDA-MB-231–derived tumors; *n* = 3. (**B**) Comparison of array data (fold change >2, *Q* < 0.05, 54 genes) with stem-like cell genes (405 genes). Common genes were verified by Epi treatment and are listed in the column according to fold change. *n* = 3. (**C**) Distribution patterns and number (d > 50 μm) of mammospheres from the negative control (shNC) or shSLUG MDA-MB-231 cells after treatment with PBS or Epi for 5 days; *n* = 3 (1-way ANOVA). (**D**) Growth of shNC or shSLUG-3 MDA-MB-231 tumors in mice with or without stress treatment; *n* = 6 (1-way ANOVA). (**E**) Dual-luciferase reporter assays of MDA-MB-231 cells transfected with *SLUG* truncated promoters or empty vector (EV) in the presence or absence of Epi for 5 days; *n* = 3 (1-way ANOVA). (**F**) Dual-luciferase analysis in shNC or shMYC MDA-MB-231 cells treated with PBS or Epi for 5 days and transfected with EV or *SLUG* promoter (–496 to 0); *n* = 3 (1-way ANOVA). (**G**) Dual-luciferase reporter assays of MDA-MB-231 cells treated with Epi for 5 days and transfected with *SLUG* WT, mutant 1 (Mut1, –57 to –54), mutant 2 (Mut2, –104 to –101), or mutant 3 (Mut3, –412 to –408) promoters; *n* = 3 (1-way ANOVA). (**H**) ChIP-PCR analysis in MDA-MB-231 cells of MYC occupancy on the *SLUG* promoter. (**I**) Model of Epi-induced cancer stem-like traits through MYC/SLUG signaling. Data are representative of at least 3 independent experiments. Data represent mean ± SEM; *P < 0.05, **P < 0.01, ***P < 0.001.

In order to investigate the downstream targets responsible for the increase in stress-mediated stem-like properties in breast cancer, we used Affymetrix Human PrimeView (analyzed from independent triplicates) to perform a large-scale expression profile analysis (Figure 2A). To further analyze the functional importance of the altered gene sets (Table 4), we performed Gene Ontology (GO) analysis including Biochemical Process, Cell Component, and Molecular Function, and found that some important metabolic processes and cell migration were among the top 10 of GO analysis (Supplemental Figure 3A). Moreover, comparing the significantly altered genes in microarray data with the Cancer Stem Cells Therapeutic Target Database (24) and stem-like cell gene sets (8), we found that 4 stemness-associated genes displayed significantly altered expression levels in epinephrine-induced tumors. Further verification of these genes in epinephrine-treated cells showed that *SLUG*, a key regulatory factor in breast cancer stemness (25), exhibited the greatest increase in cells following epinephrine treatment (Figure 2B). Using 3 different inhibitory shRNAs, we found that ablation of SLUG decreased expression of the stemness proteins β-catenin, OCT-4, and NANOG (Supplemental Figure 3B). Consistently, depletion of SLUG dramatically reversed epinephrine-improved mammosphere formation ability (Figure 2C and Supplemental Figure 3C) as well as chronic stress–induced tumor progression (Figure 2D). SLUG knockdown also inhibited the epinephrine-induced increased expression of β-catenin, OCT-4, and NANOG (Supplemental Figure 3D). These data suggest that SLUG plays a key role in the maintenance of stress-induced breast cancer stem-like properties.

To explore how *SLUG* mRNA is regulated, we evaluated the half-life of *SLUG* mRNA in the presence and absence of epinephrine. We found that epinephrine had no significant effect on the half-life of *SLUG* mRNA (Supplemental Figure 3E). Then, using a luciferase reporter assay, we found that the *SLUG* promoter (–2121 to 0 bp) is activated by epinephrine. Serial truncated analysis of the *SLUG* promoter (–1497 to 0, –997 to 0, –496 to 0 bp) revealed that the –496 to 0 bp region of the *SLUG* promoter is required for epinephrine-mediated *SLUG* transactivation (Figure 2E). Furthermore, by analysis of the *SLUG* promoter sequence (–496 to 0 bp) using the JASPAR database (Supplemental Figure 3F), MYC was identified to be the most probable candidate responsible for *SLUG* transactivation. As expected, overexpression or depletion of MYC remarkably increased and decreased, respectively, both *SLUG* mRNA and SLUG protein expression (Supplemental Figure 3, G and H). *SLUG* promoter activity was dramatically enhanced or suppressed following up- or downregulation of MYC (Supplemental Figure 3I). In concordance with these findings, activity of the truncated *SLUG* promoter (–496 to 0 bp) was enhanced upon epinephrine exposure, but it was reversed upon MYC knockdown (Figure 2F).

To further define the binding sites of MYC in transactivating *SLUG*, we constructed 3 MYC-responsive element mutants, –57/–54 (Mut1, CGTG to TTTT), –104/–101 (Mut2, CACG to TTTT), and –412/–408 (Mut3, CGTGG to TTTTT), and subcloned them into a luciferase vector. Luciferase reporter assays showed that the responsive element corresponding to Mut3 was responsible for induction of MYC by epinephrine (Figure 2G and Supplemental Figure 3J). We performed chromatin immunoprecipitation (ChIP) assays using a MYC antibody and confirmed that MYC directly binds to the –496 to –394 region of the endogenous *SLUG* promoter, whereas there was no binding in the –394 to 0 region of the *SLUG* promoter (Figure 2H). These findings demonstrate that MYC directly binds to the *SLUG* promoter and transactivates its expression under epinephrine treatment (Figure 2I).

### USP28 directly deubiquitinates and stabilizes MYC.

**Figure 3:**
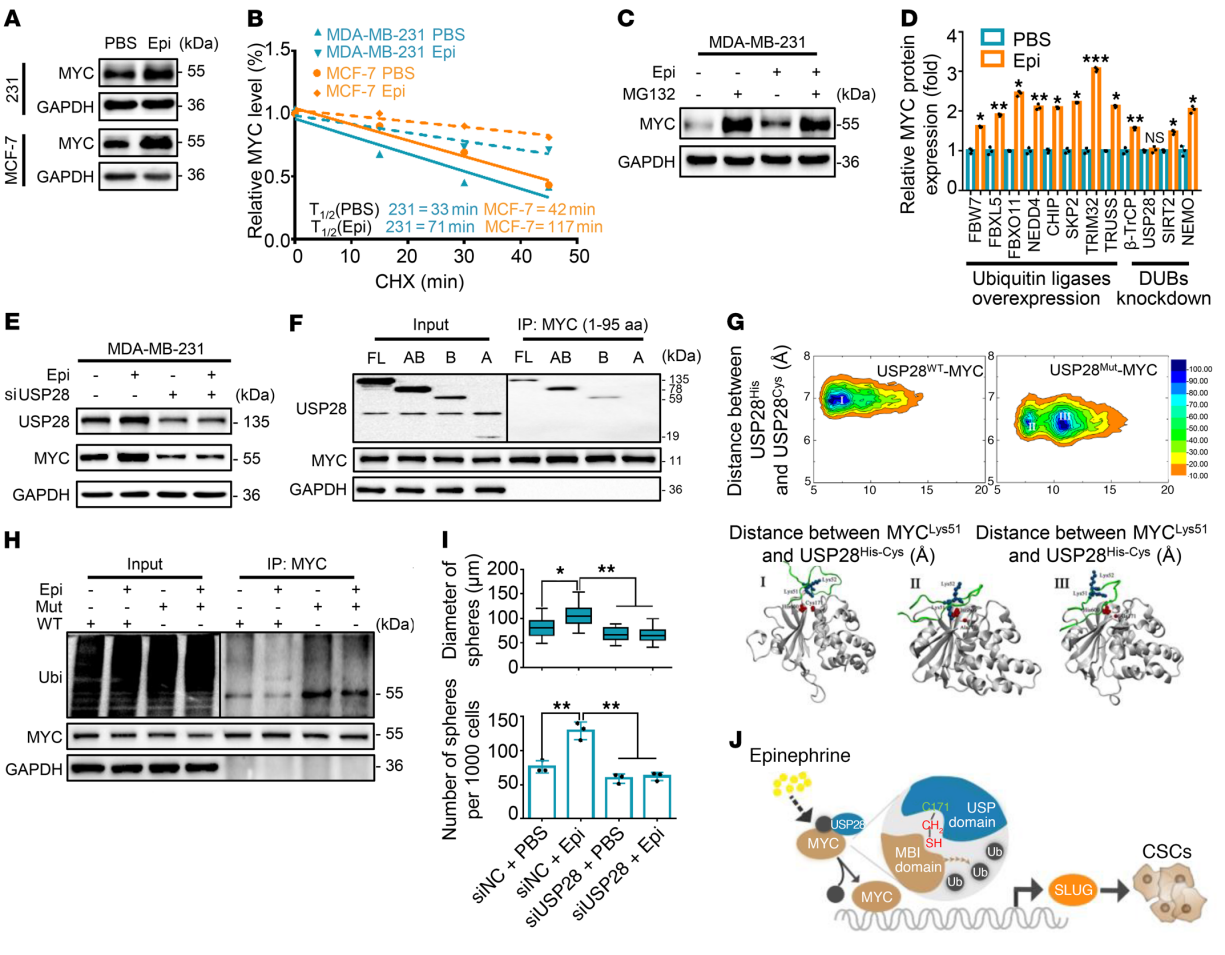
Chronic stress–induced USP28 stabilizes MYC and promotes CSCs. (**A**) Immunoblots of MYC in MDA-MB-231 and MCF-7 cells treated for 5 days with PBS or Epi. (**B**) MDA-MB-231 cells were treated with Epi for 5 days followed by treatment with cycloheximide (CHX) for the indicated times. The intensity of MYC expression for each time point was quantified by densitometry and plotted against time. (**C**) Immunoblots of MDA-MB-231 cells treated with Epi for 5 days and then incubated with or without MG132 for 6 hours. (**D**) Fold change of immunoblots of MDA-MB-231 cells transfected with indicated plasmids followed by treatment with Epi for 5 days; *n* = 3 (Student’s *t* test). (**E**) Immunoblot analysis of MDA-MB-231 cells transfected with USP28 siRNA-2 followed by treatment with PBS or Epi for 5 days. (**F**) Immunoprecipitation of USP28 constructs and MYC (amino acids 1–95) in 293T cells. (**G**) Free-energy surface of the USP28^WT^-MYC^46–74^ complex and USP28^Mut^-MYC^46–74^ complex (top panel). Gray cartoons, USP28^WT/Mut^ structures; red spheres, side chain of His^600^ and Cys/Ala^171^; green cartoons, MYC motifs; blue spheres, Lys^51^ and Lys^52^ in MYC motif. (**H**) Ubiquitin assays of 293T cells transfected with MYC and WT or a C171A mutant (Mut) of USP28 followed by treatment with Epi. (**I**) Distribution patterns and number (d > 50 μm) of mammospheres from cells in **E**; *n* = 3 (1-way ANOVA). (**J**) Model of Epi-induced cancer stem-like traits through USP28/MYC/SLUG signaling. Data are representative of at least 3 independent experiments. Data represent mean ± SEM; *P < 0.05, **P < 0.01, ***P < 0.001.

To examine the effects of chronic stress–induced epinephrine on MYC expression, we analyzed *MYC* mRNA and protein levels following epinephrine treatment for 5 days. Epinephrine led to a significant increase in MYC protein (Figure 3A) but revealed no change in *MYC* mRNA (Supplemental Figure 4A). We next treated cells with the protein synthesis inhibitor cycloheximide to determine whether epinephrine regulates MYC protein degradation. Indeed, MYC protein levels exhibited a gradual decrease in the absence of epinephrine, whereas MYC degradation was significantly attenuated in the presence of epinephrine (Figure 3B and Supplemental Figure 4B). These results indicate that epinephrine enhances the stability of MYC protein.

As rapid MYC protein turnover can be mediated by the ubiquitin-dependent proteasome pathway (26, 27), we treated cells with the proteasome inhibitor MG132 in the presence of epinephrine. Interestingly, MG132 enhanced MYC expression, whereas epinephrine did not obviously increase MYC level under MG132 treatment (Figure 3C), suggesting that epinephrine stabilizes MYC through inhibition of the proteasome-dependent degradation pathway. We next coexpressed His-MYC and HA-ubiquitin in 293T cells, followed by treatment with epinephrine in the presence or absence of MG132. MYC was heavily ubiquitinated in MG132-treated cells, but was significantly reduced following epinephrine treatment (Supplemental Figure 4C). These results suggest that epinephrine stabilizes MYC by eliminating its ubiquitination and consequent degradation.

MYC ubiquitination is a dynamic process involving ubiquitin ligases and deubiquitinases (DUBs) (28). We identified the deubiquitinase USP28 as a key candidate that reverses epinephrine-enhanced MYC expression by overexpressing E3 ligases or knockdown of DUBs (Figure 3D). As expected, depletion of USP28 remarkably decreased MYC protein and reversed epinephrine-induced increase in MYC protein expression (Supplemental Figure 4D and Figure 3E), whereas overexpression of USP28 enhanced MYC expression (Supplemental Figure 4E). We further verified that USP28 knockdown reduced epinephrine-induced MYC stabilization and directly bound to MYC box I through its USP domain without FBW7 (Figure 3F and Supplemental Figure 4, F–K).

To further investigate the functional impact of mutant USP28 (USP28^Mut^) in which the catalytic cysteine has been replaced by alanine (C171A), different models of binding between USP28^WT^ or USP28^Mut^ and MYC^46–74^ amino acids were assessed using macromolecular modeling and molecular dynamics (MD) simulation approaches (29). We obtained conformational free-energy surfaces of USP28^WT^ and USP28^Mut^ with the MYC motif using fully atomistic explicit-solvent force fields. This finding indicates a more favorable binding interaction between USP28^WT^ and MYC than between USP28^Mut^ and MYC (Figure 3G).

This result is also confirmed by a distance diagram for the USP28^WT/Mut^ catalytic domain following MD simulation for 150 nanoseconds (Supplemental Figure 4, L–N). In addition, the root-mean-square fluctuation profiles demonstrated greater fluctuations in the USP28^WT^ than in the mutant USP28^Mut^, a finding that suggests more favorable binding of USP28^WT^ to the MYC^46–74^ motif (Supplemental Figure 4O). These results explained how a single mutation in USP28 leads to a rather unfavorable binding between USP28^Mut^ and MYC, indicating that the Cys^171^ residue of USP28 is critical for binding MYC (Supplemental Videos 1 and 2). We then cotransfected USP28^WT^ or USP28^Mut^ along with His-MYC and HA-ubiquitin into 293T cells. Ubiquitination assays showed that USP28^Mut^ markedly increased MYC ubiquitination level compared with USP28^WT^ and reversed the epinephrine-inhibited ubiquitin level of MYC (Figure 3H). In agreement, USP28 knockdown decreased mammosphere diameter and number (Figure 3I and Supplemental Figure 4P). Together, these findings illustrate that epinephrine stabilizes MYC protein by inducing USP28 and that USP28 Cys^171^ directly interacts with the MYC MBI domain (Figure 3J).

### Chronic stress recruits glycolytic activator LDHA to promote glucose metabolic rewiring.

**Figure 4:**
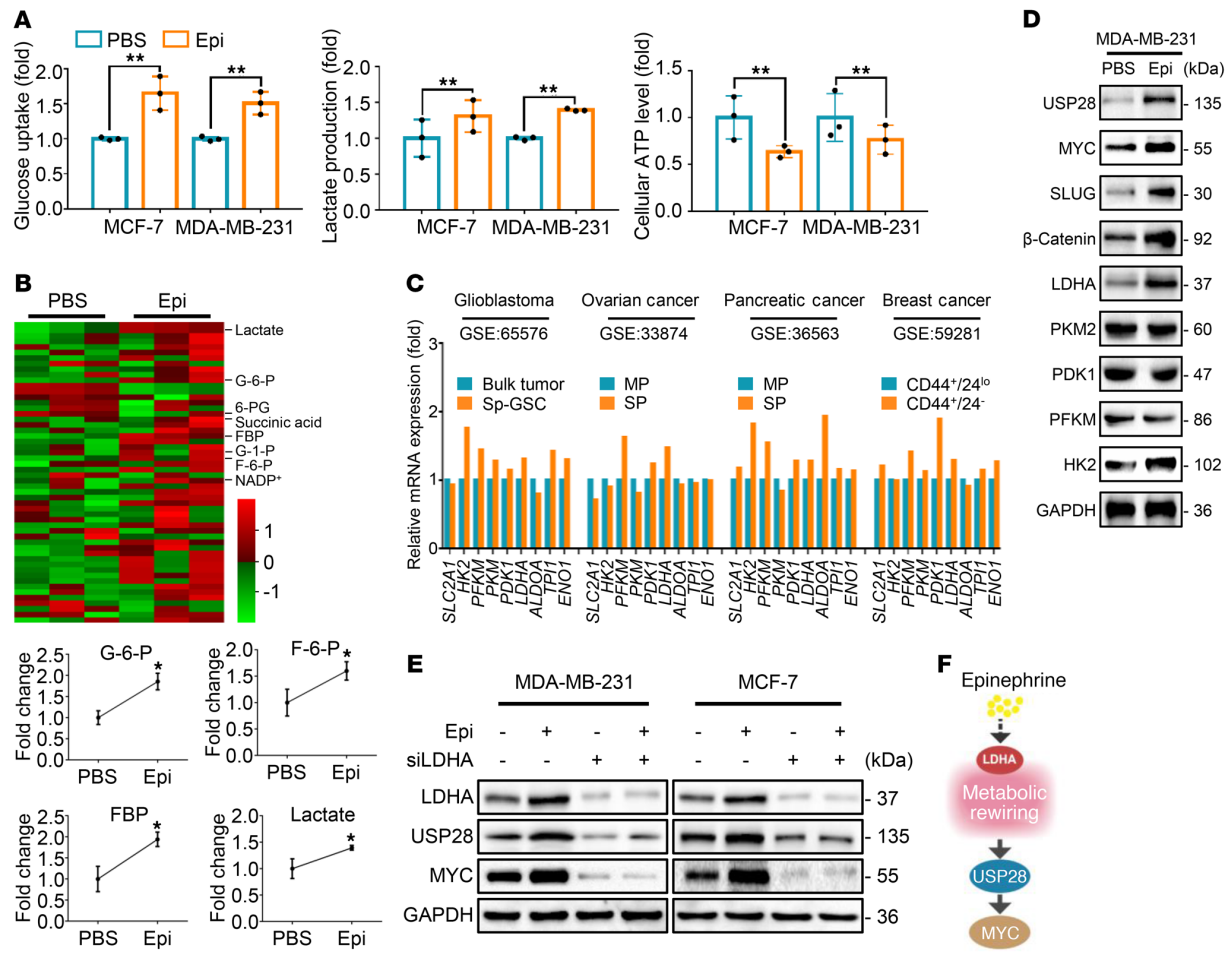
Chronic stress elevates LDHA to enhance glycolysis. (**A**) Glucose uptake, lactate production, and cellular ATP were measured in MCF-7 and MDA-MB-231 cells treated with PBS or Epi for 5 days; *n* = 3 (Student’s *t* test). (**B**) Representative heatmap of metabolome profiles (top panel). Heatmap colors represent relative metabolite levels as indicated in the color key. Average fold change of glycolytic metabolites was measured by capillary electrophoresis–mass spectrometry (bottom). G-6-P, glucose-6-phosphate; F-6-P, fructose-6-phosphate; FBP, fructose-1,6-phosphate. *n* = 3 (Student’s *t* test). (**C**) Relative mRNA expression of indicated genes in 4 GEO databases analyzed by GEO2R. (**D**) Immunoblot analysis of MDA-MB-231 cells treated with PBS or Epi for 5 days. (**E**) Immunoblot analysis of MDA-MB-231 and MCF-7 cells transfected with siLDHA in the presence or absence of Epi for 5 days. (**F**) Model of Epi-induced USP28/MYC signaling through LDHA-mediated metabolic rewiring. Data are representative of at least 3 independent experiments. Data represent mean ± SEM; *P < 0.05, **P < 0.01.

Metabolic reprogramming toward aerobic glycolysis and biomass accumulation is known to accompany tumorigenesis (30). We therefore decided to study the role of glucose metabolism following chronic stress. Epinephrine-treated cells increased glucose and lactate levels and decreased cellular ATP compared with control cells (Figure 4A). Epinephrine-treated cells exhibited an increased extracellular acidation rate and decreased oxygen consumption rate (Supplemental Figure 5A). Moreover, using targeted capillary electrophoresis–mass spectrometry (CE-MS), we extracted 54 metabolites and found a significant increase in the levels of glycolytic metabolites in epinephrine-treated compared with PBS-treated cells (Figure 4B and Supplemental Figure 5B). To further explore the differentially expressed genes of glycolysis enzymes in CSCs, we analyzed 4 publicly accessible Gene Expression Omnibus (GEO) data sets of different cell models with replicates. We discovered that glycolysis-associated genes were enriched in a cancer stem cell population (Figure 4C). These data suggest that epinephrine switches glucose metabolism from homeostasis to glycolysis.

To examine the underlying cause for this switch, expression of several key glycolytic enzymes following epinephrine treatment were examined, including HK2, PFKM, PKM2, LDHA, and PDK1. We observed that both HK2 and LDHA increased in response to epinephrine (Figure 4D and Supplemental Figure 5C). However, norepinephrine promoted the expression of PFKM and HK2, but not USP28, MYC, or SLUG. In addition, cortisol had no effect on these key regulators (Supplemental Figure 5D). Also, high glucose consistently triggered HK2 and LDHA expression and stimulated the USP28/MYC axis in breast cancer cells (Supplemental Figure 5E). Silencing of LDHA significantly reversed induction of USP28 and MYC by epinephrine (Figure 4E), while silencing of HK2 displayed no change on the effect of epinephrine (Supplemental Figure 5F). Together, these findings illustrate that epinephrine enhances USP28 expression via induction of LDHA and metabolic rewiring (Figure 4F).

### LDHA generating lactate enhances the USP28 signaling.

**Figure 5:**
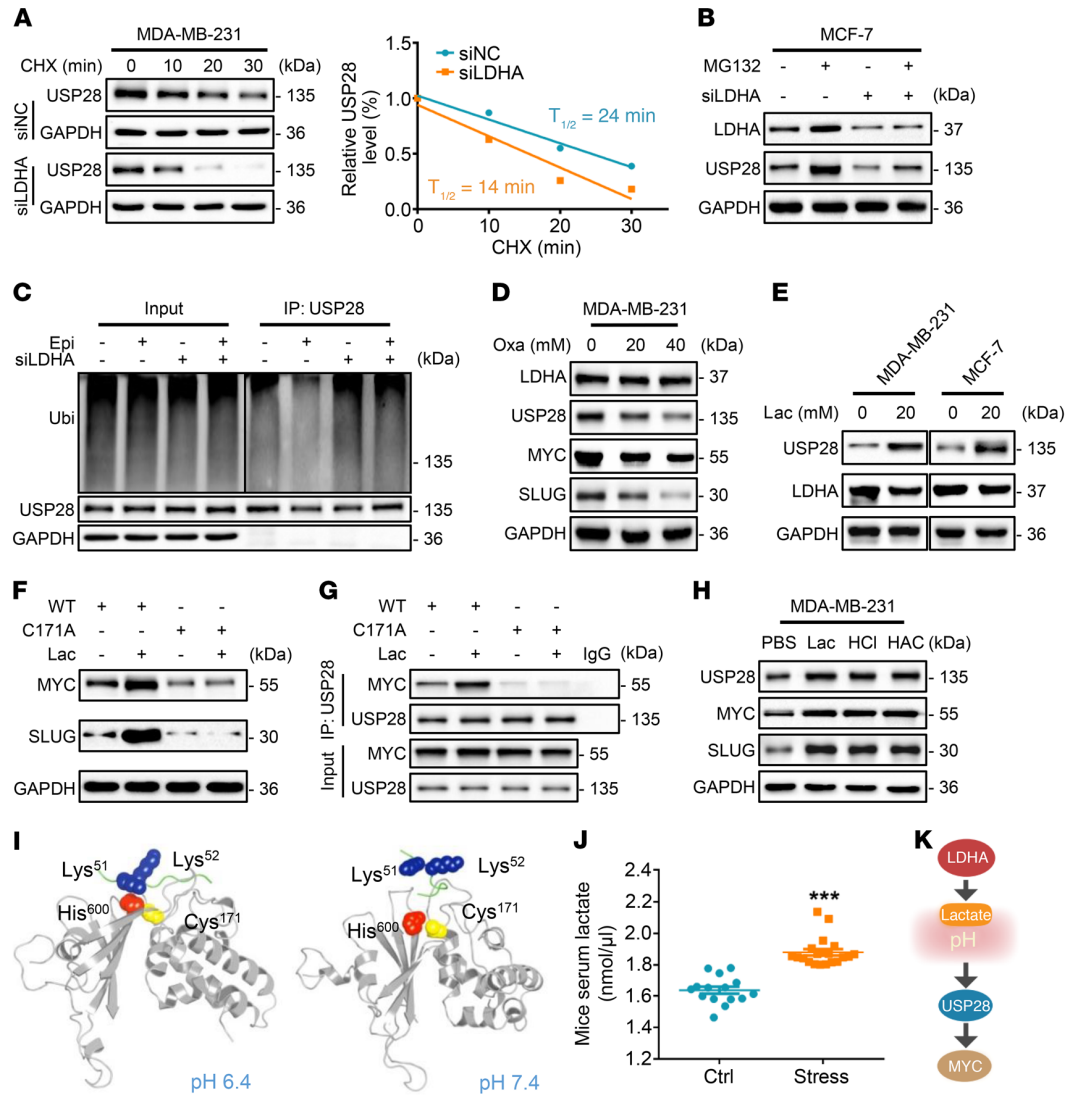
LDHA generating lactate enhances the USP28 signaling. (**A**) Immunoblots of siNC and siLDHA MDA-MB-231 cells treated with CHX for the indicated times. Intensity of USP28 expression for each time point was quantified by densitometry and plotted against time (right panel). (**B**) Immunoblots of MCF-7 cells treated for 5 days with Epi and/or siLDHA followed by incubation with or without MG132 for 6 hours. (**C**) Ubiquitin assays of 293T cells transfected with ubiquitin (Ubi) and siLDHA followed by treatment with Epi. (**D**) Immunoblots of MDA-MB-231 cells treated with the indicated concentrations of sodium oxamate (Oxa) for 48 hours. (**E**) Immunoblots of MDA-MB-231 and MCF-7 cells treated with lactate (Lac) for 72 hours. (**F**) Immunoblots of MDA-MB-231 cells transfected with USP28 WT or C171A and then treated with lactate for 72 hours. (**G**) Immunoblots of immunoprecipitation experiments of 293T cells transfected with USP28 WT or C171A and then treated with lactate for 72 hours. (**H**) MDA-MB-231 cells were treated with lactate, hydrochloric acid (HCl), and acetic acid (HAC) for 72 hours. Expression of the indicated proteins was examined by immunoblotting. (**I**) Snapshot structures of USP28 interacting with MYC motif extracted from constant-pH MD simulations at 2 representative pH conditions. Gray cartoons, USP28; green cartoons, MYC; blue spheres, Lys^51^ and Lys^52^ on MYC motif; red spheres and yellow spheres, His^600^ and Cys^171^ of USP28, respectively. (**J**) Lactate levels in serum of Ctrl (*n* = 15) or stress (*n* = 20) mice (Student’s *t* test). (**K**) Model of chronic stress–mediated USP28 stabilization through decreased pH caused by LDHA generating lactate. Data are representative of at least 3 independent experiments. Data represent mean ± SEM; ***P < 0.001.

To examine the effects of chronic stress–induced epinephrine on USP28 expression, we conducted quantitative PCR and found that LDHA knockdown had no significant effect on *USP28* mRNA expression (Supplemental Figure 6A). To test the possibility that LDHA affects USP28 stability, we treated cells with cycloheximide and found that the half-life of USP28 was shortened in LDHA-depleted cells (Figure 5A). Moreover, the reduction in expression of USP28 caused by LDHA knockdown was reversed by MG132 (Figure 5B). LDHA ablation also rescued USP28 ubiquitination that was inhibited by epinephrine (Figure 5C). Following treatment with the LDHA inhibitor sodium oxamate, we found a reduction in USP28 stabilization induced by LDHA in a dose-dependent manner (Figure 5D and Supplemental Figure 6B).

To explore whether LDHA stabilizes USP28 expression through its major product lactate, we treated cells with lactate and found that it substantially increased USP28 protein (Figure 5E) and prolonged the half-life of USP28 (Supplemental Figure 6C). Importantly, USP28^WT^ remarkably increased protein expression of MYC, whereas USP28^Mut^ reversed lactate-induced MYC and SLUG (Figure 5F). Furthermore, we found that lactate enhanced the interaction of MYC only with USP28^WT^. However, there was no interactive effect when the USP28^Mut^ was tested (Figure 5G and Supplemental Figure 6D). Interestingly, the lactate-induced weak acidic environment promoted USP28 signaling as well as hydrochloric acid and acetic acid (Figure 5H). Moreover, the pH 6.4 condition provides a closer distance between the MYC motif and the USP28 catalytic domain than a neutral pH 7.4. This finding implicates a much more stable binding and more efficient deubiquitination in a weak acidic environment (Figure 5I, Supplemental Figure 6E, and Supplemental Video 3). In accord with these data, the concentration of serum lactate was significantly higher in stressed mice compared with control mice (Figure 5J). Collectively, these data suggest that lactate enhances the USP28-MYC interaction via generation of a local acidic microenvironment (Figure 5K).

### High serum epinephrine is associated with poor prognosis and activated LDHA/USP28/MYC/SLUG signaling axis in breast cancer patients.

**Figure 6:**
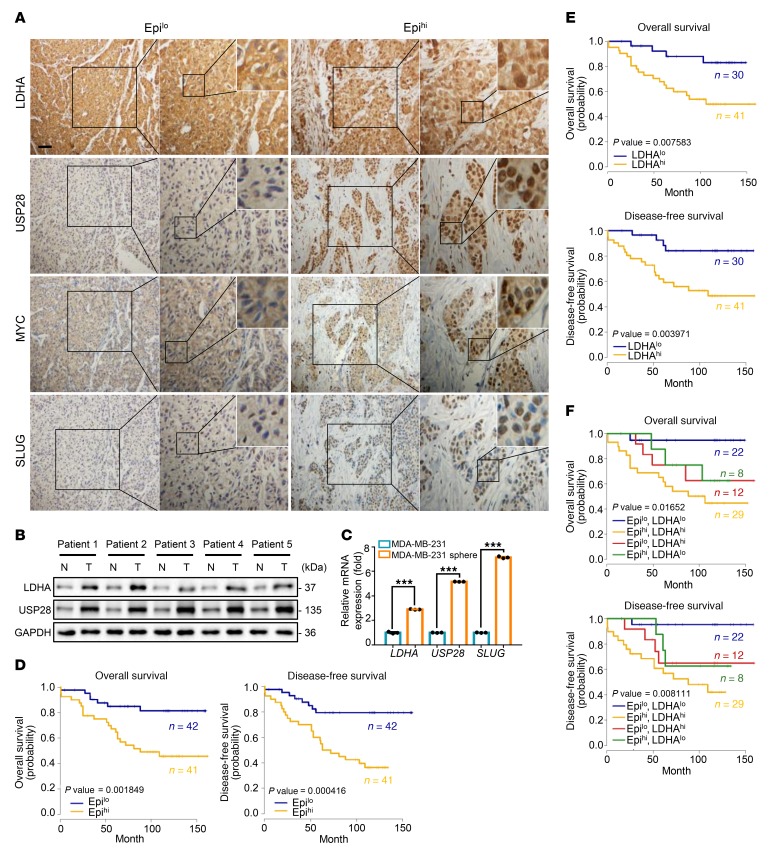
Clinical relevance of LDHA expression under chronic stress. (**A**) Representative immunohistochemistry photomicrographs of tissues stained with indicated antibodies in patients with breast cancer (Epi^lo^, *n* = 42; and Epi^hi^, *n* = 41). Scale bar: 50 μm; original magnification, ×20, ×40, ×96 (enlarged insets). (**B**) Immunoblot analysis of proteins in breast cancer tissues (T) and adjacent normal breast tissues (N); *n* = 5. (**C**) Expression of mRNA for the indicated genes in MDA-MB-231-2D cells or spheres was measured by quantitative reverse transcriptase PCR; *n* = 3 (Student’s *t* test). (**D**) Kaplan-Meier estimates of overall survival and disease-free survival of patients with breast cancer, according to the serum Epi concentrations (Epi^lo^, *n* = 42; and Epi^hi^, *n* = 41). Eighty-three patients were in the data set (log-rank test). (**E**) Kaplan-Meier estimates of overall survival and disease-free survival of patients with breast cancer, according to LDHA expression (LDHA^lo^, *n* = 30; and LDHA^hi^, *n* = 41). Seventy-one patients were in the data set (log-rank test). (**F**) Kaplan-Meier estimates of overall survival and disease-free survival of patients with breast cancer expressing high or low LDHA together with high or low serum Epi. Seventy-one patients were in the data set (log-rank test). Data are representative of at least 3 independent experiments. Data represent mean ± SEM; ***P < 0.001.

To evaluate the clinical relevance of circulating epinephrine, we studied the relationship between epinephrine and clinical pathological parameters in 83 breast cancer patients using serum samples and paraffin-embedded tissues, respectively. We first determined serum epinephrine concentrations by ELISA and divided the patients into 2 groups, Epi^lo^ and Epi^hi^, based on receiver operating characteristic (ROC) curve analysis (Supplemental Figure 7A). The relationship between serum epinephrine levels and clinical pathological parameters of breast cancer patients were then analyzed (Table 5). High epinephrine levels were not significantly correlated with tumor stage, node stage, or other clinical pathological characteristics (HER2, ER, and PR) (Table 5). Immunohistochemical staining was performed on these tissues, and the results revealed that high serum epinephrine was positively associated with high LDHA, USP28, MYC, and SLUG protein expression (Figure 6A and Supplemental Figure 7B). We next collected 5 pairs of breast cancer and adjacent normal tissues and conducted Western blot analysis. All breast cancer tissues displayed higher LDHA and USP28 protein levels when compared with adjacent normal tissues (Figure 6B and Supplemental Figure 7C). In addition, quantitative PCR analysis showed that MDA-MB-231 sphere–enriched cells displayed higher *LDHA-USP28-SLUG* expression compared with MDA-MB-231-2D cells (Figure 6C). These findings suggest that high serum epinephrine is positively related to LDHA/USP28/MYC/SLUG signaling and that LDHA can be a potential independent prognostic factor for breast cancer.

Patients with high serum epinephrine exhibited lower overall survival (OS) rate and disease-free survival (DFS) rate compared with patients with low epinephrine levels (Figure 6D). Meanwhile, Cox regression analysis showed that low serum epinephrine is a significant predictor of both longer OS and DFS (Table 6). We then divided 71 breast cancer samples into 2 groups, LDHA^lo^ and LDHA^hi^, by immunohistochemistry grade (data not shown) based on the ROC curve analysis (Supplemental Figure 7D). As predicted, the LDHA^hi^ group showed lower OS and DFS rates compared with the LDHA^lo^ group (Figure 6E). Notably, the Epi^lo^ and LDHA^lo^ groups displayed a more favorable prognosis than the Epi^hi^ and LDHA^hi^ groups, supporting the significant correlation between serum epinephrine levels and LDHA expression (Figure 6F).

### Vitamin C is a promising intervention for breast cancer patients with chronic stress.

**Figure 7:**
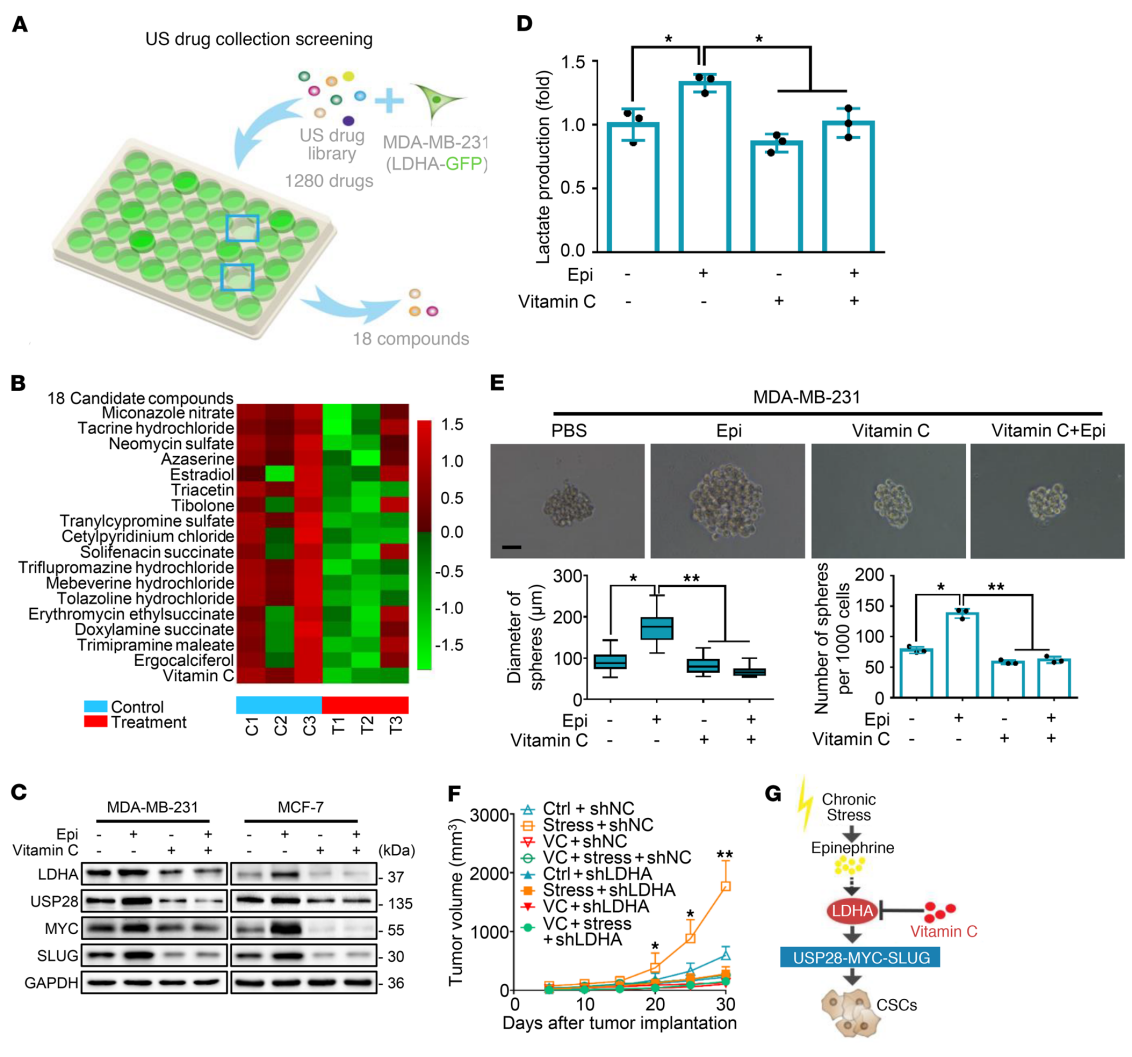
Vitamin C reverses chronic stress–induced breast cancer stem-like properties in vivo and in vitro. (**A**) US drug collection screening: MDA-MB-231 cells stably expressed with pEGFP-LDHA were treated with 1280 drugs. Fluorescence intensities were examined. *n* = 3. (**B**) Representative heatmap of LDHA expression after treatment with the 18 candidate compounds; *n* = 3. Heatmap colors represent relative LDHA protein levels as indicated by the color key. (**C**) Immunoblot analysis of MDA-MB-231 and MCF-7 cells treated with vitamin C and/or Epi for 5 days. (**D**) Lactate levels were examined in the cell culture media of MDA-MB-231 cells in **C**; *n* = 3 (Student’s *t* test). (**E**) Representative spheroid images formed by single cells with vitamin C and/or Epi; *n* = 3. Scale bar: 50 μm. Bottom left panel shows distribution patterns of sphere diameter. Bottom right panel shows the number of spheres (d > 50 μm) (1-way ANOVA). (**F**) Tumor growth curves of indicated treatments of mice; *n* = 5 (1-way ANOVA). (**G**) Model of targeting chronic stress–mediated cancer stem-like traits by vitamin C. Data are representative of at least 3 independent experiments. Data represent mean ± SEM; *P < 0.05, **P < 0.01.

To identify a potential therapeutic agent for patients undergoing chronic stress, we conducted a screen based on the US drug collection of compounds. To this end, MDA-MB-231 cells stably expressing the EGFP-LDHA fusion protein were incubated with different compounds for 6 (1 μM or 2 μM) or 12 hours (1 μM) (Figure 7A). The screening identified 18 compounds that lowered fluorescence of EGFP-LDHA, including vitamin C (Figure 7B and Supplemental Figure 8A). Virtual screening of 2037 FDA-approved drugs against LDHA also revealed that vitamin C was among the 7 vitamins in the top 200 hits (Supplemental Figure 8B). Furthermore, vitamin C has no effect on cell viability at the experimental doses and time courses used (Supplemental Figure 8C). We next examined the impact of vitamin C on epinephrine-induced LDHA/USP28/MYC/SLUG signaling by Western blot analysis and found that vitamin C attenuated the epinephrine-induced increase in LDHA, USP28, MYC, and SLUG expression (Figure 7C).

The potential impact of vitamin C on LDHA activity was then examined. We found that vitamin C suppressed lactate production in both the absence and the presence of epinephrine (Figure 7D and Supplemental Figure 8D). In addition, vitamin C significantly caused a similar inhibition of BCSCs in the sphere formation assay (Figure 7E). Next, we injected vitamin C to determine whether it would inhibit tumor progression caused by chronic stress. Compared with the control stressed group, mice treated additionally with vitamin C showed a significant reduction in tumor volume (Figure 7F and Supplemental Figure 8E). We also generated an MDA-MB-231 shLDHA cell line for tumor formation assays. Interestingly, knockdown of LDHA had an effect similar to that of vitamin C treatment, in which the stressed mice displayed an obvious reduction in tumor volume compared with untreated controls (Figure 7F). Taken together, these findings show that lowering of LDHA by vitamin C reduces tumorigenicity and that vitamin C might be a novel and effective therapeutic agent for targeting cancer in patients undergoing chronic stress (Figure 7G).

## Discussion

Chronic stress is associated with aberrantly persistent activation of the hypothalamic-pituitary-adrenal axis, leading to enhanced production of cortisol and the simultaneous elevation of catecholamines (23). We find that chronic stress promotes tumor progression in both immunocompetent and immunocompromised mouse models. Furthermore, using a NOD/SCID mouse model, we show that chronic stress increases epinephrine levels and activates β_2_-adrenergic receptor to promote breast cancer stem-like properties via metabolic rewiring. Chronic stress–induced epinephrine enhances LDHA-dependent metabolic activity, which increases lactate and augments USP28 that serves to stabilize the MYC protein. The data further revealed that MYC transactivates the *SLUG* promoter to enhance breast cancer stem-like traits. In a drug screen that targeted LDHA, we identified vitamin C as an agent capable of reversing the chronic stress–induced cancer stem-like phenotype. These findings demonstrate the involvement of psychological factors in promoting stem-like properties in breast cancer cells and promoting their tumorigenic potential. The mechanism is mediated by an LDHA-mediated glycolysis-dependent pathway. Importantly, we suggest that vitamin C, which targets this pathway by inhibiting LDHA, is a potential treatment for the stress-associated increase in breast cancer.

A substantial body of literature describes the effect of chronic stress on tumor progression. Stress-induced hormones control a number of important biological processes, such as metabolic events, immune activity, and apoptosis. Cortisol, a key stress hormone, improves the hypoglycemic profile by promoting gluconeogenesis (31) and endoplasmic glucose production via pyridine nucleotide redox reactions (32). Similarly, another vital stress hormone, epinephrine, stimulates glycogenolysis by activating glycogen synthase and increasing insulin-stimulated glucose uptake (33). In our experiments, epinephrine-treated cells consistently increased glucose and lactate levels and reduced cellular ATP levels. Furthermore, chronic exposure to epinephrine promotes the establishment of immunosuppressive microenvironments through the induction of a COX2-dependent pathway (34). Epinephrine can also enhance antiapoptotic functions through cAMP-dependent phosphorylation of BAD (35). In addition, chronic stress facilitated tumor angiogenesis through β-adrenergic activation of the cAMP/PKA signaling pathway in vivo and thereby promoted tumor growth (5). Stress-induced hormones, especially norepinephrine and epinephrine, protected the ovarian tumor cells from anoikis and promoted their proliferation by phosphorylating focal adhesion kinase (FAK) at Y397 in vitro and in vivo (36). Consistently, we demonstrate that chronic stress increases epinephrine levels to promote tumorigenesis and cancer stem-like traits via activation of the LDHA/USP28/MYC/SLUG signaling axis in a mouse model.

Our data showed that high epinephrine in patient serum was positively associated with high LDHA, USP28, MYC, and SLUG expression and conferred lower overall survival and disease-free survival rates compared with those of patients with low epinephrine levels. In addition, epinephrine levels are not constant and can be influenced by activities such as exercise. Tumor-bearing mice with access to running wheels showed reduced growth of ER-positive breast tumors via activation of the Hippo signaling pathway, a known regulator of cancer stem cells (37). The exercise-induced epinephrine surge and IL-6 suppress tumor growth and development through NK cell mobilization and redistribution (38, 39). These studies reveal that high-intensity exercise markedly upregulates epinephrine to an extremely high level, which may lead to suppression of cancer. Hence, the comprehensive influence on epinephrine levels should be carefully evaluated in the further patient outcome and drug intervention. Our results also show that norepinephrine but not cortisol moderately enhanced the expression of self-renewal regulators (NANOG, OCT-4, and β-catenin) and glycolysis enzymes (PFKM and HK2). The mechanism by which norepinephrine contributes to cancer stem-like properties and glucose metabolism is worth exploring further.

The transcription factor SLUG is a member of the Snail family that is essential for embryonic development (40, 41). Emerging evidence demonstrates that SLUG plays an essential role in metastasis due to its endogenous overexpression in a variety of cancers (42, 43). SLUG is also a key protein that controls cancer cell stemness (44). Using Affymetrix Human PrimeView to perform large-scale expression profile analysis followed by comparison with the array data (fold change >2, *Q* < 0.05) with a stem cell gene set, we found that *SLUG* expression was significantly increased in mice treated with epinephrine. MYC activates a diverse group of genes that are known to promote cell growth and proliferation as part of a heterodimeric complex with the protein MAX. The MYC-MAX heterodimer is capable of binding specific DNA sequences, such as the E-box sequence CACGTG (45, 46). In our studies, analysis through the JASPAR database first confirmed that MYC activates *SLUG* transcription to stimulate CSCs. These results are consistent with the previous finding that MYC binds directly to the *SLUG* DNA sequence –412 to –408 to transactivate its expression in breast cancer.

An unbiased search screen has been reported for MYC, from which we determined that the deubiquitinase USP28 directly stabilizes the MYC protein in our chronic stress system. USP28 is a USP member of the DUB family, which has 4 known domains. Previous studies have shown that USP28 does not bind to MYC directly but rather binds MYC through interaction with FBW7 (47). Emerging evidence demonstrates that USP28 can bind and promote deubiquitination of MYC in the absence of FBW7 in intestinal crypt stem cells (29). Importantly, our results establish that the MYC MBI domain (1–95 amino acids) interacts directly with the USP domain (160–652 amino acids) of USP28. Its crystal structure showed that USP28 interacts with MYC through C171 and the MBI domain. Moreover, recent findings show the ubiquitination sites of MYC, including Lys 51, Lys 148, Lys 389, and Lys 430, in response to DNA damage using quantitative proteomics (48). Our computational simulation analysis also revealed that USP28 displayed possible binding to Lys 51 and Lys 52 of MYC, which implied that ubiquitination might be involved in USP28’s binding to MYC.

Cancer cells show profound metabolic changes, mainly comprising aerobic glycolysis, de novo lipid biosynthesis, and glutamine-dependent anaplerosis, all of which provide energy and building blocks to sustain their high proliferation rates (49). The first noted change in cancer metabolism was in aerobic glycolysis, known as the Warburg effect. It is characterized by the ATP generation pattern shifting from oxidative phosphorylation to glycolysis, even under normal oxygen concentrations (50). This effect is regulated by PI3K, hypoxia-inducible factor (HIF), p53, MYC, and AMP-activated protein kinase (AMPK)/liver kinase B1 (LKB1) pathways (51). To analyze whether glycolysis is involved in the effects of epinephrine on breast cancer cells, we used CE-MS to examine changes in metabolites in stressed cells. This approach revealed a significant enrichment in metabolic processes. In glycolysis, LDHA executes the final step of aerobic glycolysis signaling by converting pyruvate to lactate. Deregulation of LDHA has been reported in many cancers (18–20, 52), but the underlying biochemical process by which LDHA acts on cancer cells has been unknown. Our results reveal that LDHA induces glucose metabolic disorders and stabilizes USP28 expression. However, the detailed mechanism by which LDHA stabilizes USP28 expression needs to be further explored.

The LDHA inhibitor sodium oxamate is known to be an effective anticancer agent in many types of cancer, including breast (53–56), non–small cell lung (57), and gastric cancer (58). However, the clinical application of sodium oxamate has been limited because of its high therapeutic doses and relatively low potency (59–61). As a result, new medications are urgently needed to replace sodium oxamate for targeting LDHA and to reverse the chronic stress–derived breast cancer stem-like phenotype. Using an FDA-approved high-throughput drug screen that includes 1280 drugs, we identified vitamin C as one of 18 molecules that lowered LDHA expression. Recent reports support our findings that vitamin C is an effective anticancer agent. For example, high levels of vitamin C can selectively kill KRAS- and BRAF-driven colorectal cancer cells by inducing oxidative stress, suppressing glycolysis and the subsequent energy crisis (62). High-dose intravenous vitamin C also promotes cell death through depletion of NAD^+^ and inhibits motility and mitosis by increasing α-tubulin acetylation in pancreatic cancer (63). In addition, physiological-level vitamin C, acting as an antioxidant, increases the activity of TET enzymes to rebuild 5-hydroxymethylcytosine (5hmC) content, leading to the inhibition of invasiveness and clonogenic growth in melanoma (64). Low-dose vitamin C as a cofactor of TET enhances the apoptosis effect of DNMT inhibitors via enhancing immune signals in the treatment of hematological cancers (65). It is also noteworthy that vitamin C–treated stressed mice display a significant reduction in depressive-like behaviors (66). Furthermore, vitamin C specifically targets breast cancer stem cells via suppression of glycolysis due to loss of mitochondrial function (67). Vitamin C counteracts miR-302/367 to suppress breast cancer stem cell reprogramming via decreasing TET1 gene expression (68). Although a recent study indicated that high-dose vitamin C selectively induced DNA damage on glioblastoma stem cells rather than differentiated tumor cells, it also displayed the cellular toxicity on neural stem and progenitor cells (69). In our study, vitamin C suppressed stress-induced LDHA, thus altering the lactate production to inhibit the USP28/MYC/SLUG axis in breast cancer stem cells.

## Methods

### Cell lines and culture conditions.

Human breast cancer cell lines (MDA-MB-231 and MCF-7) and 293T were purchased from the American Type Culture Collection (ATCC). These cell lines were authenticated at ATCC before purchase by standard short tandem repeat DNA-typing methodology. The murine mammary carcinoma cell line E0771 was purchased from BeNa Culture Collection. The Py8119 cell line was provided by Suling Liu (Fudan University, Shanghai, China). The MDA-MB-231, E0771, and 293T cell lines were maintained in Dulbecco’s modified Eagle medium (DMEM; Invitrogen) supplemented with 10% FBS (Invitrogen Corp.). The MCF-7 cell line was maintained in Eagle’s minimum essential medium (Invitrogen Corp.) supplemented with 10% FBS and 0.01 mg/ml human recombinant insulin. The Py8119 cell line was maintained in F12K nutrient media (HyClone) supplemented with 5% FBS. Each cell line was cultured in standard medium as recommended by ATCC. All cells were incubated at 37°C in a humidified incubator containing 5% CO_2_. All cell lines are listed in the Supplemental Table 4.

### Chronic stress mouse model.

Stressed mice were restrained in a confined space that prevented them from moving freely or turning around but did not unduly compress them. This method induces chronic stress as evidenced by neuroendocrine activation and induction of both anxiety- and depression-like behaviors but does not cause pain or wounding (5). All mice were subjected to a pretreatment of stress for 7 days to acclimate these conditions for the study. Then, we inoculated MDA-MB-231 cells (1 × 10^6^) into the pretreated mice and randomly assigned them to control conditions or daily restraint stress for a maximum length of 30 days.

### Animal studies.

Four- to six-week-old NOD/SCID and C57BL/6 mice were used in each experimental group. MDA-MB-231 cells (1 × 10^6^ in PBS/Matrigel [1:1]) were injected s.c. into both flanks of NOD/SCID mice. E0771 and Py8119 tumor cells (5 × 10^6^ in PBS/Matrigel [1:1]) were injected into the fat pads of C57BL/6 mice. To perform serial dilution assays, 10^2^, 10^3^, 10^4^, and 10^5^ MDA-MB-231 tumor cells were injected s.c. into each dorsal flank. Tumor sizes were measured in perpendicular dimensions using calipers. Volumes were estimated using the formula (*a*^2^ × *b*)/2, where *a* is the shorter of the 2 dimensions and *b* is the longer. The *P* value was obtained by comparisons between the control and treatment groups at each time point. Detailed information on the mice is provided in Supplemental Table 5.

For the metastatic mouse model, 4- to 6-week-old female BALB/c mice were injected with MDA-MB-231 cells (5 × 10^5^ in 150 μl PBS) infected with an empty vector, shMYC, or shSLUG into the tail vein. Before being euthanized at 5 weeks following injection, all mice were subjected to a CT scan.

### Plasmid constructs and transfection.

Plasmids encoding human MYC (full-length, 1–215, 1–148, 1–95 aa) were generated by PCR amplification and subcloned into pcDNA6 expression vectors. Plasmids expressing the *SLUG* promoter (–2121–0, –1497–0, –997–0, –496–0, –200–0 bp) were generated by PCR amplification and subcloned into pGL3-basic expression vectors. Detailed information on the plasmid constructs is provided in Supplemental Table 8. Fidelity of all vectors was confirmed by DNA sequencing. Expression plasmids were transfected into cells using Lipofectamine 2000 (Invitrogen) according to the manufacturer’s instructions. OE-USP28 was from Lingqiang Zhang (Beijing Institute of Lifeomics, Beijing, China). USP28 shRNA expression plasmids and USP28 (C171A) and USP28 deletion constructs were gifts from Binhua P. Zhou (University of Kentucky, College of Medicine, Lexington, Kentucky, USA). LDHA shRNA expression plasmids and pEGFP-LDHA were provided by Qingkai Yang (Dalian Medical University, Dalian, China).

### Gene knockdown with shRNA.

Knockdown of genes was performed with specific shRNAs delivered using a lentiviral system purchased from Sigma-Aldrich Corp. according to the instructions provided by the manufacturer. In brief, to generate the lentivirus containing the specific shRNA, 293T cells were cotransfected with 2.5 mg pMD2.G and 7.5 mg psPAX2 compatible packaging plasmids and 10 mg of pLKO.1 plasmid bearing the specific shRNA for 24 hours. Culture medium containing the generated lentiviruses was collected and stored at –80°C as aliquots for further use. To deliver the specific shRNA construct, approximately 10% confluent cells were infected with lentiviruses bearing the specific shRNA in growth medium containing 8 mg/ml Polybrene and were incubated at 37°C for 24 hours. Transfected cells were subsequently selected with 2 mg/ml puromycin at approximately 50% confluence. Details on the shRNAs are provided in Supplemental Table 7.

### Quantitative reverse transcriptase PCR.

Total RNA was extracted using TRIzol reagent (Invitrogen) and used to generate cDNA by EasyScript One-Step gDNA Removal and cDNA Synthesis SuperMix (TransGen Biotech) with an oligo-dT primer. Real-time reverse transcriptase PCR was performed using SYBR Select Master Mix (Life Technologies) as recommended by the manufacturer. *ACTB* was used as the internal control. All primers are listed in Supplemental Table 6.

### Immunoprecipitation and immunoblot assays.

Cells were treated with the proteasome inhibitor MG132 (10 μM) for 6 hours before cell lysis. Coimmunoprecipitation was performed using 1 μg of antibodies and 200–500 μg of exogenous protein lysates. Protein A/G PLUS-Agarose immunoprecipitation reagent was then added, and this incubation was continued for 8 hours at 4°C. Beads were washed 3 times with 1 ml of coimmunoprecipitation buffer and subjected to Western blot analysis. Cells were lysed on ice in RIPA buffer, and protein concentrations were determined using Coomassie Brilliant Blue. Equal amounts of protein were subjected to electrophoresis on 10% gradient SDS-PAGE gels followed by immunoblot assays with the antibodies listed in Supplemental Table 1. See Supplemental data for the unedited blots.

### Luciferase reporter assay.

Cells were plated at a density of 1 × 10^5^ cells per well in 24-well plates. After 20 hours, cells were transfected with *SLUG* promoter–driven luciferase constructs (pGL3-*SLUG*) or control (pGL3-Basic) luciferase constructs. Fluc/Rluc activities were measured using the Dual Luciferase Reporter Assay System (Promega). Information on the Critical Commercial Assays is provided in Supplemental Table 3.

### Mammosphere culture.

Sphere formation was performed in ultralow attachment plates (Corning) with medium supplemented with 2% B27, 20 ng/ml bFGF, and 20 ng/ml EGF. MDA-MB-231 and MCF-7 cells were seeded at a density of around 2 cells/μl and cultured at 37°C with 5% CO_2_. After 14 days, spheres greater than 50 μm diameter were counted at ×40 magnification (Olympus). The reagents used are listed in Supplemental Table 2.

### Chromatin immunoprecipitation.

ChIP was performed using ChIP-IT Express Chromatin Immunoprecipitation Kits (Active Motif) according to the manufacturer’s protocol. See Supplemental Methods for details. Detailed information on the Critical Commercial Assays is provided Supplemental Table 3, and the primers used are listed in Supplemental Table 6.

### Immunohistochemical staining and statistical analysis.

Paraffin-embedded tissue blocks were used for immunohistochemical staining. Paraffin-embedded tissue specimens were sectioned, deparaffinized in xylene, and rehydrated, followed by antigen retrieval in sodium citrate, and the sections were then processed using SPlink Detection Kits (ZSGB-BIO) according to the manufacturer’s instructions. The sections were incubated with primary antibodies (1:200 dilution) overnight at 4°C. Specimens were stained using a DAB kit (ZSGB-BIO) until the desired stain intensity was developed. Sections were then counterstained with hematoxylin, dehydrated, and mounted. Staining intensity and extent of staining were graded as follows: negative (score 0), bordering (score 1), weak (score 2), moderate (score 3), and strong (score 4). Extent of staining was also grouped into quantiles according to the percentage of high-staining cells per field: negative (score 0), 25% (score 1), 26%–50% (score 2), 51%–75% (score 3), and 76%–100% (score 4). All immunohistochemical staining was evaluated and scored by at least 2 independent pathologists. Details on the Critical Commercial Assays are provided in Supplemental Table 3.

### Pharmacological studies.

Cells were maintained in DMEM for 8–12 hours that was followed by DMEM supplemented with 2% FBS for 5 days with different pharmaceutical treatments. The concentration of all drugs was chosen based on successful activation/inhibition in previous publications: epinephrine (10 nM), propranolol (10 μM), and ICI118,551 (10 μM). For other drugs, cells were maintained in 10% DMEM: vitamin C (1 μM), actinomycin D (5 μg/ml), cycloheximide (200 μg/ml). For mice, epinephrine (2 mg/kg/d, s.c.), propranolol (2 mg/kg/d, i.p.), and ICI118,551 (25 μM/100 μl, i.p.) were injected 7 days before tumor cell injection. The control group of mice received an equal volume of PBS. Detailed information on the chemicals used are listed in Supplemental Table 2.

### Metabolic assays.

Glucose uptake, lactate production, and ATP concentrations were measured by assay kits from BioVision. All procedures were performed as recommended by the manufacturer. Information on the Critical Commercial Assays is provided in Supplemental Table 3.

### Metabolomics analysis.

Cells were washed with 10 ml mannitol 3 times. This was followed by addition of 1 ml methanol containing 10 μM of d-camphor-10-sulfonic acid sodium salt as internal standards to each plate. Cells were then scraped (Corning) and transferred to a 5-ml Eppendorf tube. One milliliter of chloroform was then added to the tube, followed by vortexing for 30 seconds. Water in the amount of 400 μl was subsequently added to form a 2-phase system. After vortexing for 1 minute, the mixture was left to stand for 5 minutes and then centrifuged at 15,000 *g* for 15 minutes at 4°C. Subsequently, 450 μl of the upper layer was transferred and was filtered via centrifugation through a 5-kDa-cutoff filter (Millipore) to remove proteins (9100 *g*, 3 hours at 4°C). The filtrate was lyophilized and stored at –80°C. Before CE-TOF/MS analysis, dried samples were reconstituted in Milli-Q water (MilliporeSigma) containing 50 μM of trimesic acid and 2-naphtol-3,6-disulfonic acid disodium salt as internal standards to adjust for migration time in the anion mode.

### Screening of US drug collection of compounds against LDHA.

MDA-MB-231 cells were stably infected with LDHA that was subcloned into the pEGFP-C1 vector and then plated onto 96-well plates. Individual drugs were added to each well for 6 (1 μM, T1; or 2 μM, T2) and 12 hours (1 μM, T3) at 50% confluence. After washing of cells with PBS, LDHA expression in infected cells was determined by fluorescence detection.

### In vitro limiting dilution assay.

Limiting dilution assay was performed as described previously (70). Briefly, dissociated primary cells were seeded in 96-well plates at densities of 1, 2, 4, 8, 16, 32, and 64 cells per well. Wells with no sphere were counted for each group after 7 days.

### Statistics.

Each in vivo and in vitro experiment was performed in triplicate and repeated at least 3 times. Statistical analyses were performed with SPSS software (version 16.0) or GraphPad Prism 6.0 (GraphPad Software Inc.). Differences between variables were assessed by 2-tailed Student’s *t* test, 1-way ANOVA, and χ^2^ test, where appropriate. Data were expressed as mean ± SEM. *P* values less than 0.05 were considered statistically significant (*P < 0.05, **P < 0.01, ***P < 0.001).

### Accession number.

The mRNA array data were deposited in the GEO database with accession number GSE116781.

### Study approval.

All animals were housed and handled in accordance with the Animal Care and Use Committee at Dalian Medical University. All animal studies were conducted in accordance with the Dalian Medical University guidelines for animal care, and all animal procedures were approved by the IACUC of the Dalian Medical University (Dalian, China). All studies were performed in accordance with national animal protection laws (certificate number AEE18018). All studies involving patients’ samples were approved by the IRBs of the Cancer Center of Sun Yat-sen University, with informed consent.

## Author contributions

QL conceived the project and designed the experiments. BC, YL, PT, FP, JL, and YY designed and performed most of the experiments, whereas QS, JY, X. Luo, LY, WC, FA, BH, D. Liang, SW, PC, LS, X. Liu, HL, JX, and YP performed data analysis. YW, D. Li, PH, QY, LZ, BPZ, SL, and GX provided reagents. QL provided funds. QL, BC, FP, JL, YL, BL, EWFL, and KWK wrote the manuscript.

## Supplementary Material

Supplemental data

Supplemental Video 1

Supplemental Video 2

Supplemental Video 3

## Figures and Tables

**Table 6:**
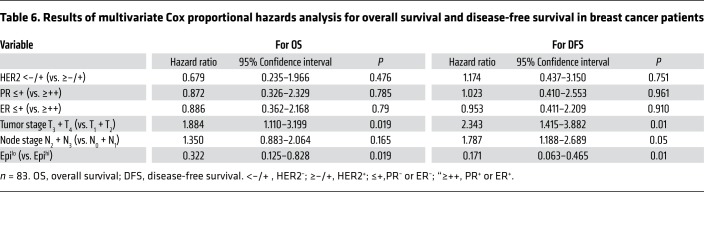
Results of multivariate Cox proportional hazards analysis for overall survival and disease-free survival in breast cancer patients

**Table 5:**
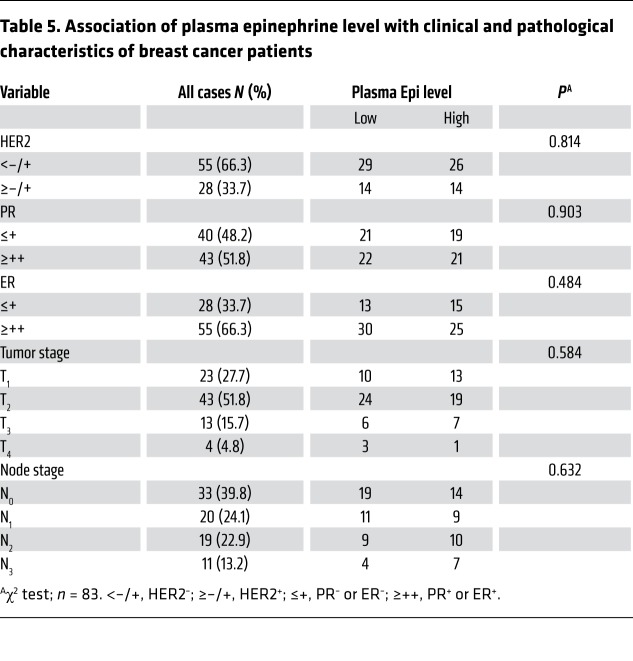
Association of plasma epinephrine level with clinical and pathological characteristics of breast cancer patients

**Table 4:**
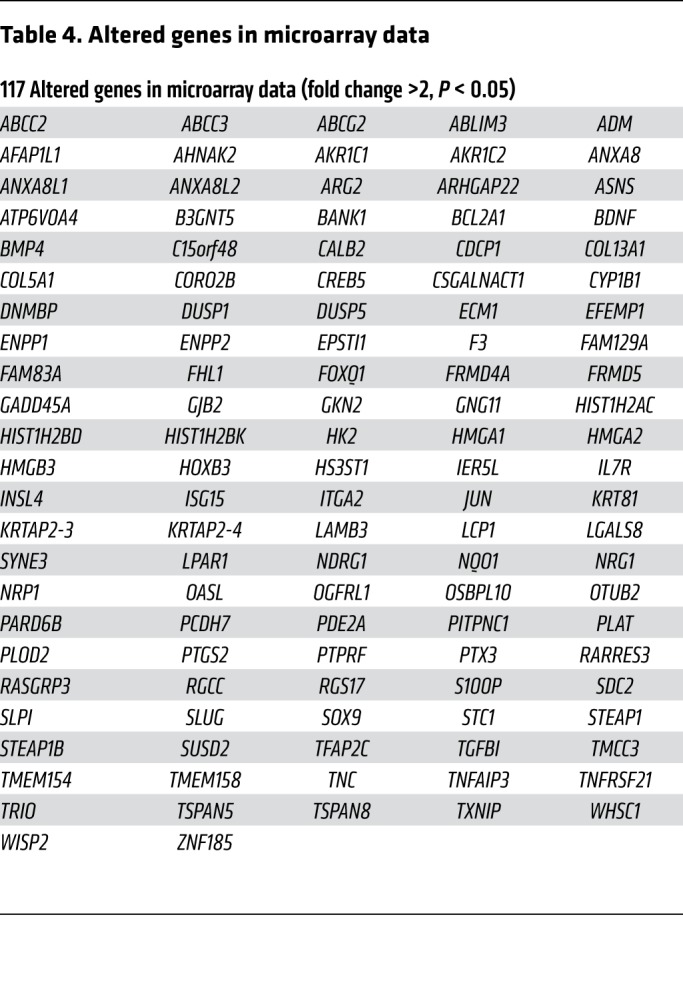
Altered genes in microarray data

**Table 1:**
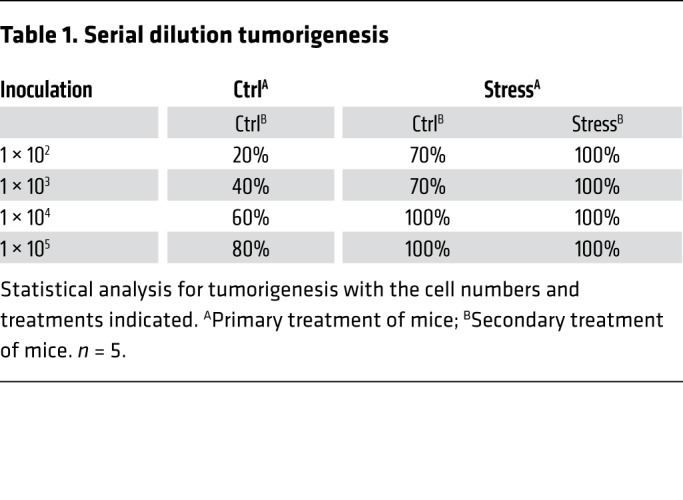
Serial dilution tumorigenesis

**Table 2:**
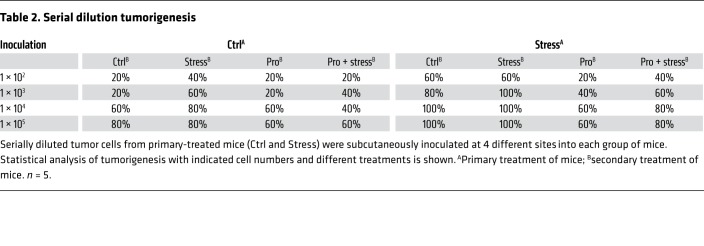
Serial dilution tumorigenesis

**Table 3:**
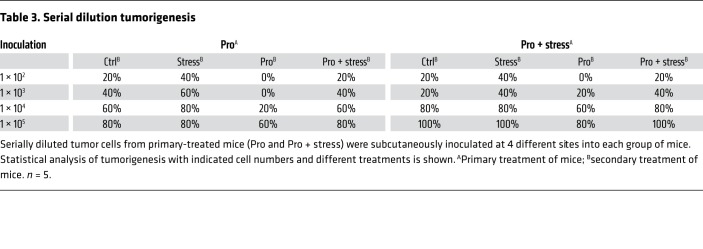
Serial dilution tumorigenesis

## Version 1 01/28/2019

Electronic publication

## Version 2 02/01/2019

Supplemental videos added.

## Version 3 03/01/2019

Print issue publication
