# Supplementary material for: Stress-induced epinephrine enhances lactate dehydrogenase A and promotes breast cancer stem-like cells
Source: J Clin Invest. 2019 Jan 28;129(3):1030–46. doi: 10.1172/JCI121685 (PMC6391112; doi:10.1172/JCI121685)

Supplemental Materials:

Supplemental Methods:

Wound healing assay. Cells were pre-treated with epinephrine for 5 days and then plated in six-well plates and grew to confluent cell monolayers. Subsequently, three horizontal scratches were made gently with sterile pipette tip across the diameter of the well and then incubated in serum-free medium. For each well, at least five pictures were taken microscopically at 0 h and 48 h after scratching. The percentage of wound healing was determined based on three measurements of the wound area.

Transwell invasion assay. Cells were pre-treated with epinephrine for 5 days. Then cells (2×10^4) resuspended in serum-free medium were placed in 50 μl matrigel (BD Biosciences) coated membrane in upper chamber (24-well insert, 8 μm, Corning Costar, China) and incubated for 36 h. Medium supplemented with 10% FBS were used as an attractant in the lower chamber. After being incubated for 36 h, cells invaded through the membrane were fixed with 4% paraformaldehyle (Santa Cruz) and stained with 0.5% crystal violet (Shanghai Sangon Company, China). The stained cell images were captured by microscope (Olympus, Japan), and five random fields at 10× magnification were counted.

Transwell migration assay. Cells were pre-treated with epinephrine for 5 days. Then cells (5×10^4) resuspended in serum-free medium were placed into uncoated membrane in the upper chamber (24-well insert, 8 μm, Corning Costar, China). Growth medium supplemented with 10% FBS was used as an attractant in the lower chamber. After being incubated for 24 h, cells migrated through the membrane were fixed with 4% paraformaldehyde (Santa Cruz, USA) and stained with 0.5% crystal violet (Shanghai Sangon Company, China). The stained cell images

were captured by microscope (Olympus, Japan), and five random fields at 10× magnification were counted.

Chromatin immunoprecipitation (ChIP). Cells (1×10^7) were fixed with 1% formaldehyde for 10 min at room temperature. Next, 10× glycine was added to the fixation solution to inhibit crosslinking. Cells were collected in PBS at 4°C, centrifuged at 1,000 ×*g* for 5 min, resuspended in 1 ml ice-cold 1% SDS buffer and lysed on ice for 30 min. The cells were homogenized on ice to aid release of nuclei. Cells were sonicated for 10 min at 7 watts average incident power (Covaris). Chromatin (25 μg) was immunoprecipitated for 12 h with 2 mg of specific antibodies directed against MYC (Abcam) and Protein G magnetic beads (25 ml). Beads were then washed sequentially for 5 min with the following buffers: once with ChIP Buffer I and twice with ChIP Buffer II. Immune complexes were eluted in 50 ml elution buffer AM2. Supernatants were reverse cross-linked by heating at 65°C for 12 h, treated with 1 ml RNaseA at 37°C for 15 min and digested with 2 ml proteinase K at 37°C for 1 h. DNA was obtained by phenol and phenol/chloroform extraction. The human *SLUG* promoter-specific primers used for PCR are listed in Supplemental Table F.

Extracellular Acidification Rate and Oxygen Consumption Rate Assays. The extracellular acidification rate (ECAR) and cellular oxygen consumption rate (OCR) were measured using the Seahorse XFe 24 Extracellular Flux Analyzer (Seahorse Bioscience). Experiments were performed according to the manufacturer's instructions. Briefly, cells were treated with epinephrine for 5 days and cell number was determined. Fifty thousand cells per well were then seeded into a Seahorse XF 24 cell culture microplate for 10 h, at which time cell number for each group was very similar. Cells were used for measurement of ECAR and OCR. Baseline measurements were collected. Then, for ECAR analysis, glucose, the oxidative phosphorylation

inhibitor oligomycin and the glycolytic inhibitor 2-DG were sequentially injected into each well at the indicated time points. For OCR, oligomycin, the reversible inhibitor of oxidative phosphorylation FCCP (p-trifluoromethoxy carbonyl cyanide phenylhydrazone) and the mitochondrial complex I inhibitor rotenone plus the mitochondrial complex III inhibitor antimycin A (Rote/AA) were sequentially injected. Data were analyzed using Seahorse XF-24 Wave software. OCR was reported in pmols/minute and ECAR in mpH/minute. The results were normalized to cell number.

Behavioral analyses. To assess the effects of chronic stress on locomotor and exploratory activities, mice were evaluated in an open field test. The open field was constructed of plywood and surrounded by walls 30 cm in height. The floor of the open field was 50 cm in length and 50 cm in width. Each mouse was placed individually at the center of the apparatus and was observed for 6 min to record locomotor activity (1). Total duration of immobility and latency induced by tail suspension were measured (2). Mice were suspended 45 cm above the floor by an adhesive tape placed approximately 1 cm from the tip of the tail. Time of immobility and latency was recorded during a 6-min period (3). All results were recorded and analyzed using Xeye Aba V3.2 software.

Molecular Dynamics Simulation. 1) USP28 and MYC modeling. The homology modeling program MODELLER 9.17 was used for characterizing USP28 and MYC motifs (AA: 46-74; confirmed by prior pull-down experiments (4, 5)) followed by MD optimization; 2) Protein Docking and MD simulation. We used the Zdock program to perform global rigid-body docking for $USP28^{WT}$ or $USP28^{C171A}$ complexed with the MYC^{46-74} motif. All the MD simulations were performed using Gromacs 4.6.7 with the Amber99sb force field; 3) Free energy surface (FES)

analysis. Free energy surfaces were obtained by integrating the deposited bias during metadynamics protocol implemented in the PLUMED program.

Prediction of Transcription Factor Binding Sites. Transcription factor binding sites were predicted using JASPAR 2014 software (http://jaspar.genereg.net/) (6). Since sensitivity and specificity are affected by the relative score threshold (default 80%), the submitted sequences were analyzed using a relative profile score threshold setting of 90% to the "CORE Vertebrata" database. This approach reports only the most likely sites (7) because experimentally reported binding sites in DNA frequently locate true sites as the highest-scoring sequences (8). Position frequency matrix cell numbers indicate the number of sequences with base x in column y. Sequence logos (9) are graphical representations of a transcription factor consensus binding site, in which nucleotides are sized and sorted relative to their occurrence at each position. The ranges are from 0 (no base preference) to 2 (single base occurrence).

Constant-pH molecular dynamics simulations (CpHMD). To delineate the effects of increased lactate product (the change of solvent pH), we employed constant-pH MD simulations (CpHMD) (10) to model the interactions of USP28 with MYC motif mimicking titration experiments at atomic level. The newest version of Amber16 and AmberTool17 (11) were used for CpHMD study with the AMBER99 force field and the GB implicit solvent model. In the present study, we used the distance of MYC^{Lys51} to $USP28^{C171\text{-}H600}$ to measure the binding capabilities of potential ubiquitin ligated sites in MYC motif with the catalytic domain of USP28. The pH 6.4 condition clearly enables a more stable interaction between MYC motif with the USP28 than that in neutral pH 7.4. Interestingly, in acidic pH 4.0 or basic pH 9.0 condition, the binding between MYC motif with USP28 appears unstable and fluctuates with increased frequency of dissociations.

Virtual screening of FDA-approved drugs against LDHA. Our in-house docking program FIPSDock63 was used to perform the virtual screening of 2037 FDA-approved small molecule drugs against LDHA. Noteworthy, vitamin C stands out in the vitamins group (seven hits) among top 200 hits in the virtual screening campaign.

MTT assay. MDA-MB-231 cells (2×10^3) were plated onto 96-well flat bottom plates in a final volume of 100 μl/well. After attached, cells were exposed to test compounds for times indicated and viability examined at 490 nm.

Microarray analysis. Total RNA was extracted by Trizol and submitted to the Gene Tech Company Limited (Shanghai, China) for labelling and hybridization for 16 h at 45°C using Affymetrix Clariom D. Microarray scans were obtained with a GeneChip Scanner 3000 7G (Affymetrix, Santa Clara, CA) using the default settings. Data were normalized with the Robust Multichip Analysis (RMA) algorithm using default analysis settings and some additional median/quantile normalization. We then eliminated all probes with a mean < 6.0 and standard deviation < 1.0 to filter the number of probes from 49,293 to 26,000. Then, we normalized the data with a fold change > 2 and P value < 0.05 (117 genes). We further selected the data with a fold change > 2 and Q value < 0.05 by the method with Benjamini-hochberg, resulting in 54 genes being chosen, and which were included in the 117 genes. Gene Ontology Enrichment analysis was performed in the website (https://david.ncifcrf.gov).

Gene set enrichment analysis (GSEA). We searched the publicly available databases to identify differential gene expression between cancer stem cells (CSCs) and non-CSCs with replicates. Four GEO datasets GSE65576 (bulk tumor cells and spheriod-cultured cell with stem cell medium from glioblastoma samples), GSE33874 (side population (SP) and main population (MP) of human ovarian adenocarcinoma samples), GSE36563 (SP and MP samples of

xenografts which derived from human pancreatic ductal adenocarcinoma samples) and GSE59281 ($CD44^{+}/CD24^{low}$ and $CD44^{+}/CD24^{-}$ breast cancer cell line) were subsequently selected and analyzed by GEO2R.

References

1. Rodrigues AL, et al. Involvement of monoaminergic system in the antidepressant-like effect of the hydroalcoholic extract of Siphocampylus verticillatus. Life Sci. 2002;70(12):1347-1358.
2. Steru L, Chermat R, Thierry B, Simon P. The tail suspension test: a new method for screening antidepressants in mice. Psychopharmacology (Berl). 1985;85(3):367-370.
3. Tomida S, et al. Usp46 is a quantitative trait gene regulating mouse immobile behavior in the tail suspension and forced swimming tests. Nat Genet. 2009;41(6):688-695.
4. Popov N, et al. The ubiquitin-specific protease USP28 is required for MYC stability. Nat Cell Biol. 2007;9(7):765-774.
5. Diefenbacher ME, et al. Usp28 counteracts Fbw7 in intestinal homeostasis and cancer. Cancer Res. 2015;75(7):1181-1186.
6. Mathelier A, et al. JASPAR 2014: an extensively expanded and updated open-access database of transcription factor binding profiles. Nucleic Acids Res. 2014;42(Database issue):D142-147.
7. van den Hoven R, Gur E, Schlamanig M, Hofer M, Onmaz AC, Steinborn R. Putative regulation mechanism for the MSTN gene by a CpG island generated by the SINE marker Ins227bp. BMC Vet Res. 2015;11:138.
8. Benitez-Bellon E, Moreno-Hagelsieb G, Collado-Vides J. Evaluation of thresholds for the detection of binding sites for regulatory proteins in Escherichia coli K12 DNA. Genome Biol. 2002;3(3):RESEARCH0013.
9. Schneider TD, Stephens RM. Sequence logos: a new way to display consensus sequences. Nucleic Acids Res. 1990;18(20):6097-6100.

10. Mongan J, Case DA, McCammon JA. Constant pH molecular dynamics in generalized Born implicit solvent. J Comput Chem. 2004;25(16):2038-2048.
11. Case DA, et al. The Amber biomolecular simulation programs. J Comput Chem. 2005;26(16):1668-1688.

Supplemental Table 1:

**Antibodies**		
**REAGENT or RESOURCE**	**SOURCE**	**Catalog**
β–Catenin (Rabbit monoclonal)	Cell Signaling Technology	Cat#8480
NANOG (Rabbit polyclonal)	Abcam	Cat#ab80892
OCT-4 (Mouse monoclonal)	Cell Signaling Technology	Cat#75463
ADRB1 (Rabbit polyclonal)	Abcam	Cat#ab3442
ADRB2 (Rabbit monoclonal)	Abcam	Cat#ab182136
SLUG (Rabbit polyclonal)	Abcam	Cat#27568
MYC (Rabbit or Mouse monoclonal)	Cell Signaling Technology or Abcam (ChIP)	Cat#5605S or Cat#ab32
USP28 (Rabbit polyclonal)	Proteintech	Cat#17707-1-AP
LDHA (Rabbit monoclonal)	Cell Signaling Technology	Cat#3582S
PKM2 (Rabbit monoclonal)	Cell Signaling Technology	Cat#4053S
PDK1 (PDHK1) (Rabbit monoclonal)	Cell Signaling Technology	Cat#3820S
PFKM (Rabbit polyclonal)	Proteintech	Cat#55028-1-AP
HK2 (Rabbit monoclonal)	Cell Signaling Technology	Cat#2106S
HA (Rabbit monoclonal)	Proteintech	Cat#51064-2-AP
TWIST1 (Rabbit polyclonal)	Proteintech	Cat#25465-1-AP
SNAIL(Rabbit polyclonal)	Abcam	Cat# ab180714
Ubiquitin (Mouse monoclonal)	Cell Signaling Technology	Cat#3936
GAPDH (Mouse monoclonal)	Proteintech	Cat#60004-1-Ig
Goat anti-Mouse IgG Secondary Antibody	Thermo Fisher Scientific	Cat#31430
Goat anti-Rabbit IgG Secondary Antibody	Thermo Fisher Scientific	Cat#31460

Supplemental Table 2:

Chemicals, Peptides and Recombinant Proteins		
**REAGENT or RESOURCE**	**SOURCE**	**Catalog**
MG132	Selleck	Cat#S2619;
Protein A/G PLUS-Agarose Immunoprecipitation Reagent	Santa Cruz	Cat#sc2003
Cycloheximide	Coolaber	Cat#CC4071;
Lipofectamine 2000	Invitrogen	Cat#11668-019
Puromycin Dihydrochloride	ThermoFisher	Cat#A1113803;
Atenolol	Sigma	Cat#A7655-1G;
ICI118,551 hydrochloride	Sigma	Cat#I127;
TRIzol reagent	Life Technologies	Cat#15596-026
Epinephrine bitartrate	Selleck	Cat#S2521
Propranolol hydrochloride	Sigma	Cat#P0884;
Norepinepphrine	Isoreag	Cat#IR-15054S
Cortisol	Sigma	Cat#C-113
Actinomycin D	Abmole	Cat#M4881;
SYBR Select Master Mix	Life Technologies	Cat#4472908
EasyScript One-Step gDNA Removal cDNA Synthesis SuperMix	Transgen	Cat#AE311-03
B27	Life Technologies	Cat#17504-044
bFGF	PEPROTECH	Cat#100-18B
EGF (hEGF)	Sigma	Cat#E9644;
Sodium Oxamate	Sigma	Cat#O2751;
L-(+)-Lactic acid	Sigma	Cat#L1750;
L-Ascorbic acid	Sigma	Cat#V900134;

Supplemental Table 3:

**Critical Commercial Assays**		
**REAGENT or RESOURCE**	**SOURCE**	**Catalog**
WesternBright™ECL kit	Advansta	K-12045-D50
DAB kit	ZSGB-BIO	ZLI-9018
Dual-Luciferase® Reporter Assay System	Promega	E1910
ChIP-IT Express Chromatin Immunoprecipitation Kits	Active Motif	53008
SPlink Detection Kits	ZSGB-BIO	SP-9000
Glucose uptake	BioVision	K606-100
Lactate production	BioVision	K627-100
ATP levels were measured by assay kits	BioVision	K354-100
Cell Mito Stress Test Kit	Agilent	103015-100
Glycolysis Stress Test Kit	Agilent	103020-100

Supplemental Table 4:

**Experimental Models: Cell Lines**		
**REAGENT or RESOURCE**	**SOURCE**	**Catalog**
MDA-MB-231	ATCC	Cat# CRM-HTB-26
MCF-7	ATCC	Cat# HTB-22™
293T	ATCC	Cat#CRL-3216™
Py8119	Fudan University Shanghai	
E0771	BeNa Culture Collection	BNCC342034

Supplemental Table 5:

**Experimental Models: Organisms/Strains**		
NOD/SCID mouse model	Female	Beijing Vital River Laboratory Animal Technology Co., Ltd.
BALB/C mouse model	Female	Beijing Vital River Laboratory Animal Technology Co., Ltd.
C57BL/6 mouse model	Female	Dalian Medical University

Supplemental Table 6:

**Oligonucleotides**		
**RT-q-PCR (Sangon Biotech)**	**sense (5′-3′)**	**antisense (5′-3′)**
MYC	TCAAGAGGCGAACACACAAC	GGCCTTTTCATTGTTTTCCA
SLUG	ATGAGGAATCTGGCTGCTGT	CAGGAGAAAATGCCTTTGGA
POU5F1	GAGAACCGAGTGAGAGGCAACC	CATAGTCGCTGCTTGATCGCTTG
CTNNB1	ATGGAGCCGGACAGAAAAGC	TGGGAGGTGTCAACATCTTCTT
NANOG	CCAAATTCTCCTGCCAGTGAC	CACACGTCTTCAGGTTGCAT
ABCG2	ATCAGCTGGTTATCACTGTGAGGCC	AGTGGCTTATCCTGCTTGGAAGGC
TFAP2C	GAAGAGGACTGCGAGGATCG	GCTGATATTCGGCGACTCCA
BMP4	GGAGGAGGAGGAAGAGCAGA	CACTGGTCCCTGGGATGTTC
WISP2	CTGGCCTTGTCTCTTCCCTG	AGAAGCGGTTCTGGTTGGAC
NDRG1	AGGAGCAGGACATCGAGACT	CGATGTCATGGTAGGTGAGG
HS3ST1	ATGCACACATGCTGAACTGG	GCAGTAGAAGCCCTTGGTTTTG
TMCC3	AAGAGCCGGGTAGAACGTCAT	TCAAAGTTGAGGTTGGTGTCTG
LDHA	CACCAAAGATTGTCTCTGGCA	AAGATGTTCACGTTACGCTGG
USP28	GGACCCTTCCTTTCTCCATGA	AGGCTGACTGCCTGAGTAATGTC
ACTB	TTGCCGACAGGATGCAGAAGGA	AGGTGGACAGCGAGGCCAGGAT
**PCR (ChIP) (Sangon Biotech)**	**sense (5′-3′)**	
SNAI2-pro0-R	CTTGCCAGCGGGTCTGGCGG	
SNAI2-pro394-F	CTCACCGAGCGAGGTTACCT	
SNAI2-pro394-R	CCCCGCCCGCGATCCACGCTC	
SNAI2-pro496-F	GCGCGCTGGCGCTGCACCACA	

Supplemental Table 7:

**Oligonucleotides**		
**siRNAs (GenePharma, Suzhou, China)**	**sense（5′-3′）**	
siNC	5′-UUCUCCGAACGUGUCACGU-3′	
siUSP28-1	5′-ACUCAGACUAUUGAACAGAUGUACUGC-3′	
siUSP28-2	5′-CUGCAUGCAAGCGAUAAGG-3′	
siUSP28-3	5′-CUGCAUUCACCUUAUCAUU-3′	
siADRB2-1	5′-GCCAUUACUUCACCUUUCA-3′	
siADRB2-2	5′-GCCUAGCGAUAACAUUGAU-3′	
siADRB2-3	5′-CGCCCAUAUUCUUAUGAAA-3′	
siADRB2-4	5′-CAGAGUGGAUAUCACGUGGAA-3′	
siADRB1-1	5′-CCGCUGUCUCAGCAGUGGA-3′	
siADRB1-2	5′-CGCUCACCAACCUCUUCAU-3′	
siADRB1-3	5′-CCUCGUCCGUAGUCUCCUU-3′	
siADRB1-4	5′-CCGAUAGCAGGUGAACUCGAA-3′	
siLDHA	5′-GGCAAAGACUAUAAUGUAA-3′	
siHK2	5′-CCTGGGTGAGATTGTCCGTAA-3′	
**shRNA**	**sense（5′-3′）**	**antisense (5′-3′)**
shSLUG-1	CCGGCCCATTCTGATGTAAAGAAATCTCGAGATTTCTTTACATCAGAATGGGTTTTT	AATTAAAAACCCATTCTGATGTAAAGAAATCTCGAGATTTCTTTACATCAGAATGGG
shSLUG-2	CCGGCCGAAGCCAAATGACAAATAACTCGAGTTATTTGTCATTTGGCTTCGGTTTTT	AATTAAAAACCGAAGCCAAATGACAAATAACTCGAGTTATTTGTCATTTGGCTTCGG
shSLUG-3	CCGGGAGTGACGCAATCAATGTTTACTCGAGTAAACATTGATTGCGTCACTCTTTTT	AATTAAAAAGAGTGACGCAATCAATGTTTACTCGAGTAAACATTGATTGCGTCACTC
shMYC-1	CCGGCCCAAGGTAGTTATCCTTAAACTCGAGTTTAAGGATAACTACCTTGGGTTTTT	AATTAAAAACCCAAGGTAGTTATCCTTAAACTCGAGTTTAAGGATAACTACCTTGGG
shMYC-2	CCGGACTGAAAGATTTAGCCATAATCTCGAGATTATGGCTAAATCTTTCAGTTTTTT	AATTAAAAAACTGAAAGATTTAGCCATAATCTCGAGATTATGGCTAAATCTTTCAGT

Supplemental Table 8:

Oligonucleotides		
**Plasmid**	**forward primer (5'-3')**	**reverse primer (5'-3')**
plvx-SNAI2	CCGGAATTCGCCACCATGCCGCGCTCCTTCCTGGT	CGCGGATCCTCAGTGTGCTACACAGCAGC
plvx-SNAI2-Red	CCGGAATTCGCCACCATGCCGCGCTCCTTCCTGGT	CGGGATCCCGGTGTGCTACACAGCAGCCAG
PCS2-SLUG	CAGGATCCGCCACCATGCCGCGCTCCTTCCTGGT	TTGAATTCTCAGTGTGCTACACAGCAGCCAG
pcDNA6-His-SLUG	CAGGATCCGCCACCATGCCGCGCTCCTTCCTGGT	CAGAATTCCGGTGTGCTACACAGCAGCCAG
**Luciferase constructs**	**forward sense (5'-3')**	**reverse sense (5'-3')**
pGL3-basic-SNAI2 1k	CGGGGTACCAAAGATAAGATCTCTTGTC	CCGCTCGAGCTTGCCAGCGGGTCTGGCG
pGL3-basic-SNAI2 1.5k	CGGGGTACCGACAATGCACTTTTCTCTG	CCGCTCGAGCTTGCCAGCGGGTCTGGCG
pGL3-basic-SNAI2 2k	CGGGGTACCTGGATTATGCCTCTGTGATCC	CCGCTCGAGCTTGCCAGCGGGTCTGGCG
pGL3-basic-SNAI2 Mut1	TAGGGACCGCAAAATCCTCCCGCC	GGCGGGAGGAAAAAGCGGTCCCTA
pGL3-basic-SNAI2 Mut2	CGCACCTGAGAAAAGCCCCTGCCC	GGGCAGGGGCAAAACTCAGGTGCG
pGL3-basic-SNAI2 Mut3	TCCCAGAGAGAAAAAATCGCGGGCG	CGCCCGCGATAAAAACTCTCTGGGA
pGL3-basic-POU5F1 2k	CGGGGTACCCCCTGGCCCAGAGCCCCCTC	CATGCCATGGGGAAGGAAGGCGCCCCAAGC

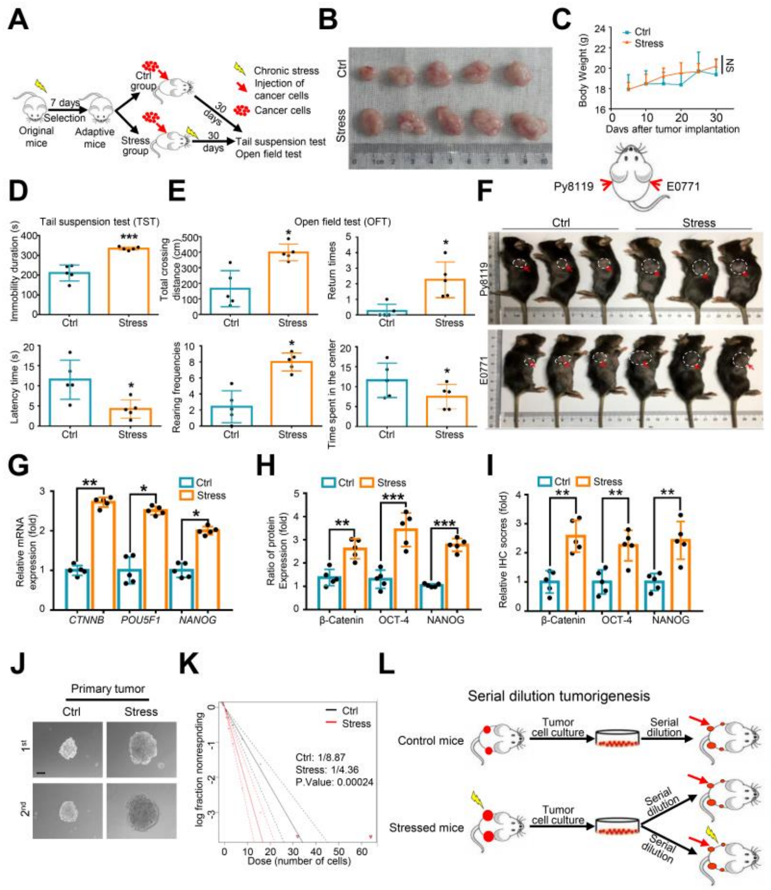

Supplemental Figure 1. Chronic stress promotes CSC-like properties in mouse model. (A) Schematic diagram of the chronic stress mouse model. The yellow flash indicates chronic stress, the red arrow indicates injection of cancer cells and the red dots indicate cancer cells. (B) Representative tumor image of MDA-MB-231 tumors in Control (Ctrl) and Stressed mice; n=5. (C) Body weight growth curves of mice over 30 days following tumor implantation. n=5 (1-way ANOVA). (D) Mice were subjected to behavioral tests after completion of the chronic stress paradigm. Immobility duration (upper panel) and latency time (lower panel) were analyzed by the tail suspension test. n=5 (1-way ANOVA). (E) Total crossing distance (upper-left panel), return times (upper-right panel), rearing frequencies (lower-left panel) and time spent in the center (lower-right panel) were analyzed with an open field test. n=5 (1-way ANOVA). (F) Diagram of Py8119 and E0771 cells injected sites (upper panel) and representative tumor images from control (Ctrl) and stressed (Stress) mice injected with these tumor cell lines (bottom panel), n= 6. (G and H) Relative mRNA levels (G) and protein levels (H) of selected proteins in tumor cells from Ctrl- or stress-induced mice. Intensity of protein expression was quantified by densitometry and differences expressed as fold changes. n=5 (Student's *t* test). (I) IHC scores of indicated proteins from Ctrl- and stress-induced tumor tissue. n=5 (Student's *t* test). (J) Representative images of primary and secondary sphere formation of primary MDA-MB-231 tumors from the Ctrl and Stress groups. Scale bar, 50 μm (K) Limiting dilution assay of Ctrl and stressed MDA-MB-231 xenografted tumor cells. n=3 (χ^2 test). (L) Serially diluted tumor cells were subcutaneously inoculated at 4 different sites into each mouse. Data are representative of 3 independent experiments. Data represent mean ± SEM, *p < 0.05, **p < 0.01, ***p < 0.001.

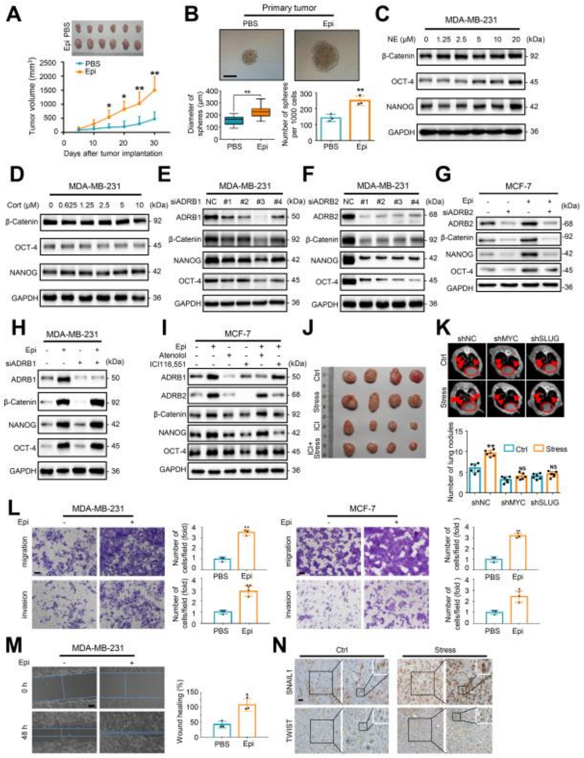

Supplemental Figure 2. Epinephrine promotes CSC-like traits via ADRB2. (A) Representative tumor image derived from PBS- and Epi-treated mice (upper panel). Scale bar, 1 cm (1-way ANOVA). Tumor growth curves are shown (lower panel). n=6. (B) Representative spheroid images derived from PBS- and epinephrine (Epi)-induced tumors cells (upper panel). Scale bar, 100 μm. Bottom left panel shows distribution pattern of mammosphere size from tumor cells. Bottom right panel shows the number of mammospheres (d > 50 μm); n=3 (1-way ANOVA). (C and D) Immunoblot analysis with indicated antibodies in MDA-MB-231 cells treated with increasing concentrations of NE (C) and Cort (D). (E and F) MDA-MB-231 cells were transfected with 4 different siRNAs targeting ADRB1 (E) or targeting ADRB2 (F). Cell lysates were analyzed for the expression of indicated proteins. (G) MCF-7 cells were transfected with siADRB2 and then treated with Epi for 5 days. Expression of proteins was determined by immunoblot analysis. (H) MDA-MB-231 cells were transfected with siRNA targeting ADRB1 with or without Epi for 5 days. Cell lysates were analyzed for the expression of indicated proteins. (I) MCF-7 cells were treated with PBS, atenolol (A), or ICI118,551 (ICI) in the presence or absence of Epi for 5 days. Immunoblot analysis was used to analyze the expression of indicated proteins in the cell lysates. (J) Representative tumor image derived from Ctrl or stressed mice in the presence or absence of ICI. n=5. (K) Representative images of CT scan of mice with indicated treatments. Statistical significance was determined by one-way ANOVA test (n=6). (L) Representative images of MDA-MB-231 and MCF-7 cells in transwell assays. Statistical significance was determined by one-way ANOVA test. n=3, Scale bar, 100 μm. (M) Representative images of MDA-MB-231 cells in wound healing assay. Statistical significance was determined by one-way ANOVA test. n=3. Scale bar, 100 μm. (N) Representative IHC staining for indicated antibodies of Ctrl and stressed tumor tissue. n=5, Scale bar, 50 μm. Data are representative of at least 3 independent experiments. Data represent mean ± SEM, $*p < 0.05$, $**p < 0.01$.

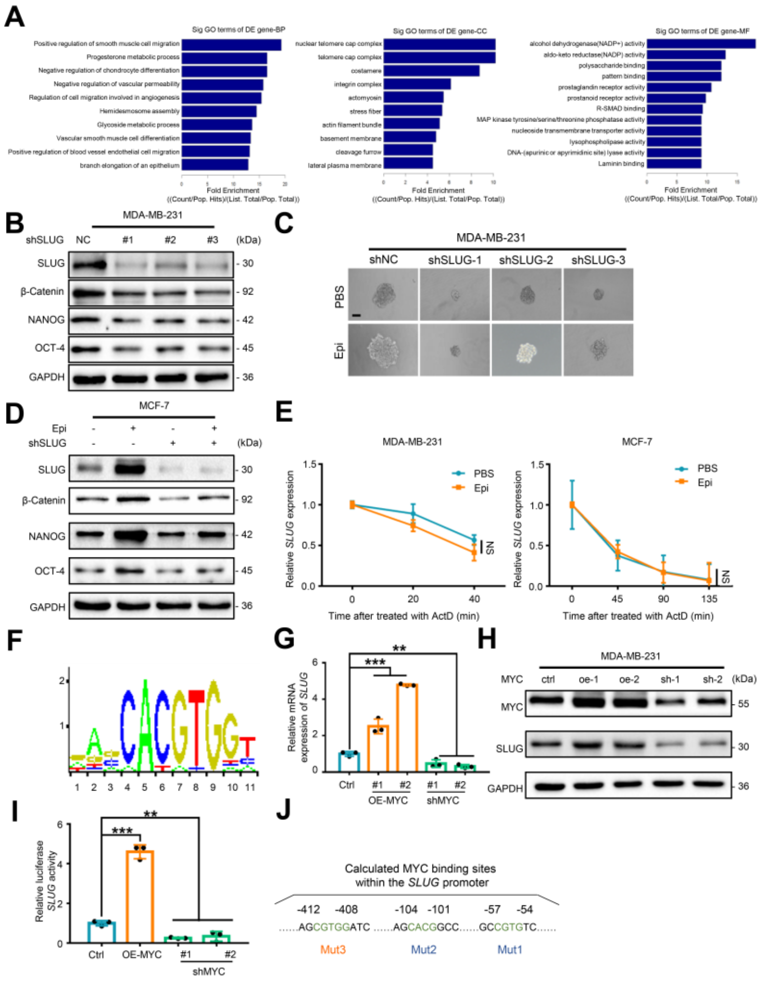

Supplemental Figure 3. Epinephrine transactivates SLUG via MYC. (A) Gene set enrichment analysis of 117 altered genes (fold change > 2 and P value < 0.05) showing the top 10 most-enriched gene sets. (B) Immunoblot analysis of MDA-MB-231 cells transfected with 3 different shRNAs targeting SLUG. (C) Representative images of mammospheres from shNC or shSLUGs MDA-MB-231 cells and treated with PBS or Epi for 5 days. n=3. Scale bar, 50 μm. (D) MCF-7 cells were transfected with shRNA-1 targeting SLUG in the presence or absence of Epi for 5 days. Cell lysates were subjected to immunoblot analysis for selected proteins. (E) MDA-MB-231 and MCF-7 cells were treated with Epi for 5 days. Actinomycin D (ActD) was added for the indicated times before cells were harvested. Half-life of mRNAs for each treatment was predicted as described. n=3 (1-way ANOVA). (F) Prediction of transcription factor binding site in the SLUG promoter using the JASPAR database. (G and H) MDA-MB-231 cells were transfected with MYC overexpression (oe-1, oe-2) or knockdown (sh-1, sh-2) vectors, mRNA levels were verified by RT-qPCR (G). n=3 (1-way ANOVA). Cell lysates were subjected to immunoblot analysis with the indicated antibodies (H). (I) MDA-MB-231 cells were transfected with ctrl or oe-MYC or MYC shRNA for 48 h, and then transfected with a pGL3-SLUG (-496~0) truncated promoter. After 24 h, cells were harvested for dual-luciferase analysis. n=3 (1-way ANOVA). (J) Schematic diagram of SLUG WT, mutant 1 (Mut1, -57 to -54), mutant 2 (Mut2, -104 to -101), or mutant 3 (Mut3, -412 to -408). Data are representative of at least 3 independent experiments. Data represent mean ± SEM, $^{**}p < 0.01$, $^{***}p < 0.001$.

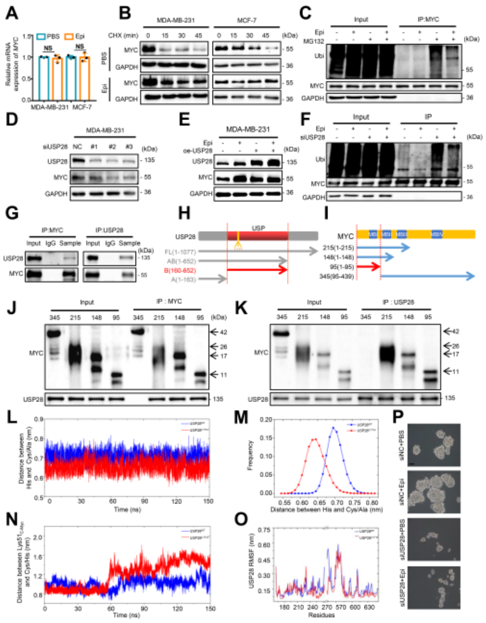

Supplemental Figure 4. Epinephrine stabilizes MYC via USP28 deubiquitination. (A) MDA-MB-231 and MCF-7 cells were treated with PBS or Epi for 5 days. Expression of MYC mRNA was verified by RT-qPCR. n=3 (Student's *t* test). (B) Immunoblots of cancer cells treated with Epi for 5 days followed by treated with cycloheximide (CHX) for the indicated times. (C) Ubiquitin assays of 293T cells transfected with ubiquitin (UB) and MYC followed by treated with Epi and/or MG132. (D) Immunoblot analysis of MDA-MB-231 cells transfected with 3 different shRNAs targeting USP28. **(**E) MDA-MB-231 cells were transfected with oe-USP28 and then treated with PBS or Epi for 5 days. Expression of the indicated proteins was examined by immunoblotting. (F) MYC and HA-UB were co-expressed with siRNA-2 targeting USP28 in the presence or absence of Epi in 293T cells. MYC was immunoprecipitated and the polyubiquitination of MYC was detected by immunoblotting. (G) MYC and USP28 were transfected into 293T cells. MYC and USP28 were immunoprecipitated with a MYC or USP28 antibody, respectively, and USP28 and MYC were analyzed by immunoblotting. (H) Schematic diagram of USP28 structure and deletion constructs. (I) Schematic diagram showing the structure of MYC and deletion constructs that were used. (J and K) Deletion mutants of MYC were co-expressed with USP28 in 293T cells. Extracts were immunoprecipitated with MYC (J) or USP28 (K) antibody Bound USP28 or MYC was then examined by immunoblotting. (L) Distance between His^{600} and the Cys/Ala mutation site in USP28 during a 150-ns MD simulation. The USP28^{WT} profile is displayed in blue and the USP28^{C171A} profile is depicted in red. (M) Frequency distribution histogram for the distance between His^{600} and Cys/Ala mutation site in USP28 during a 150-ns MD simulation. The USP28^{WT} profile is displayed in blue and the USP28^{C171A} profile is shown in red. (N) Distance between the center of mass of MYC^{Lys51} and center of mass of USP28$^{His\text{-}Cys/Ala}$ during a 150-ns MD simulation. The USP28^{WT} profile is shown in blue and the USP28^{C171A} profile is shown in red. (O) RMSF profiles for USP28^{WT} and USP28^{C171A} during a 150-ns MD simulation. (P) Representative spheroid images formed by cells transfected with USP28 siRNA-2 followed by treated with PBS or Epi for 5 days. Data are representative of at least 3 independent experiments. Data represent mean ± SEM.

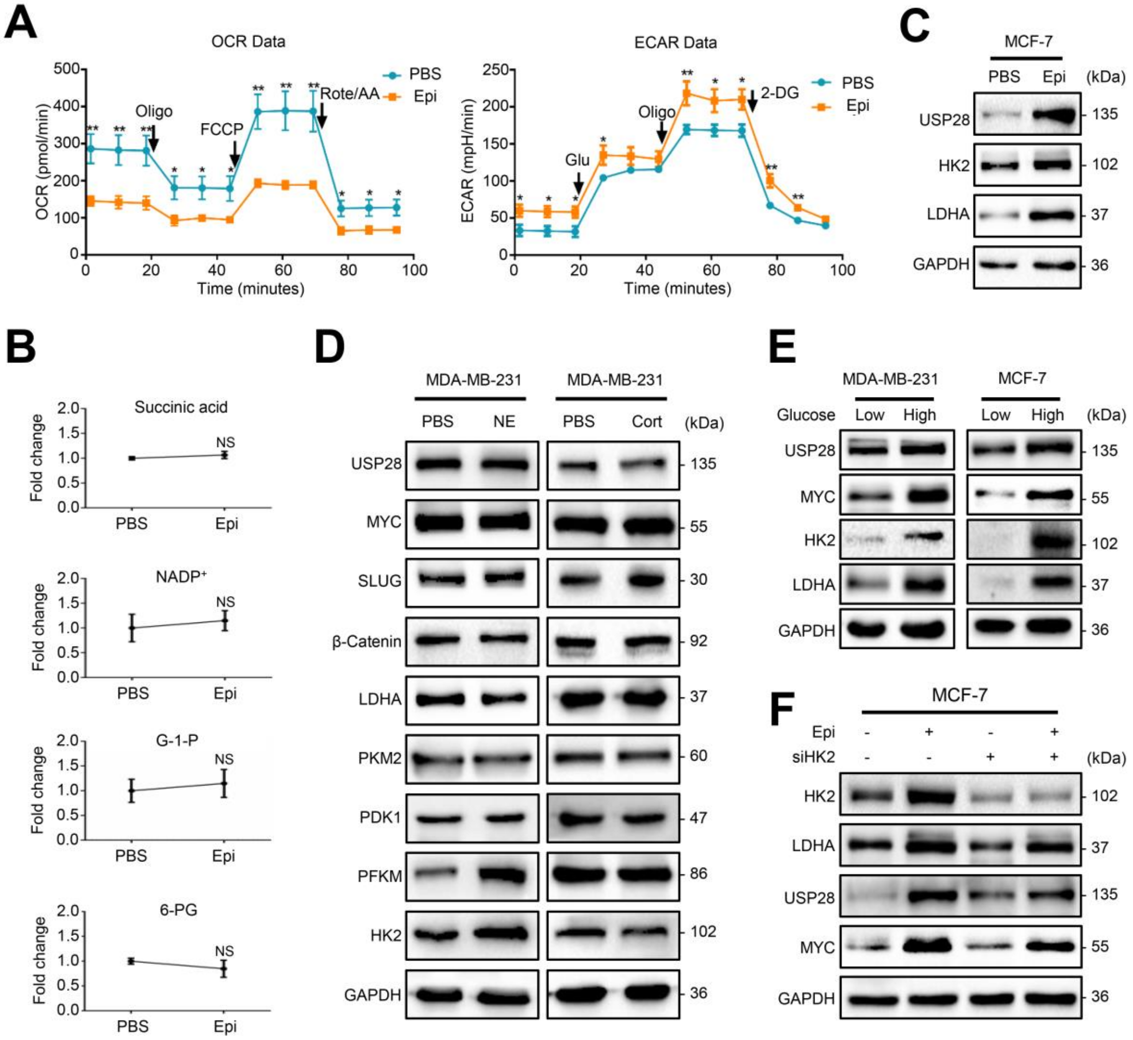

Supplemental Figure 5. Epinephrine enhances USP28 signaling through LDHA. (A) MDA-MB-231 cells were treated with epinephrine for 5 days and OCR and ECAR were then measured. n=3 (1-way ANOVA). (B)Average fold change of glycolytic metabolites was measured by capillary electrophoresis-mass spectrometry. G-1-P, glucose-1-phosphate; 6-PG, 6-phosphogluconic acid. n=3 (Student's t test). (C) MCF-7 cells were treated with PBS or Epi for 5 days and cell lysates were subjected to immunoblot analysis to quantify selected proteins. (D) Immunoblot analysis with indicated antibodies in MDA-MB-231 cells treated with NE or Cort (10 μM). (E) MDA-MB-231 and MCF-7 cells were maintained in low- (1 g/l) or high-glucose (4.5 g/l) and cell lysates were subjected to immunoblot analysis to detect the indicated proteins. (F) MCF-7 cells were transfected with HK2 siRNA in the presence or absence of Epi for 5 days. Cell lysates were immunoblotted with antibodies against the indicated proteins. Data are representative of at least 3 independent experiments. Data represent mean ± SEM, *p < 0.05, **p < 0.01.

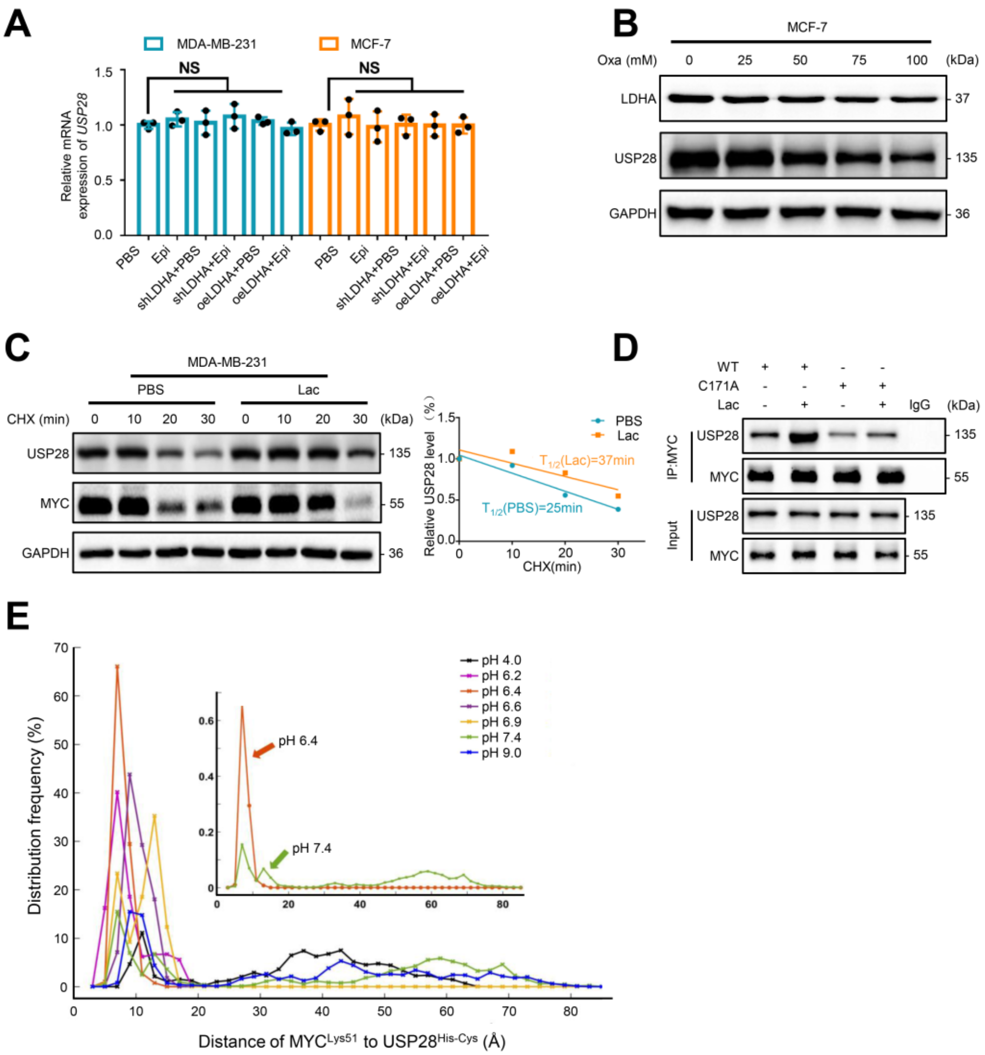

Supplemental Figure 6. Epinephrine stabilizes USP28 through LDHA. (A) MDA-MB-231 and MCF-7 cells were transfected with shLDHA or oeLDHA in the presence or absence of Epi for 5 days. USP28 mRNA were examined by RT-qPCR. n=3 (1-way ANOVA). (B) MCF-7 cells were treated with the indicated concentrations of sodium oxamate (Oxa) for 48h and expression of the indicated proteins was examined by immunoblotting. (C) MDA-MB-231 cells were treated with lactate (Lac) for 72 h. After treating the cells with CHX for the indicated times, expression of USP28 was analyzed by immunoblotting (left panel). Intensity of USP28 expression for each time point was quantified by densitometry and plotted against time (right panel). Half-life ($T_{1/2}$, min) of USP28 following PBS was 25 min and for Epi it was 37 min in MDA-MB-231 cells. (D) 293T cells were transfected with USP28 WT or C171A and then treated with lactate for 72 h. Extracts were immunoprecipitated with a MYC antibody and probed for expression of USP28 and MYC. (E) The distribution frequency for the distance of MYC^{Lys51} to $USP28^{C171\text{-}H600}$ in different pH conditions computed by constant pH molecular dynamics (CpHMD) simulation method. Data are representative of at least 3 independent experiments. Data represent mean ± SEM.

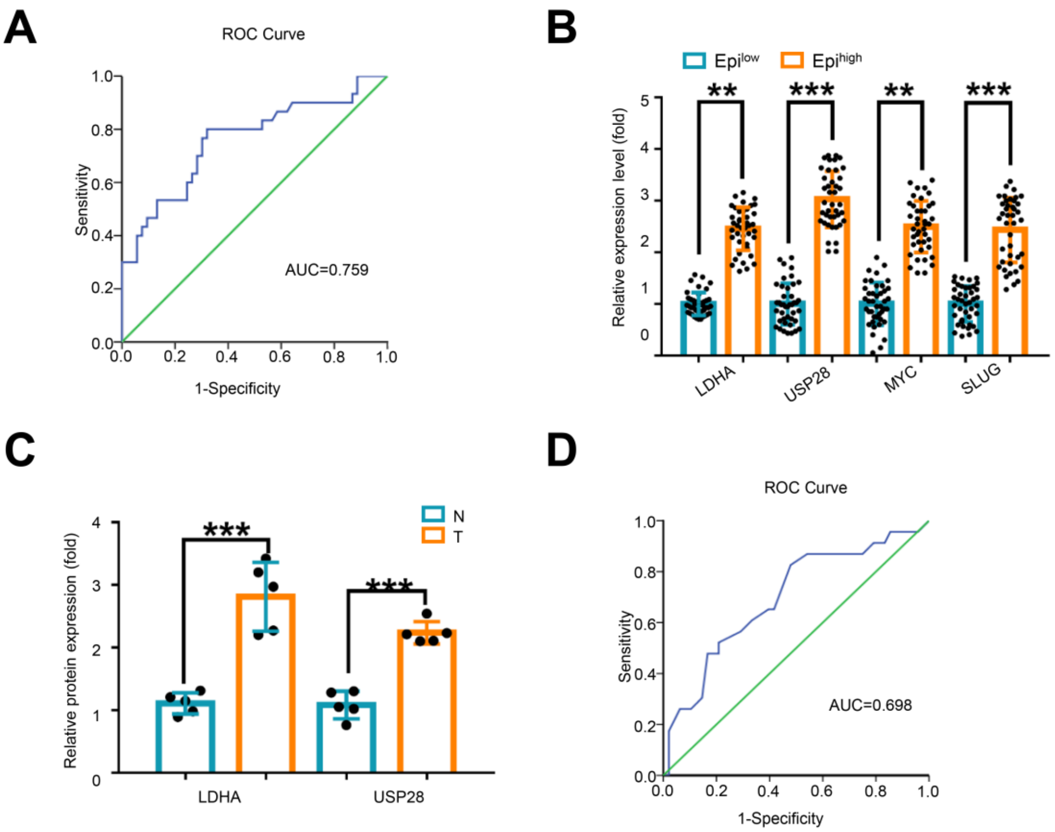

Supplemental Figure 7. Chronic stress is related to poor clinical outcome in patients with breast cancer. (A) Receiver operating characteristic (ROC) curve of sensitivity versus specificity of Epi^{high} (n=41) and Epi^{low} (n=42) groups. **(**B**)**Statistical analysis to determine the correlation between serum Epi levels and LDHA, USP28, MYC and SLUG from the IHC staining score in human breast cancer tissues. Epi^{high} (n=41) and Epi^{low} (n=42) (Student's t test). (C) Relative fold-changes of selected proteins in patient tissues. n=5 (Student's *t* test). (D) ROC curve of sensitivity versus specificity of LDHA^{high} (n=30) and LDHA^{low} (n=41) groups. Data are representative of at least 3 independent experiments.Data represent mean ± SEM, ***p < 0.001.

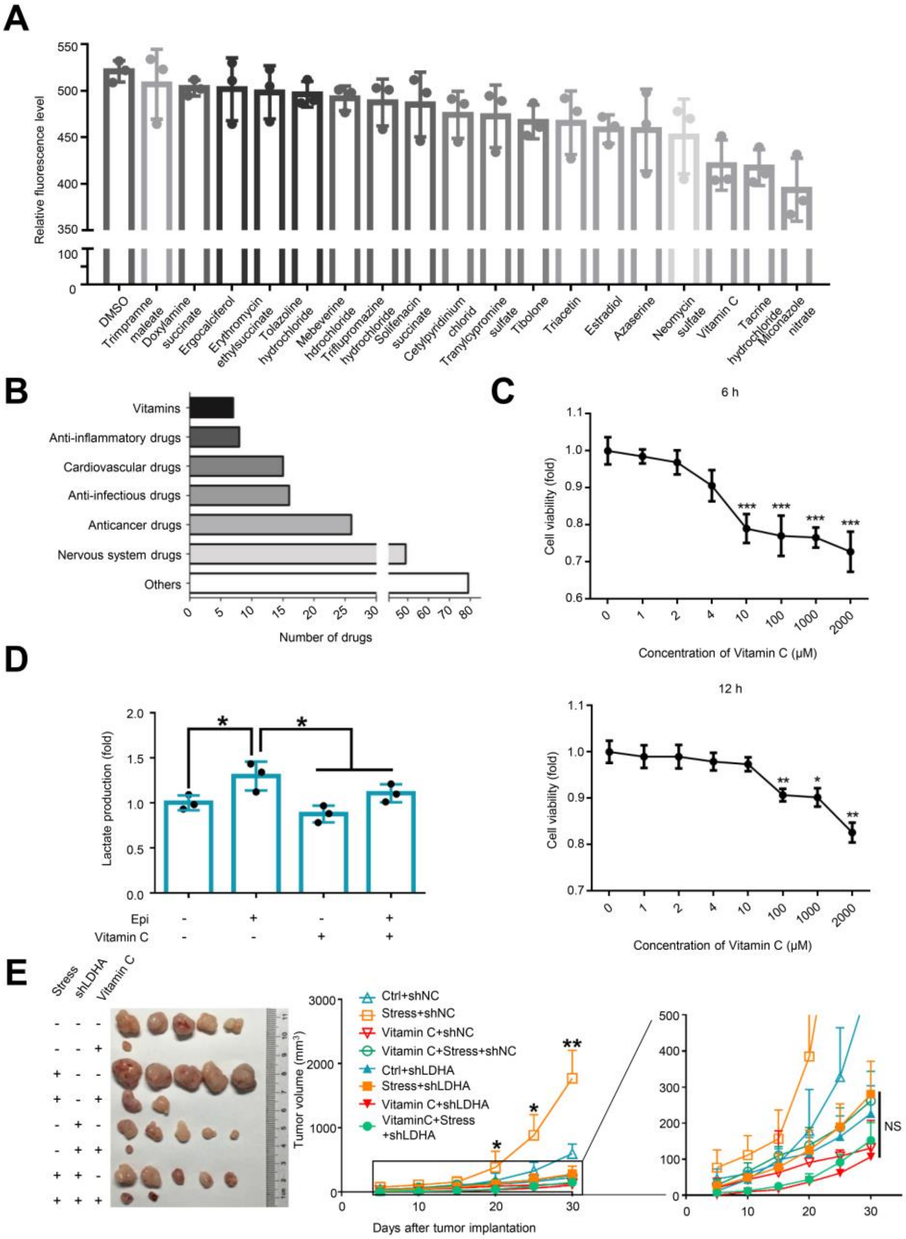

Supplemental Figure 8. Vitamin C is identified as the agent targeting LDHA. (A) Relative fold-change of the top 18 drugs that showed a reduction in LDHA. (n=3) (B) The top 200 hits were classified into different groups according to drug indications or functions during the virtual screening of 2,037 FDA-approved drugs against LDHA. (C) Cell viability of MDA-MB-231 cells treated with the indicated doses of vitamin C. n=3 (Student's *t* test). (D) Lactate was examined using cell culture medium from MCF-7 cells. n=3 (Student's *t* test). (E) Representative tumor images derived from treated mice (left panel) and tumor growth curves (right panel). n=5 (1-way ANOVA). Data are representative of at least3 independent experiments. Data represent mean ± SEM, *p < 0.05, **p < 0.01, ***p < 0.001.

Supplemental Movie 1. MD simulation on USP28^{WT} complexed with MYC$^{46\text{-}74}$ motif.
USP28^{WT} was depicted in gray and MYC$^{46\text{-}74}$ motif was shown in green. The Cys/Ala^{171} mutation site was displayed in red, and Lys^{51} and Lys^{52} in the MYC motif were depicted as blue spheres. States were collected for 0–150 ns after each ns.

Supplemental Movie 2. MD simulation for mutant USP28^{C171A} complexed with MYC$^{46\text{-}74}$ motif.
USP28^{C171A} was depicted in gray and MYC$^{46\text{-}74}$ motif was shown in green. The Cys/Ala^{171} mutation site was displayed in red, and Lys^{51} and Lys^{52} in the MYC motif were depicted as blue spheres. States were collected for 0–150 ns after each ns.

Supplemental Movie 3. MD simulation of Vitamin C complexed in the pocket of LDHA.
LDHA was depicted in gray, co-factor was depicted in stick model and vitamin C was depicted in gold sphere model. States were collected for 0–50 ns after each ns.

Full unedited gel for Figure 1B

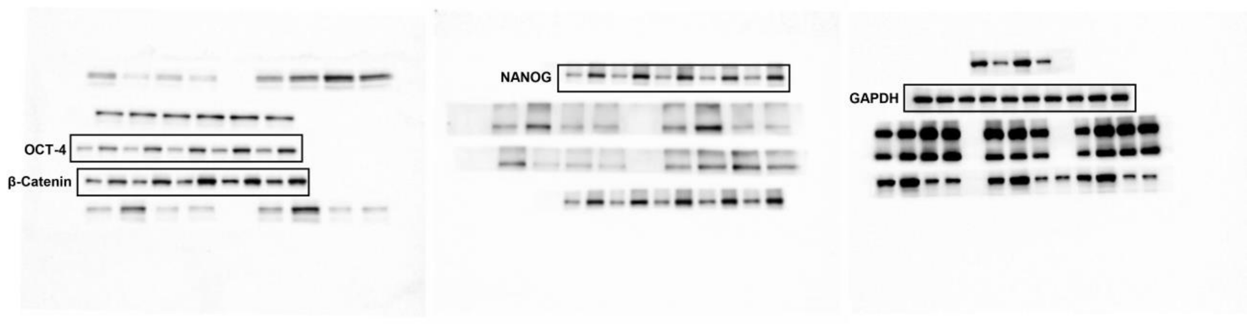

Full unedited gel for Figure 1F

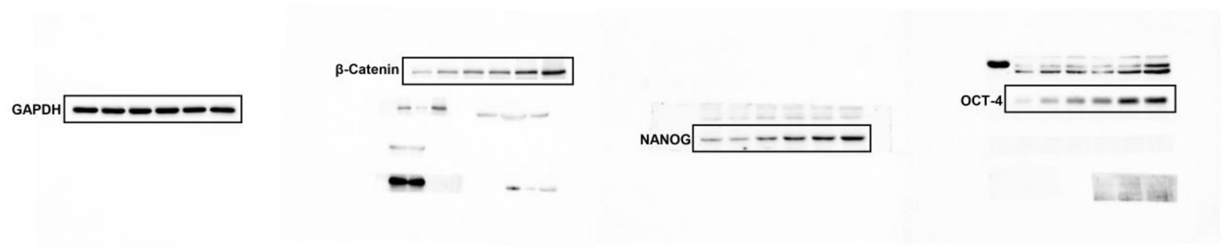

Full unedited gel for Figure 1H

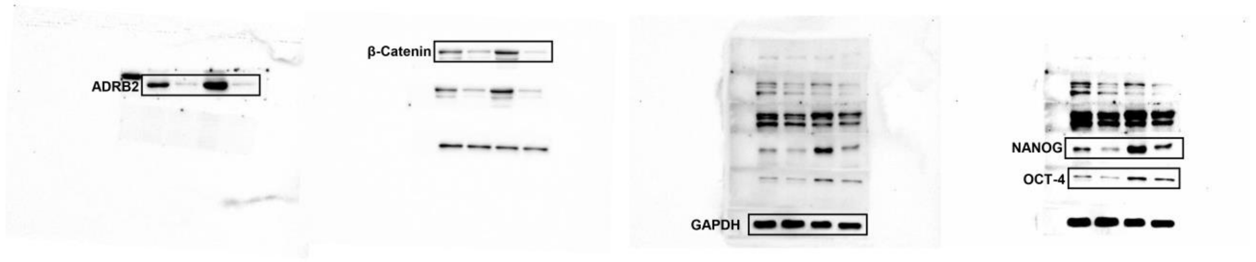

Full unedited gel for Figure 3A

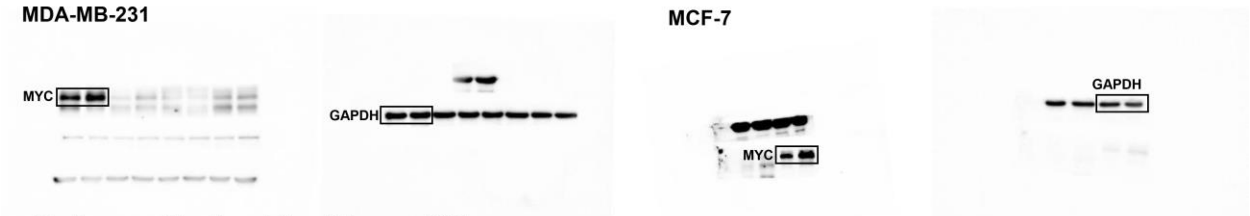

Full unedited gel for Figure 3C

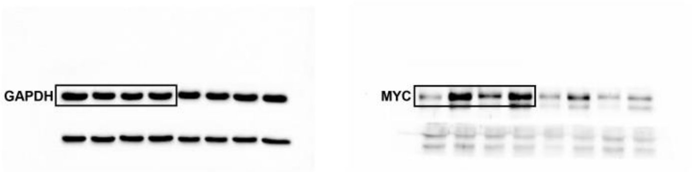

Full unedited gel for Figure 3E

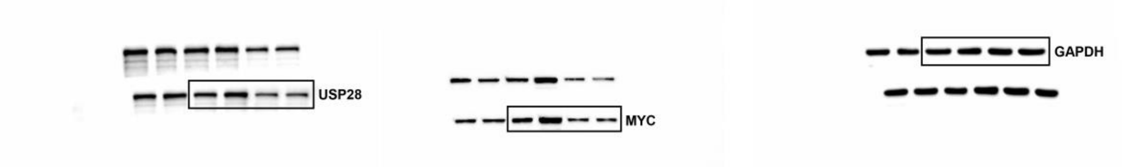

Full unedited gel for Figure 3F

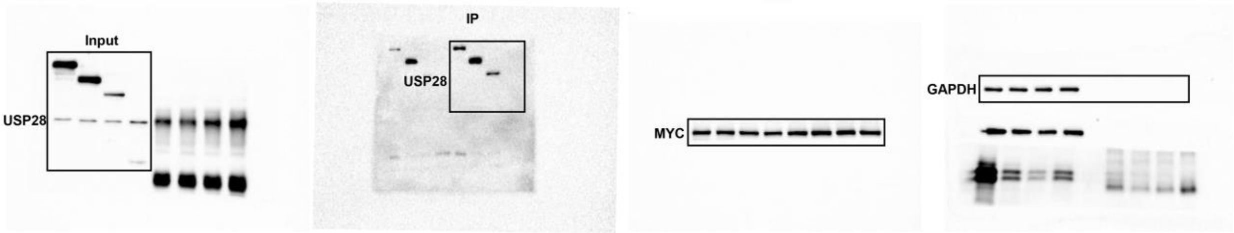

Full unedited gel for Figure 3H

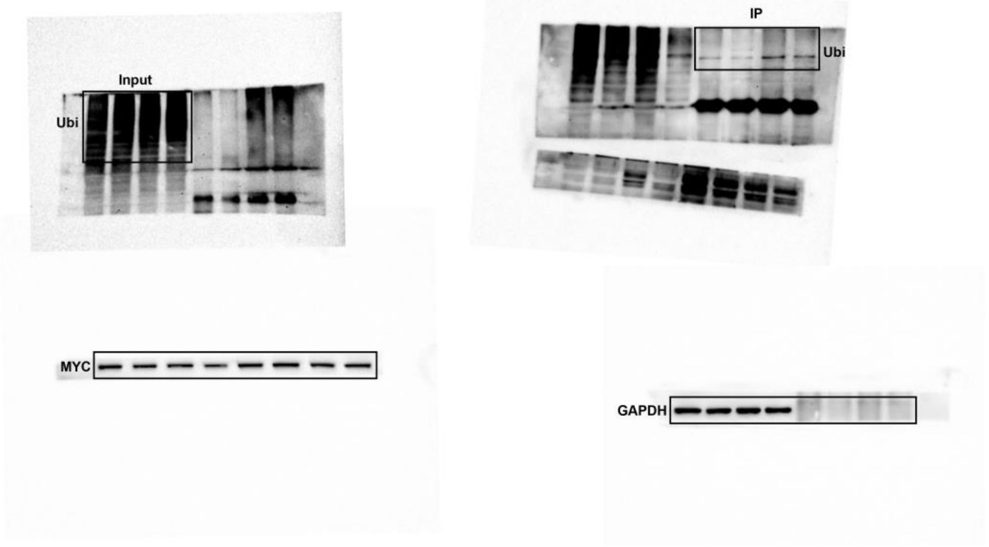

Full unedited gel for Figure 4D

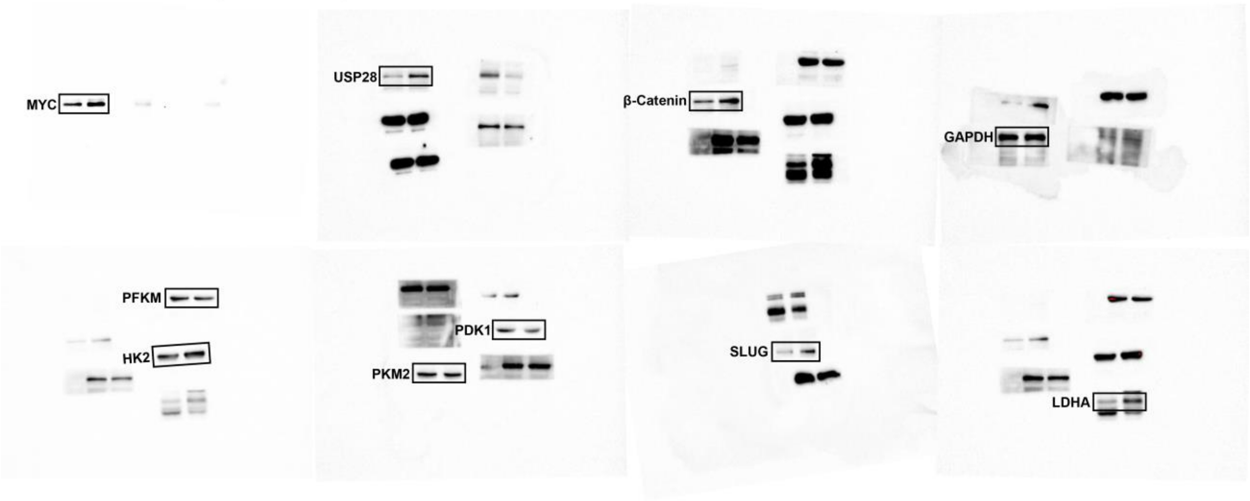

Full unedited gel for Figure 4E

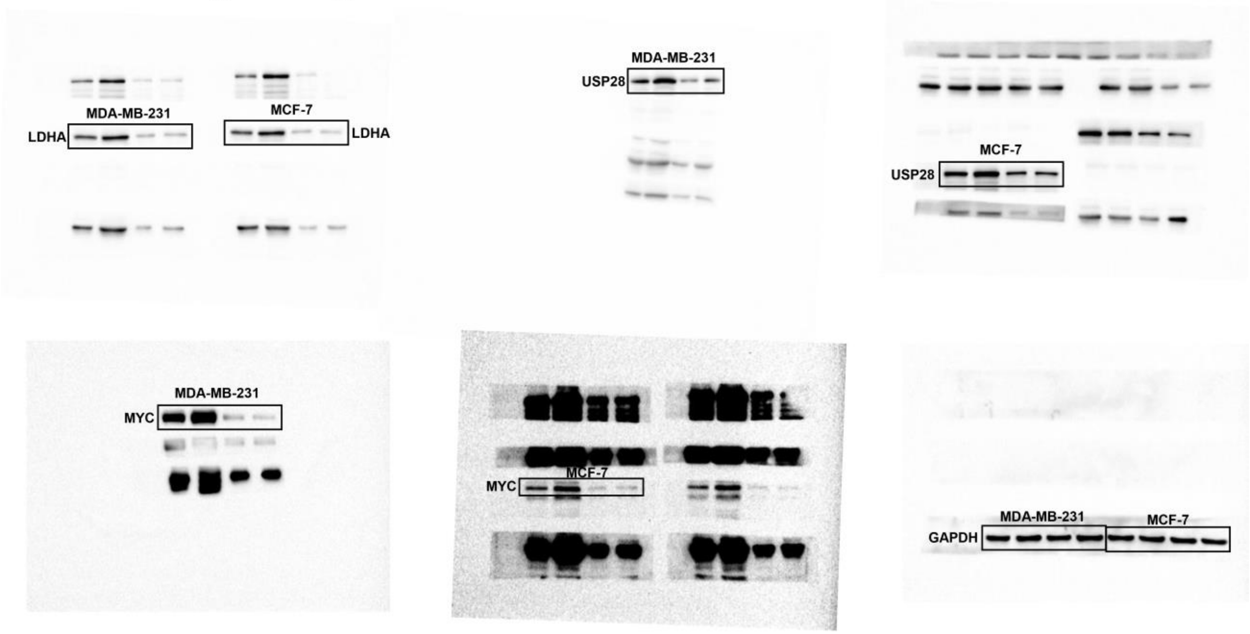

Full unedited gel for Figure 5A

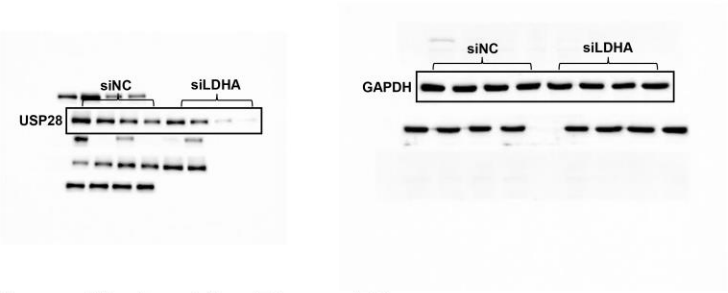

Full unedited gel for Figure 5B

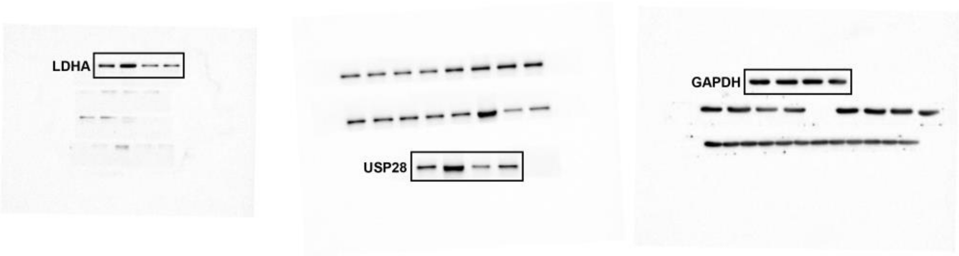

Full unedited gel for Figure 5C

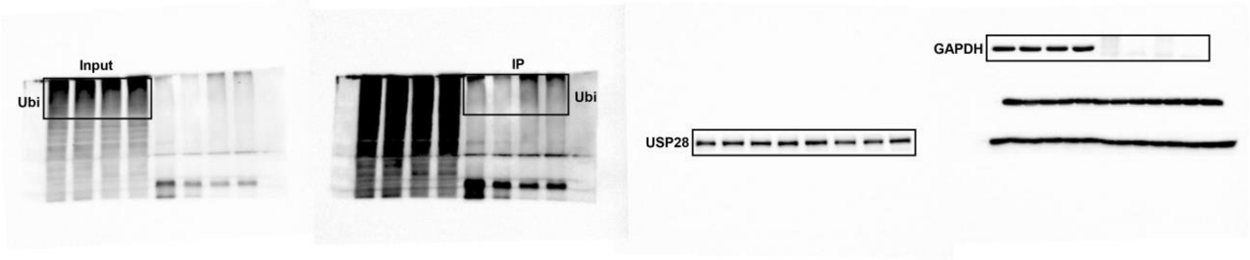

Full unedited gel for Figure 5D

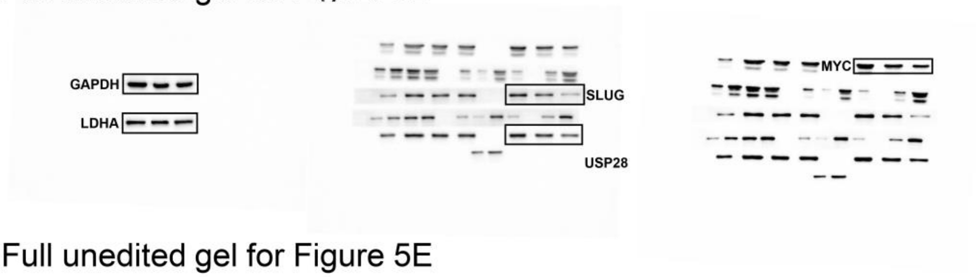

Full unedited gel for Figure 5E

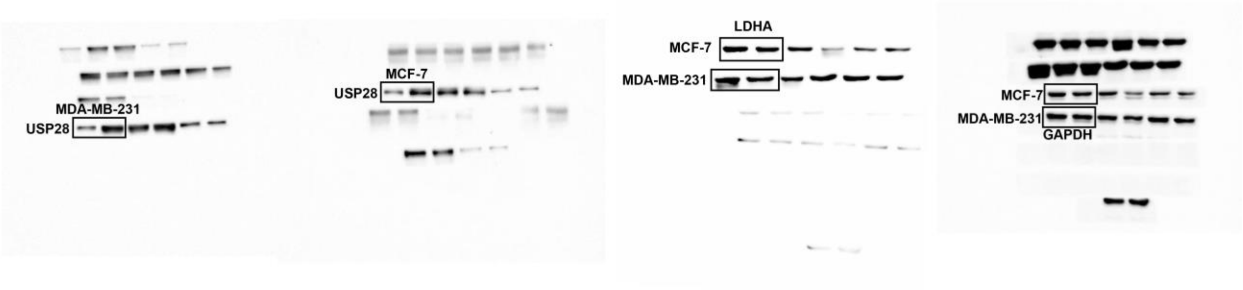

Full unedited gel for Figure 5F

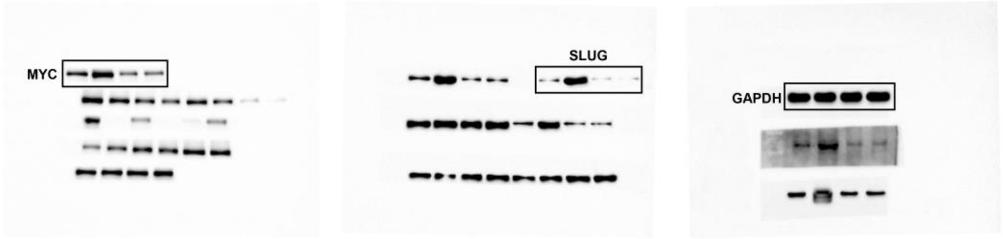

Full unedited gel for Figure 5G

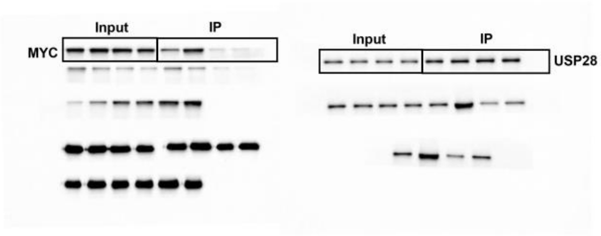

Full unedited gel for Figure 5H

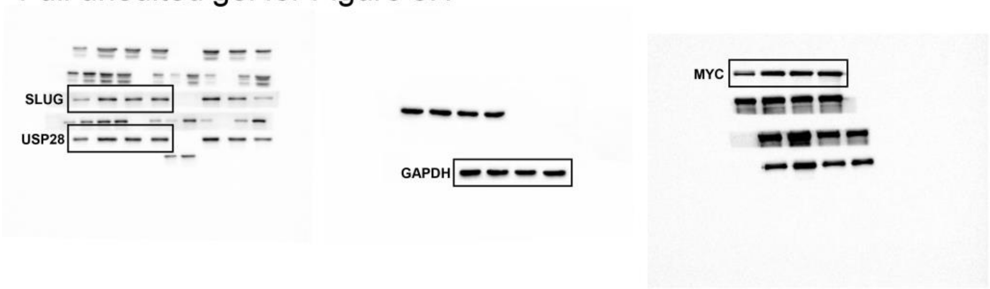

Full unedited gel for Figure 6B

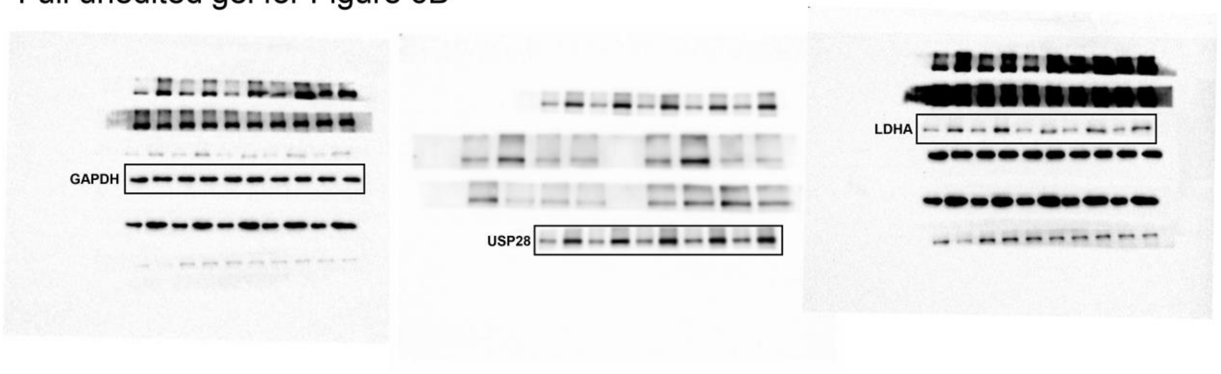

Full unedited gel for Figure 7C

MDA-MB-231

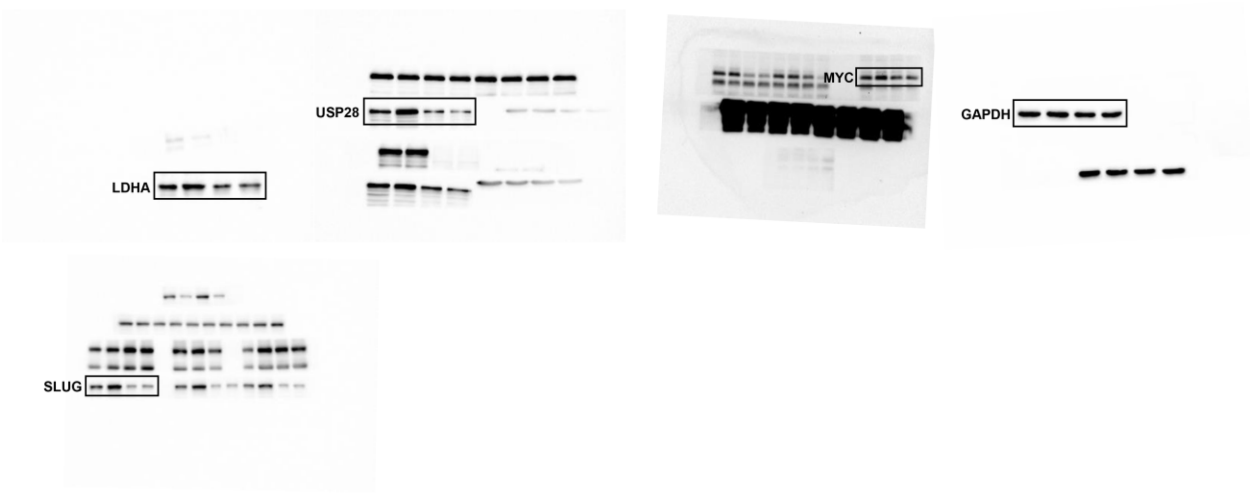

MCF-7

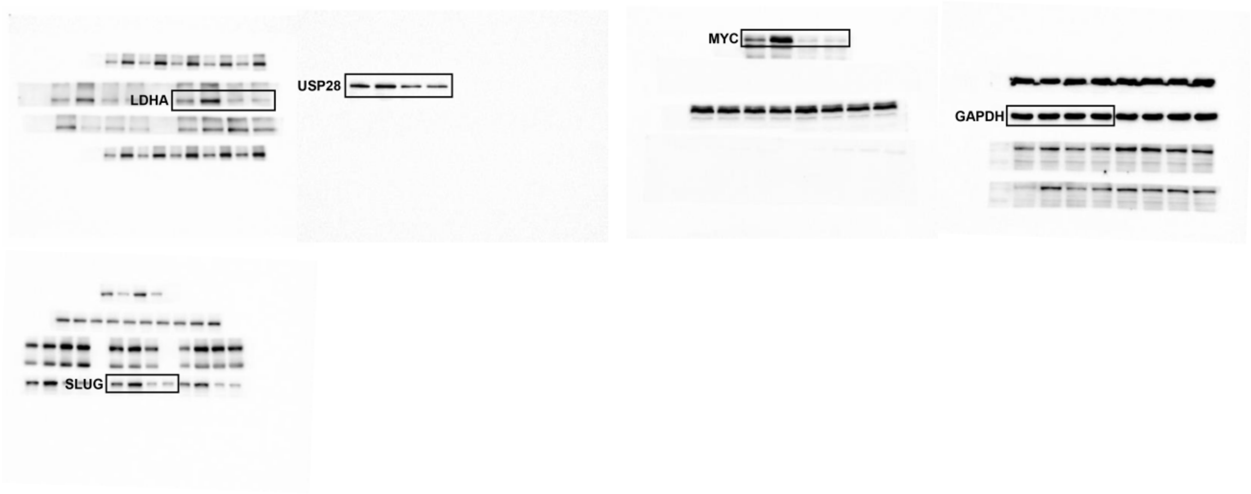

Full unedited gel for Supplemental Figure 2C

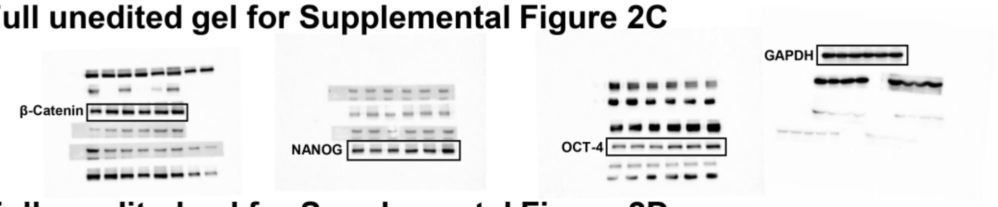

Full unedited gel for Supplemental Figure 2D

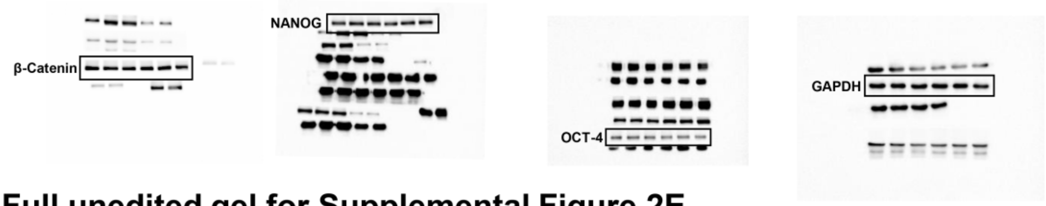

Full unedited gel for Supplemental Figure 2E

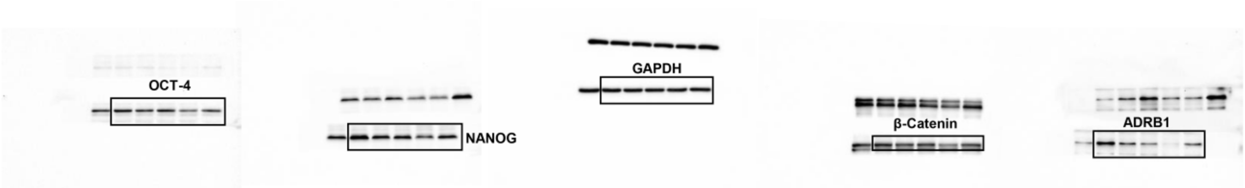

Full unedited gel for Supplemental Figure 2F

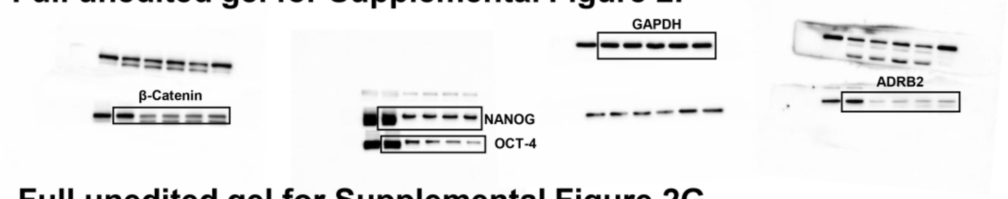

Full unedited gel for Supplemental Figure 2G

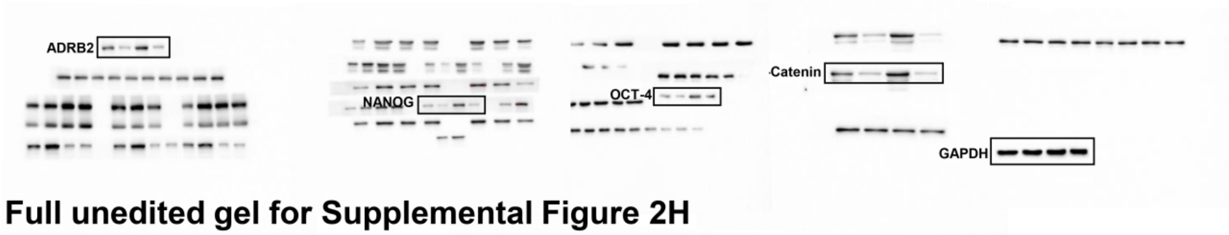

Full unedited gel for Supplemental Figure 2H

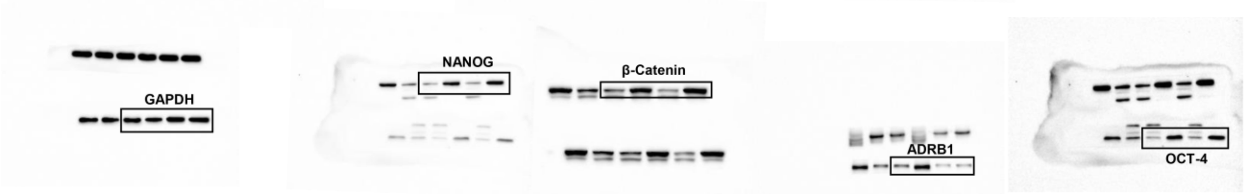

Full unedited gel for Supplemental Figure 2I

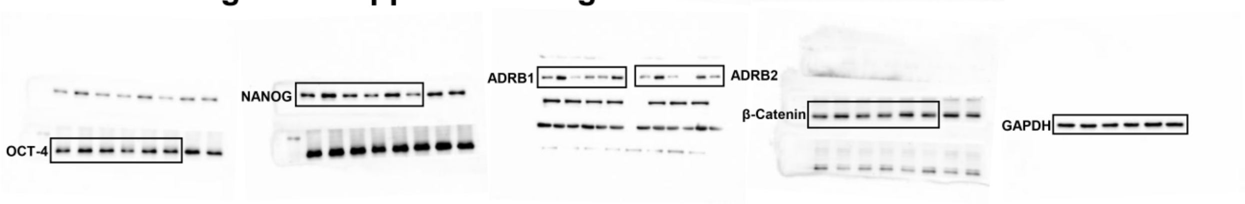

Full unedited gel for Supplemental Figure 3B

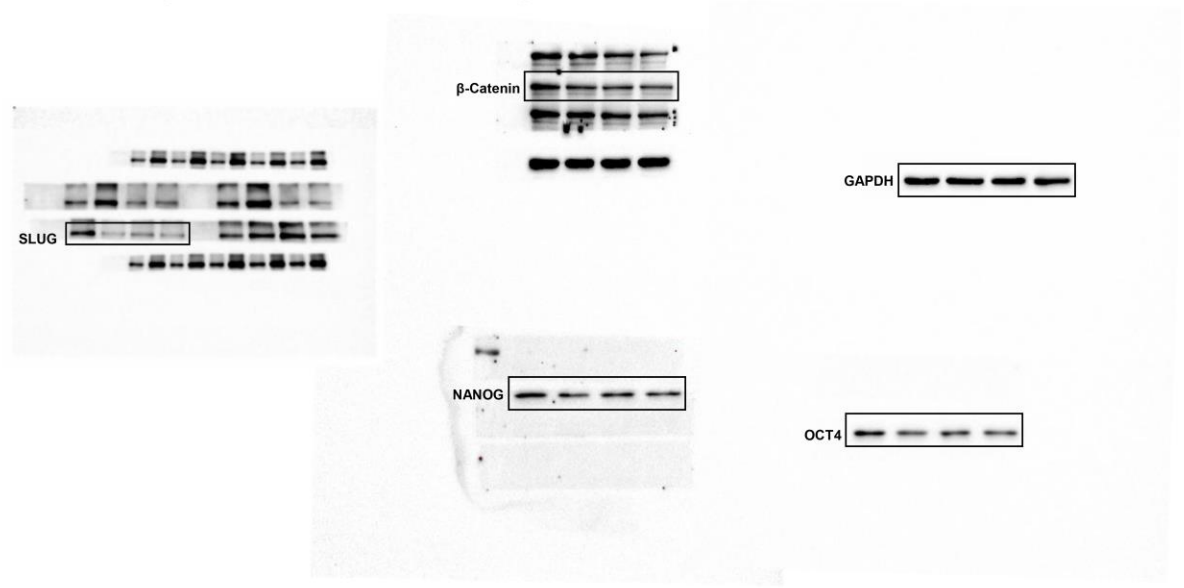

Full unedited gel for Supplemental Figure 3D

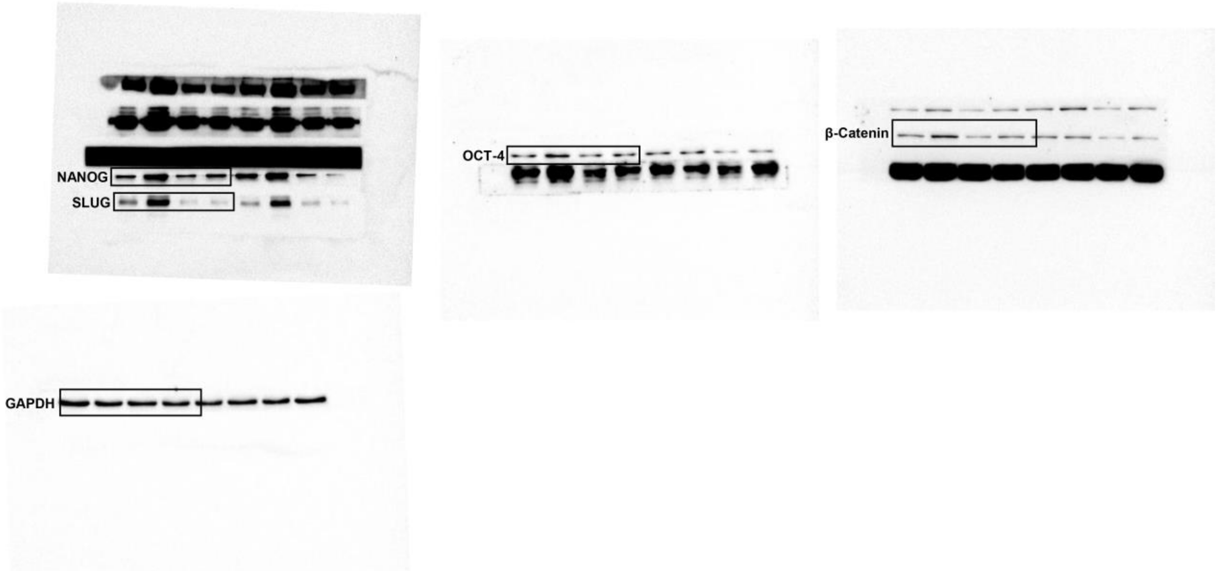

Full unedited gel for Supplemental Figure 3H

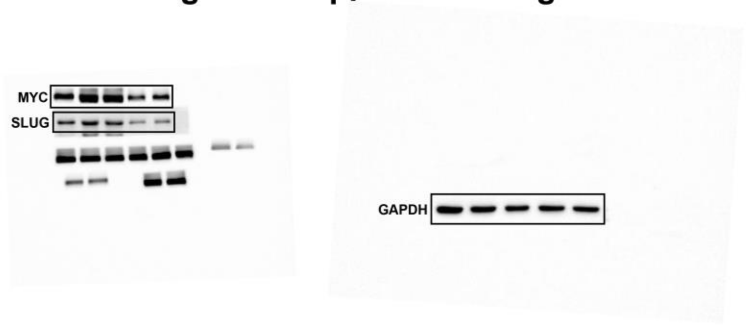

Full unedited gel for Supplemental Figure 4F

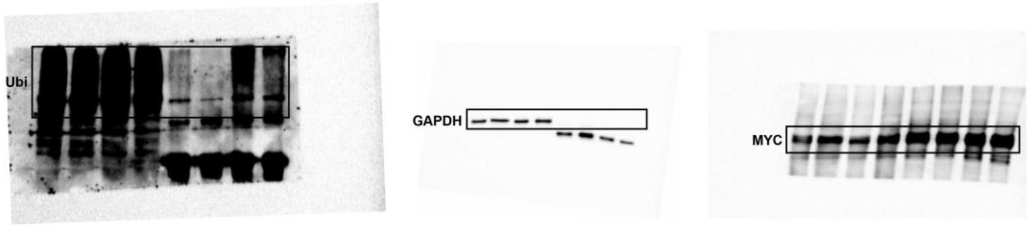

Full unedited gel for Supplemental Figure 4G

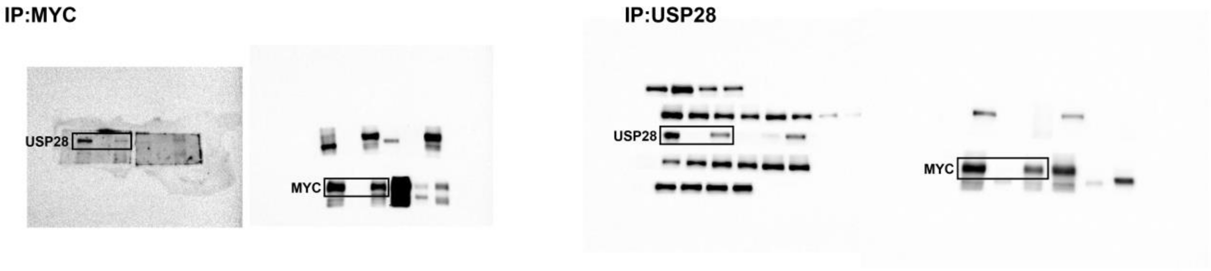

Full unedited gel for Supplemental Figure 4J

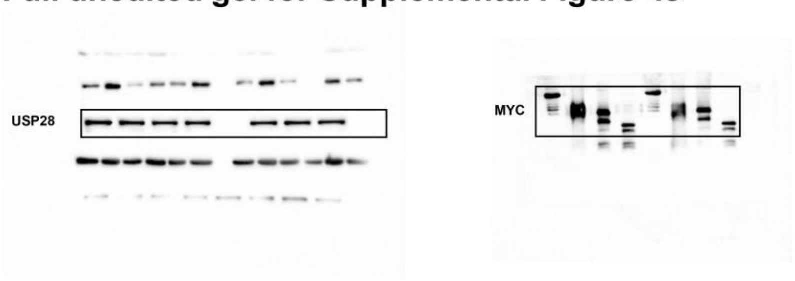

Full unedited gel for Supplemental Figure 4K

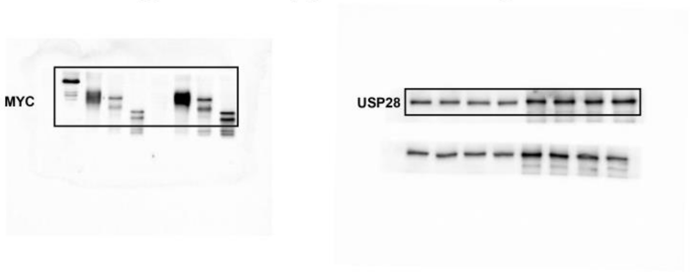

Full unedited gel for Supplemental Figure 5C

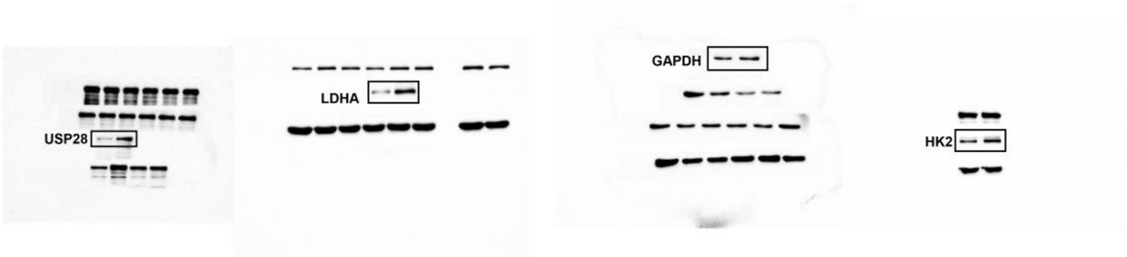

Full unedited gel for Supplemental Figure 5D

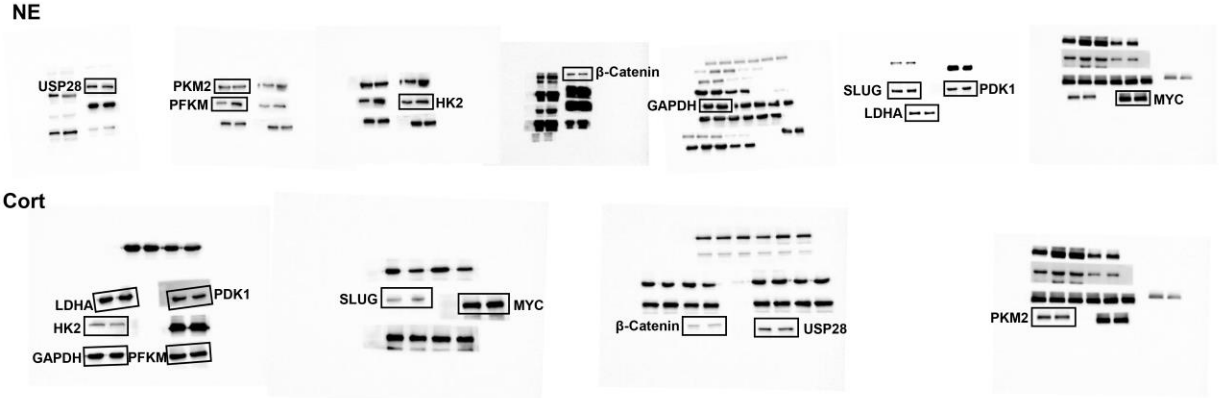

Full unedited gel for Supplemental Figure 5E

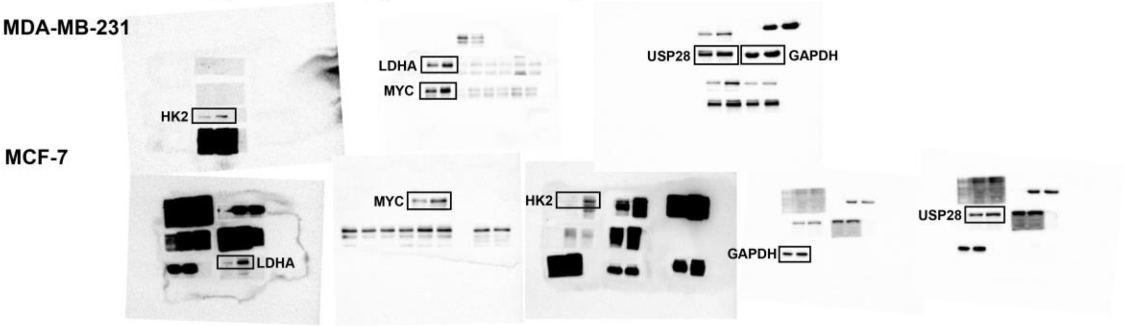

Full unedited gel for Supplemental Figure 5F

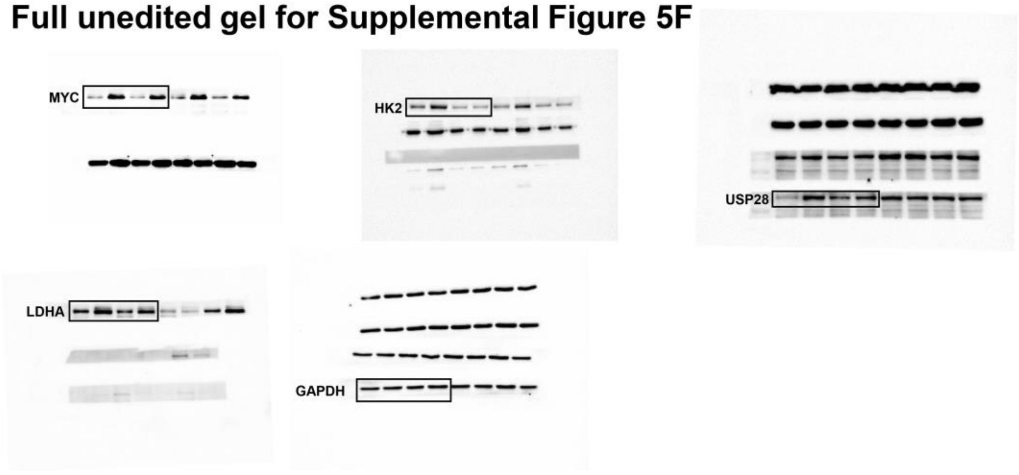

Full unedited gel for Supplemental Figure 6B

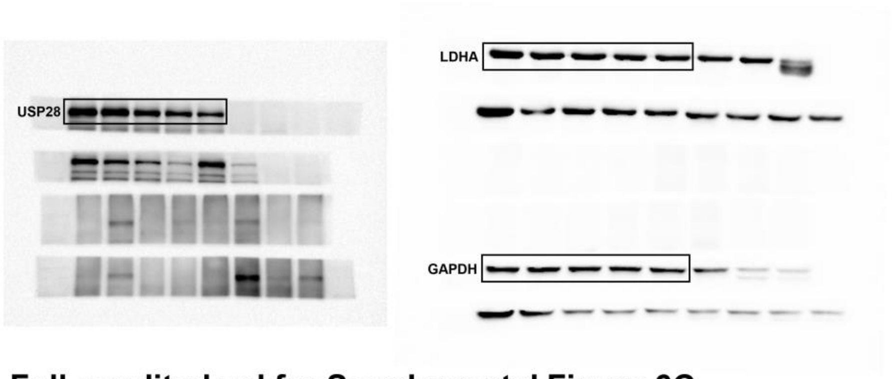

Full unedited gel for Supplemental Figure 6C

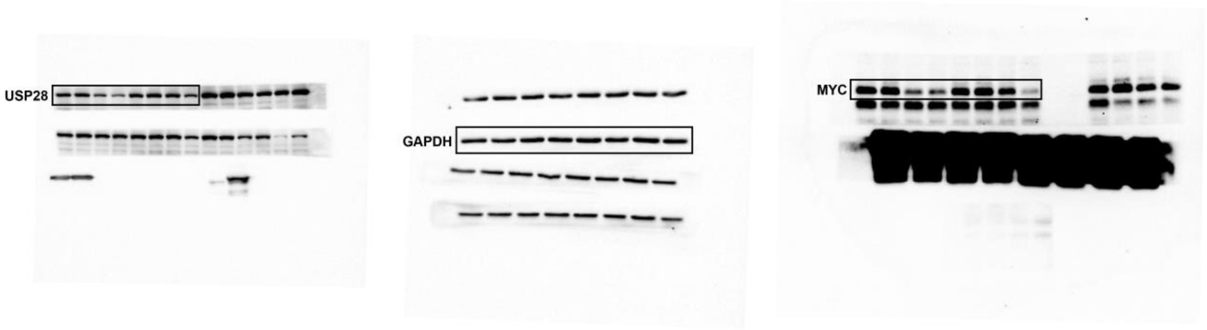

Full unedited gel for Supplemental Figure 6D

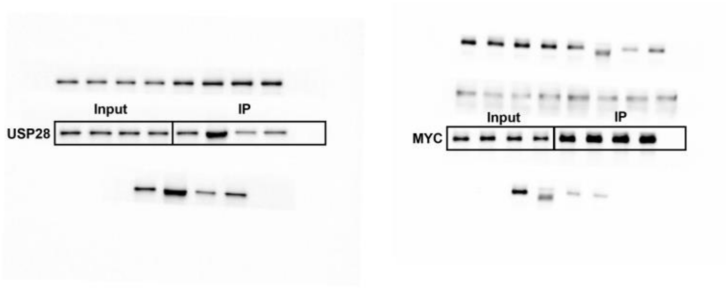

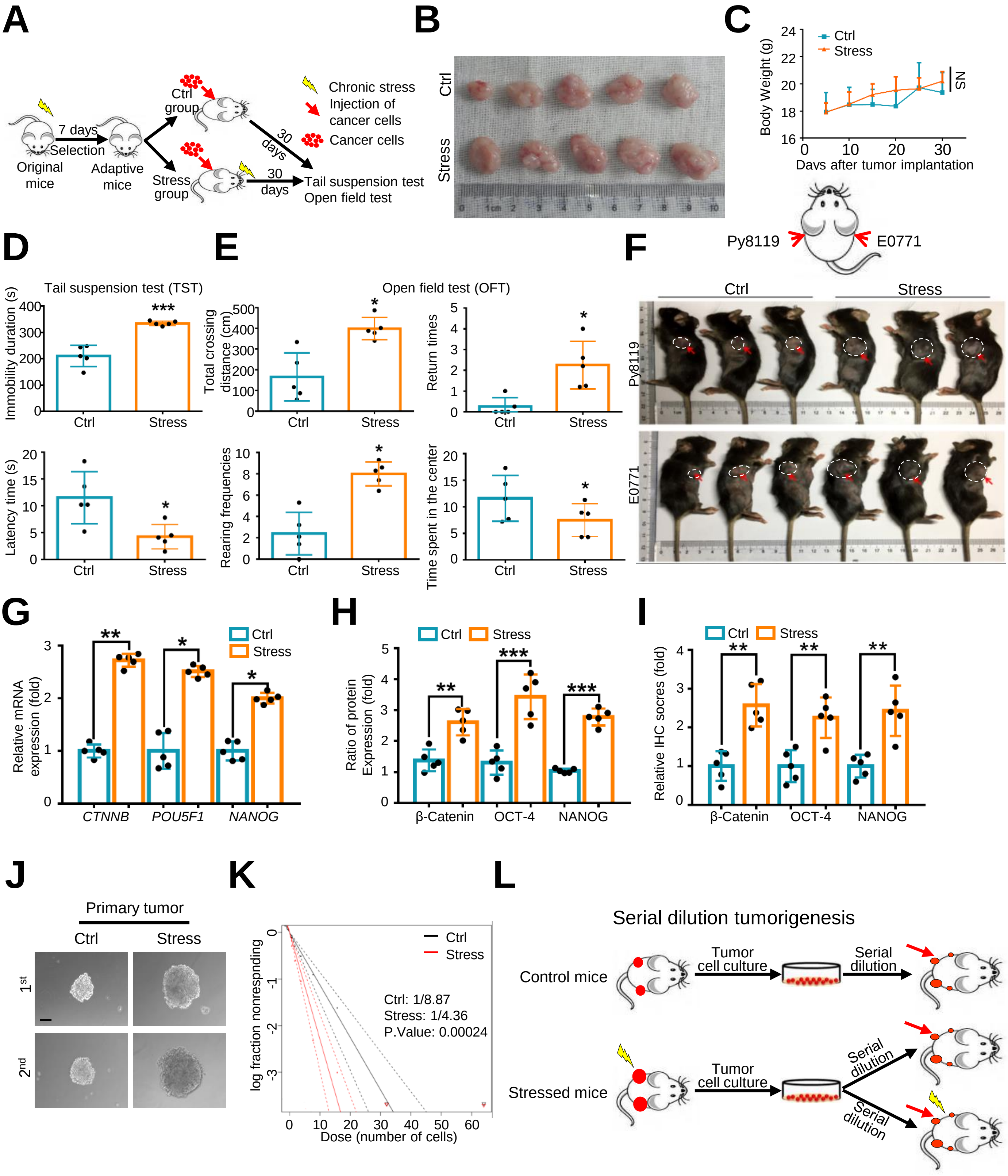

A
7 days
Selection
Original mice
Adaptive mice
Ctrl group
Stress group
30 days
30 days
Chronic stress
Injection of cancer cells
Cancer cells
Tail suspension test
Open field test
B
Ctrl
Stress
C
Ctrl
Stress
Body Weight (g)
Days after tumor implantation
NS
D
Tail suspension test (TST)
Immobility duration (s)
Latency time (s)
E
Open field test (OFT)
Total crossing distance (cm)
Return times
Rearing frequencies
Time spent in the center
F
Py8119
E0771
G
Relative mRNA expression (fold)
CTNNB
POU5F1
NANOG
H
Ratio of protein Expression (fold)
β-Catenin
OCT-4
NANOG
I
Relative IHC socres (fold)
J
Primary tumor
1st
2nd
K
log fraction nonrespnding
Dose (number of cells)
Ctrl: 1/8.87
Stress: 1/4.36
P.Value: 0.00024
L
Serial dilution tumorigenesis
Control mice
Stressed mice
Tumor cell culture
Serial dilution

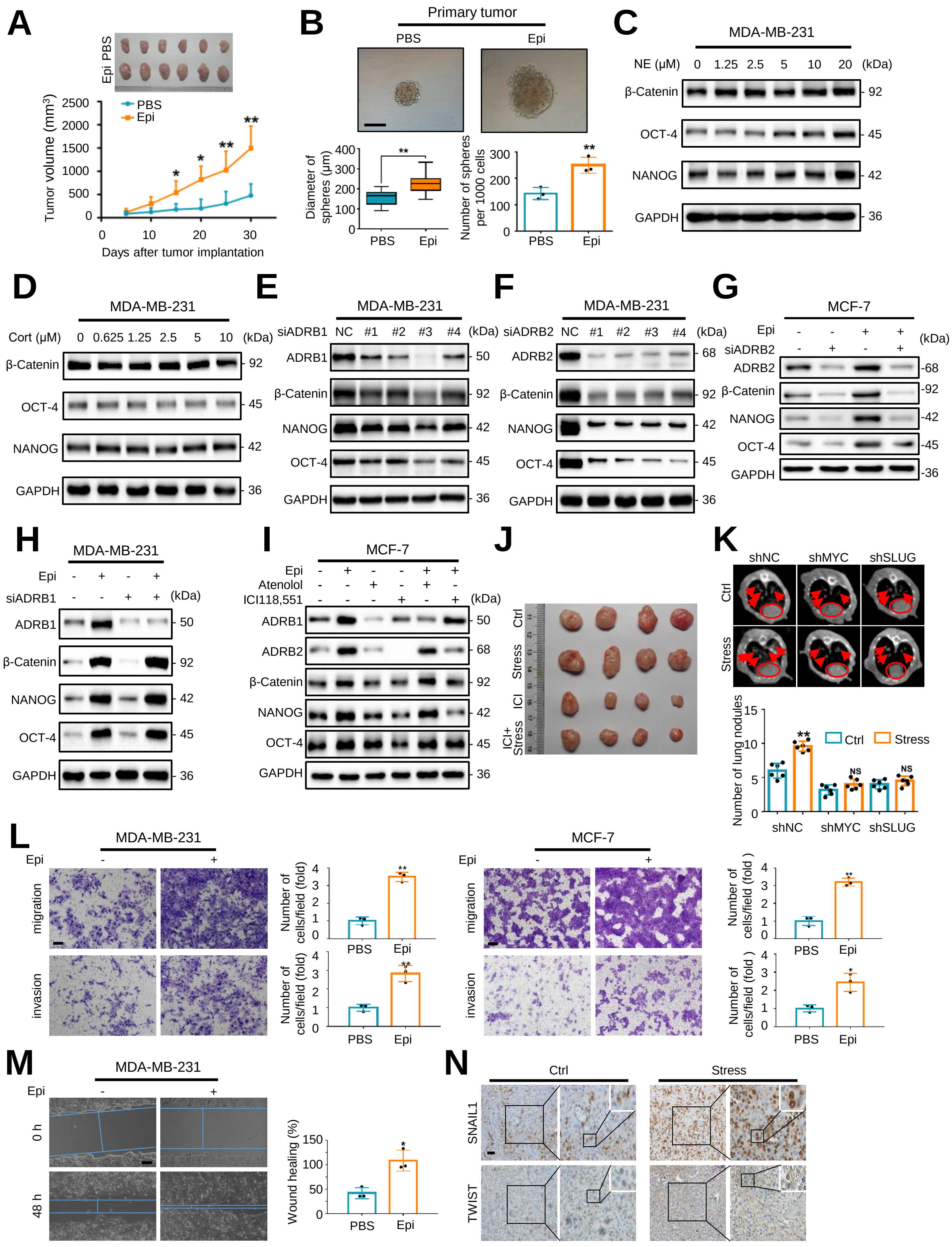

A
Epi PBS
Tumor volume (mm3)
PBS
Epi
Days after tumor implantation
B
Primary tumor
PBS
Epi
Diameter of spheres (μm)
Number of spheres per 1000 cells
C
MDA-MB-231
NE (μM) 0 1.25 2.5 5 10 20 (kDa)
β-Catenin 92
OCT-4 45
NANOG 42
GAPDH 36
D
MDA-MB-231
Cort (μM) 0 0.625 1.25 2.5 5 10 (kDa)
E
MDA-MB-231
siADRB1 NC #1 #2 #3 #4 (kDa)
ADRB1 50
F
MDA-MB-231
siADRB2 NC #1 #2 #3 #4 (kDa)
ADRB2 68
G
MCF-7
Epi
siADRB2
H
MDA-MB-231
Epi
siADRB1
I
MCF-7
Epi
Atenolol
ICI118,551
J
Ctrl
Stress
ICI
ICI+ Stress
K
shNC shMYC shSLUG
Ctrl
Stress
Number of lung nodules
NS
L
MDA-MB-231
MCF-7
migration
invasion
Number of cells/field (fold)
M
MDA-MB-231
0 h
48 h
Wound healing (%)
N
Ctrl
Stress
SNAIL1
TWIST

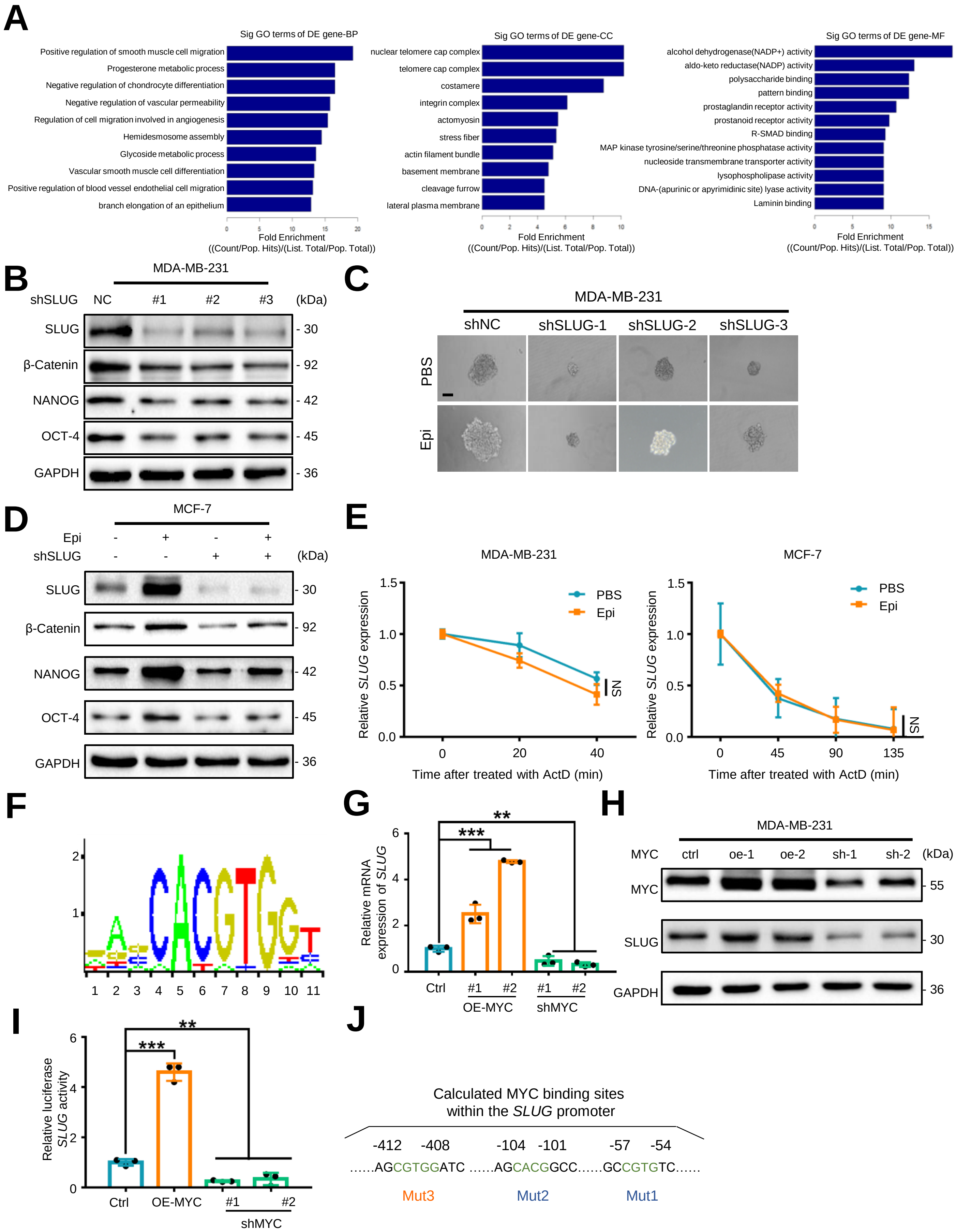

A
Sig GO terms of DE gene-BP
Positive regulation of smooth muscle cell migration
Progesterone metabolic process
Negative regulation of chondrocyte differentiation
Negative regulation of vascular permeability
Regulation of cell migration involved in angiogenesis
Hemidesmosome assembly
Glycoside metabolic process
Vascular smooth muscle cell differentiation
Positive regulation of blood vessel endothelial cell migration
branch elongation of an epithelium
Fold Enrichment
((Count/Pop. Hits)/(List. Total/Pop. Total))
Sig GO terms of DE gene-CC
nuclear telomere cap complex
telomere cap complex
costamere
integrin complex
actomyosin
stress fiber
actin filament bundle
basement membrane
cleavage furrow
lateral plasma membrane
Sig GO terms of DE gene-MF
alcohol dehydrogenase(NADP+) activity
aldo-keto reductase(NADP) activity
polysaccharide binding
pattern binding
prostaglandin receptor activity
prostanoid receptor activity
R-SMAD binding
MAP kinase tyrosine/serine/threonine phosphatase activity
nucleoside transmembrane transporter activity
lysophospholipase activity
DNA-(apurinic or apyrimidinic site) lyase activity
Laminin binding
B
MDA-MB-231
shSLUG NC #1 #2 #3 (kDa)
SLUG - 30
β-Catenin - 92
NANOG - 42
OCT-4 - 45
GAPDH - 36
C
MDA-MB-231
shNC shSLUG-1 shSLUG-2 shSLUG-3
PBS
Epi
D
MCF-7
Epi - + - +
shSLUG - - + + (kDa)
SLUG - 30
β-Catenin - 92
NANOG - 42
OCT-4 - 45
GAPDH - 36
E
MDA-MB-231
MCF-7
PBS
Epi
Relative SLUG expression
NS
Time after treated with ActD (min)
F
G
Relative mRNA expression of SLUG
***
**
Ctrl #1 #2 #1 #2
OE-MYC shMYC
H
MDA-MB-231
MYC ctrl oe-1 oe-2 sh-1 sh-2 (kDa)
MYC - 55
SLUG - 30
GAPDH - 36
I
Relative luciferase SLUG activity
***
**
Ctrl OE-MYC #1 #2
shMYC
J
Calculated MYC binding sites within the SLUG promoter
-412 -408 -104 -101 -57 -54
……AGCGTGGATC ……AGCACGGCC……GCCGTGTC……
Mut3 Mut2 Mut1

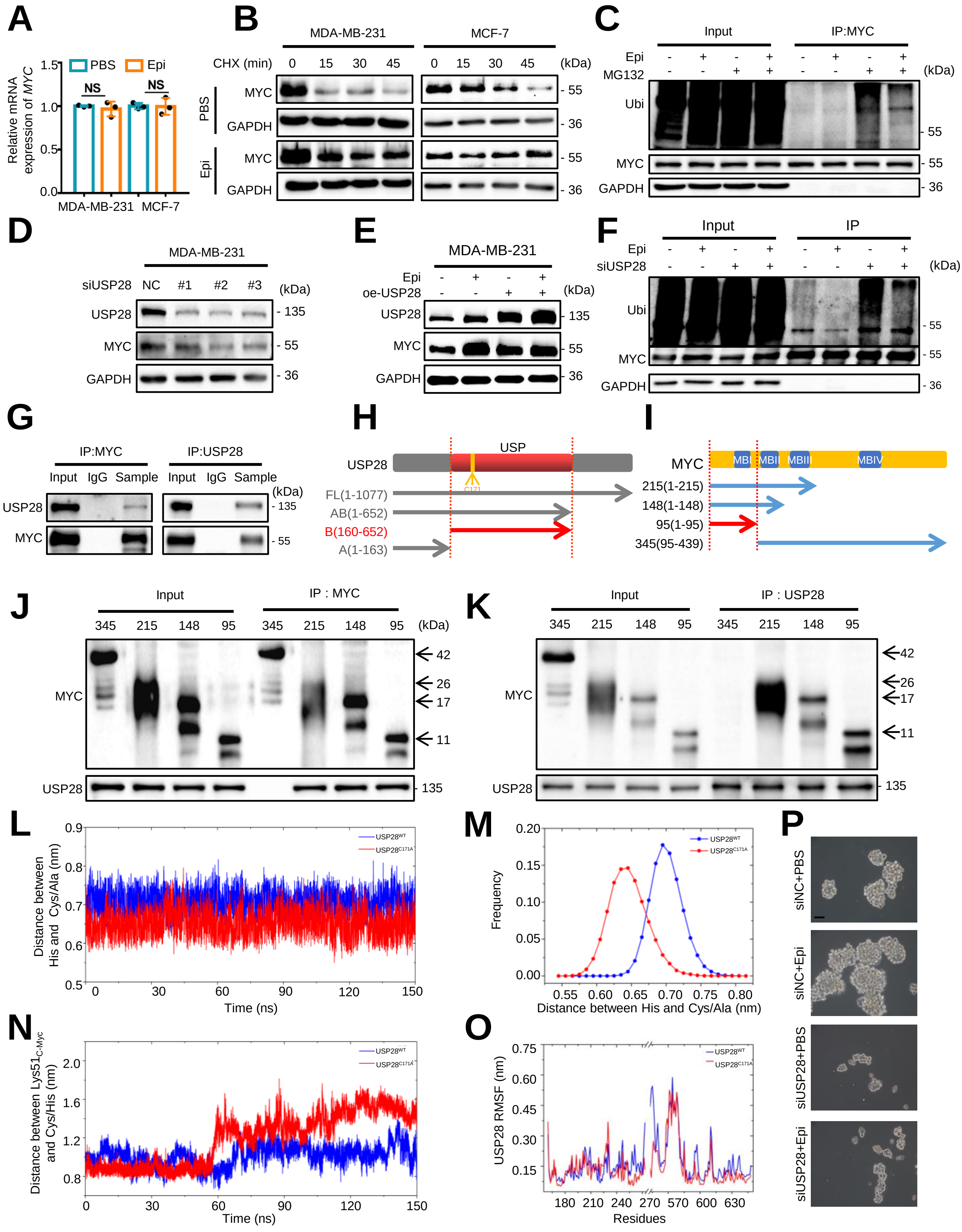
A
B
C
D
E
F
G
H
I
J
K
L
M
N
O
P
PBS
Epi
NS
Relative mRNA expression of MYC
MDA-MB-231
MCF-7
CHX (min)
0
15
30
45
(kDa)
MYC
GAPDH
Input
IP:MYC
MG132
Ubi
siUSP28
NC
#1
#2
#3
USP28
oe-USP28
IP
IP:USP28
IgG
Sample
USP
C171
FL(1-1077)
AB(1-652)
B(160-652)
A(1-163)
MBI
MBII
MBIII
MBIV
215(1-215)
148(1-148)
95(1-95)
345(95-439)
IP : MYC
IP : USP28
USP28WT
USP28C171A
Distance between His and Cys/Ala (nm)
Time (ns)
Frequency
Distance between Lys51C-Myc and Cys/His (nm)
USP28 RMSF (nm)
Residues
siNC+PBS
siNC+Epi
siUSP28+PBS
siUSP28+Epi

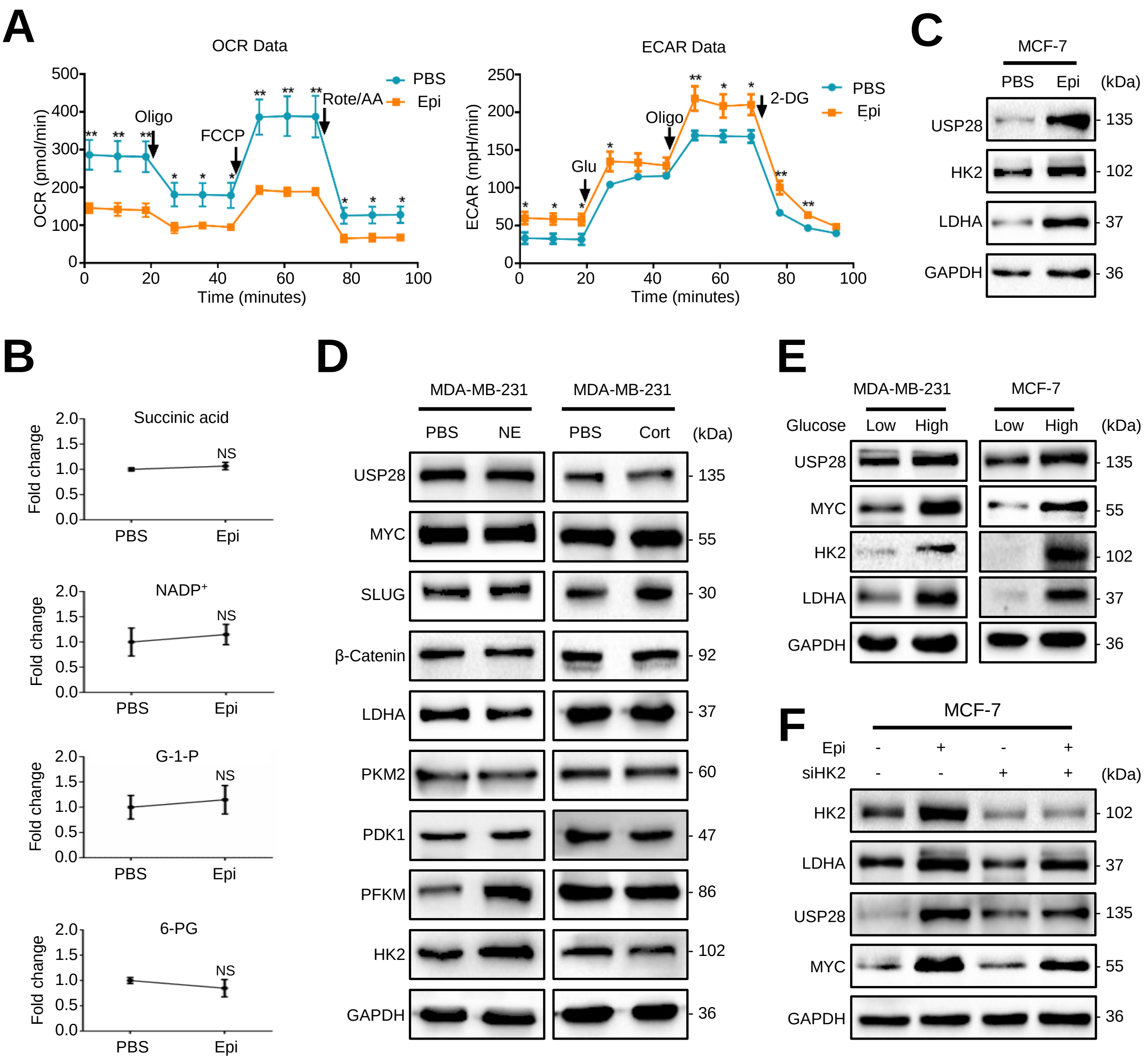
A
OCR Data
OCR (pmol/min)
Time (minutes)
Oligo
FCCP
Rote/AA
PBS
Epi
ECAR Data
ECAR (mpH/min)
Glu
Oligo
2-DG
B
Succinic acid
NADP+
G-1-P
6-PG
Fold change
NS
PBS
Epi
C
MCF-7
PBS
Epi
(kDa)
USP28
HK2
LDHA
GAPDH
135
102
37
36
D
MDA-MB-231
PBS
NE
Cort
USP28
MYC
SLUG
β-Catenin
LDHA
PKM2
PDK1
PFKM
HK2
GAPDH
135
55
30
92
37
60
47
86
102
36
E
MDA-MB-231
MCF-7
Glucose
Low
High
USP28
MYC
HK2
LDHA
GAPDH
F
MCF-7
Epi
siHK2
HK2
LDHA
USP28
MYC
GAPDH

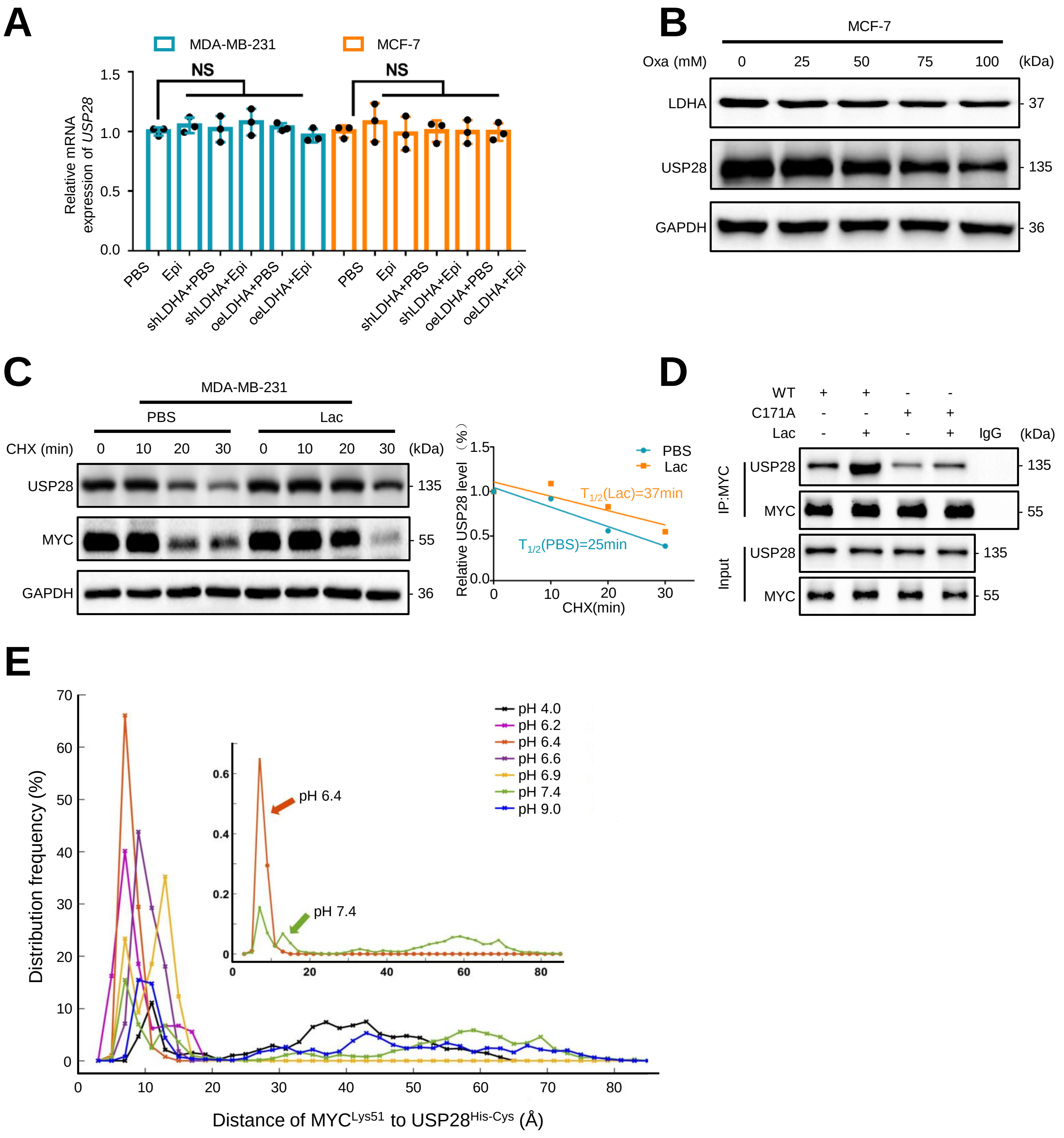

A
MDA-MB-231
MCF-7
NS
NS
Relative mRNA expression of USP28
PBS
Epi
shLDHA+PBS
shLDHA+Epi
oeLDHA+PBS
oeLDHA+Epi
B
MCF-7
Oxa (mM) 0 25 50 75 100 (kDa)
LDHA 37
USP28 135
GAPDH 36
C
MDA-MB-231
PBS
Lac
CHX (min) 0 10 20 30 0 10 20 30 (kDa)
USP28 135
MYC 55
GAPDH 36
Relative USP28 level (%)
PBS
Lac
T1/2(Lac)=37min
T1/2(PBS)=25min
CHX(min)
D
WT + + - -
C171A - - + +
Lac - + - +
IgG (kDa)
IP:MYC
USP28 135
MYC 55
Input
USP28 135
MYC 55
E
pH 4.0
pH 6.2
pH 6.4
pH 6.6
pH 6.9
pH 7.4
pH 9.0
pH 6.4
pH 7.4
Distribution frequency (%)
Distance of MYCLys51 to USP28His-Cys (Å)

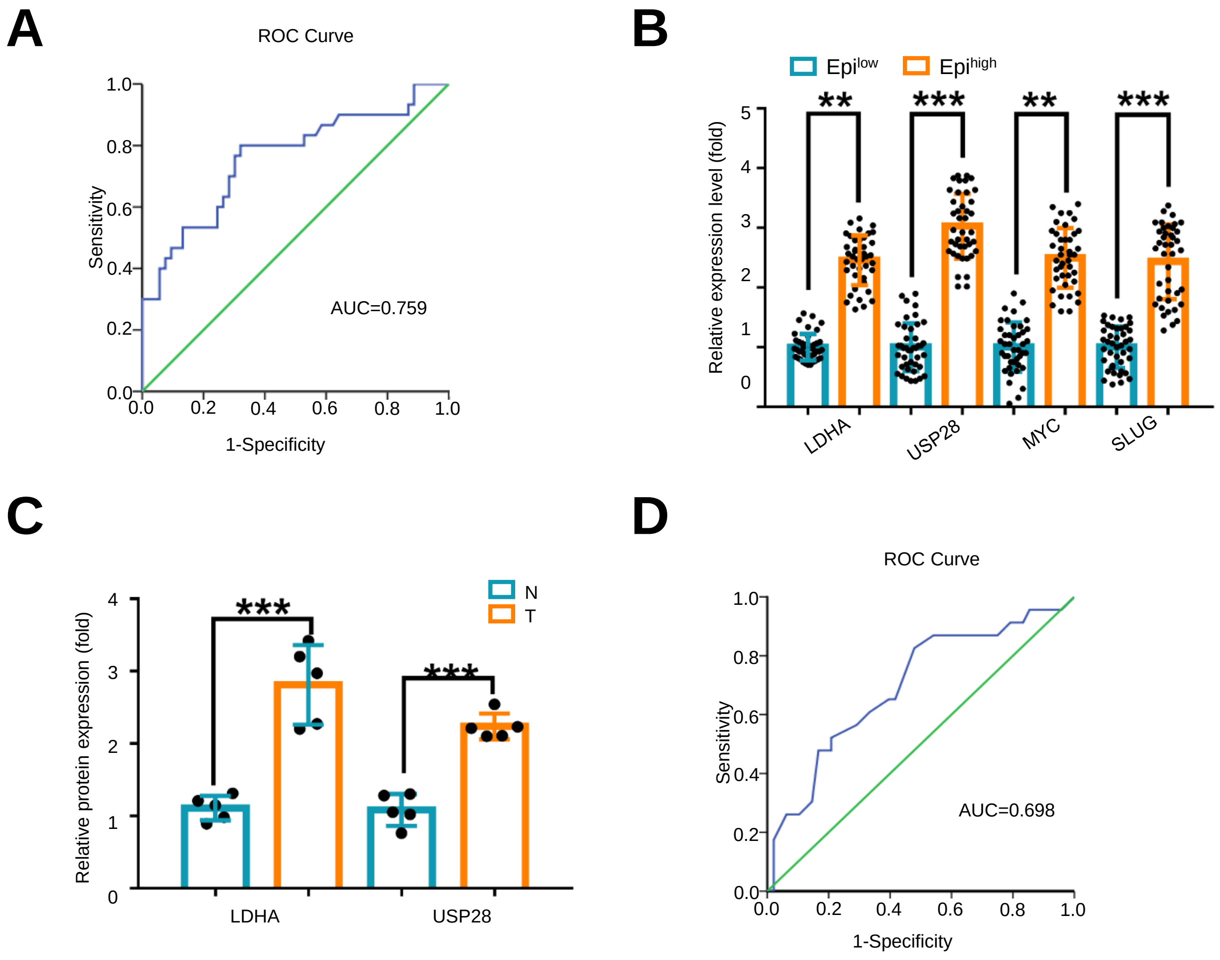
A
ROC Curve
Sensitivity
1-Specificity
AUC=0.759
B
Epi^low
Epi^high
Relative expression level (fold)
**
***
**
***
LDHA
USP28
MYC
SLUG
C
Relative protein expression (fold)
N
T
***
***
LDHA
USP28
D
ROC Curve
Sensitivity
1-Specificity
AUC=0.698

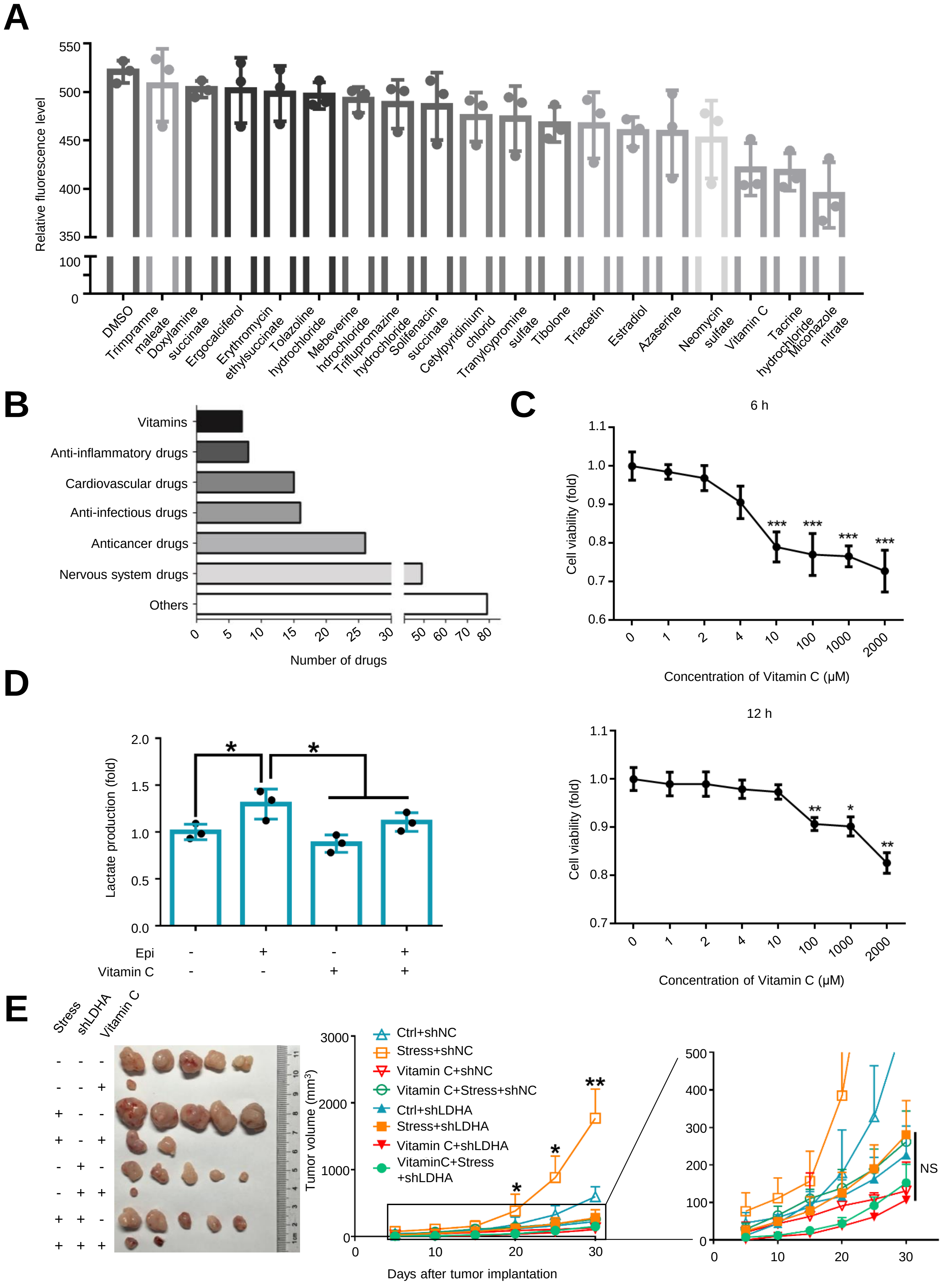
A
Relative fluorescence level
550
500
450
400
350
100
0
DMSO
Trimpramne maleate
Doxylamine succinate
Ergocalciferol
Erythromycin ethylsuccinate
Tolazoline hydrochloride
Mebeverine hdrochloride
Triflupromazine hydrochloride
Solifenacin succinate
Cetylpyridinium chlorid
Tranylcypromine sulfate
Tibolone
Triacetin
Estradiol
Azaserine
Neomycin sulfate
Vitamin C
Tacrine hydrochloride
Miconazole nitrate
B
Vitamins
Anti-inflammatory drugs
Cardiovascular drugs
Anti-infectious drugs
Anticancer drugs
Nervous system drugs
Others
Number of drugs
C
6 h
12 h
Cell viability (fold)
Concentration of Vitamin C (μM)
D
Lactate production (fold)
Epi
Vitamin C
E
Stress
shLDHA
Vitamin C
Tumor volume (mm³)
Ctrl+shNC
Stress+shNC
Vitamin C+shNC
Vitamin C+Stress+shNC
Ctrl+shLDHA
Stress+shLDHA
Vitamin C+shLDHA
VitaminC+Stress +shLDHA
Days after tumor implantation
NS

Full unedited gel for Figure 1B

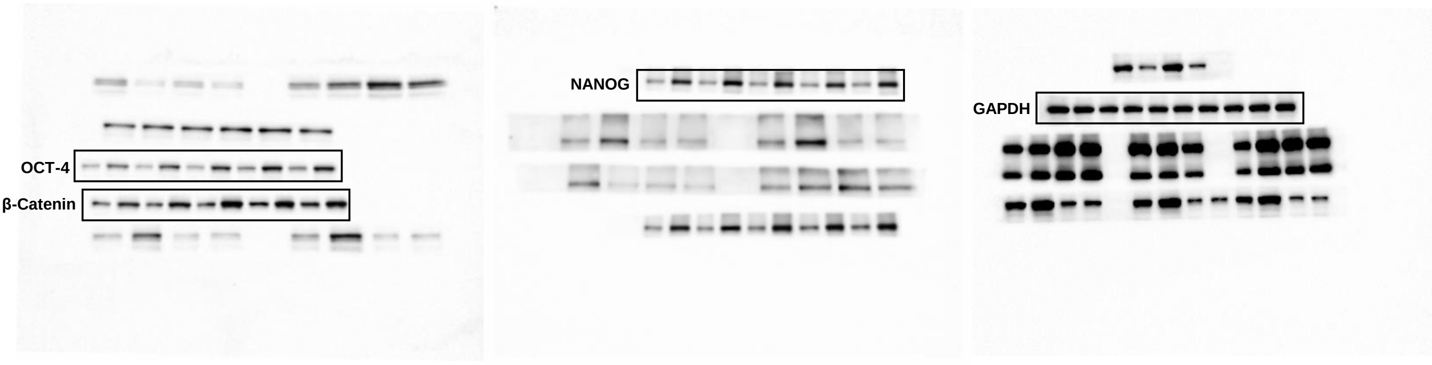

Full unedited gel for Figure 1F

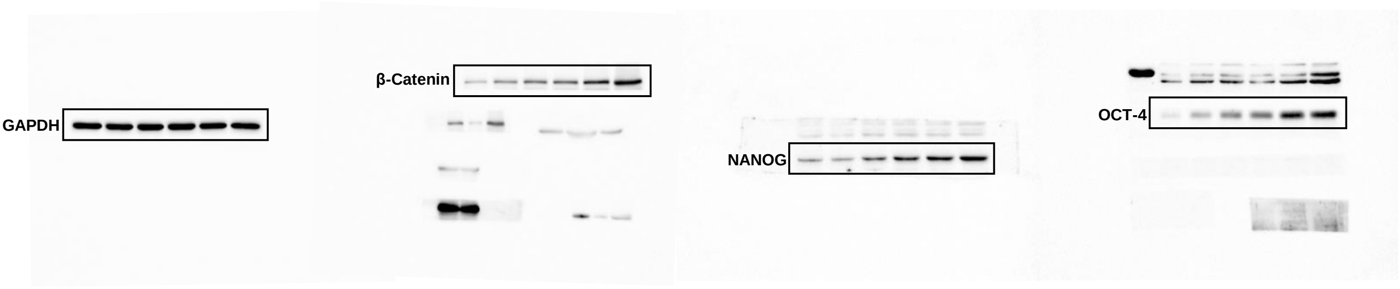

Full unedited gel for Figure 1H

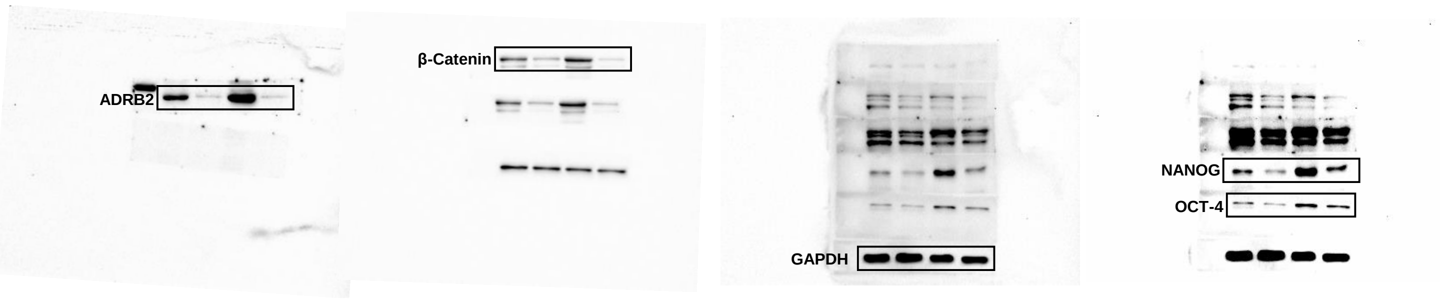

Full unedited gel for Figure 3A

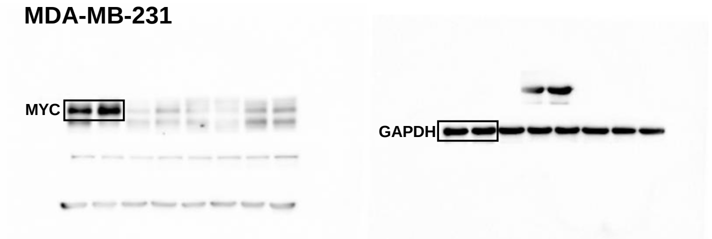

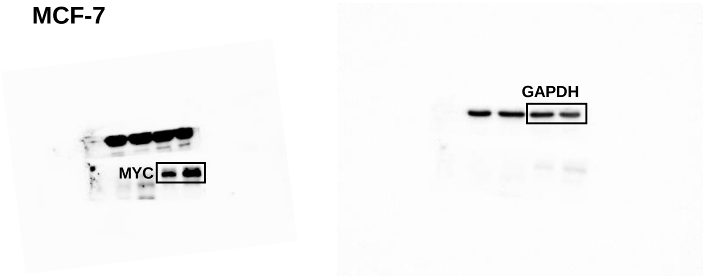

Full unedited gel for Figure 3C

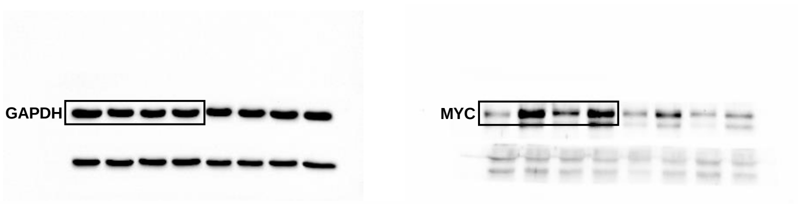

Full unedited gel for Figure 3E

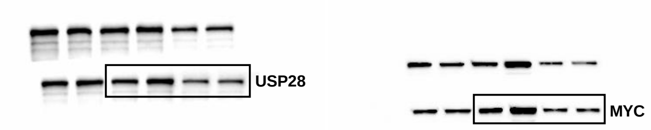

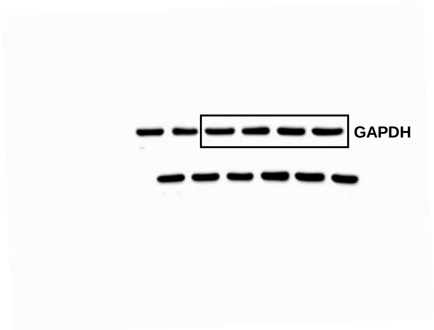

Full unedited gel for Figure 3F

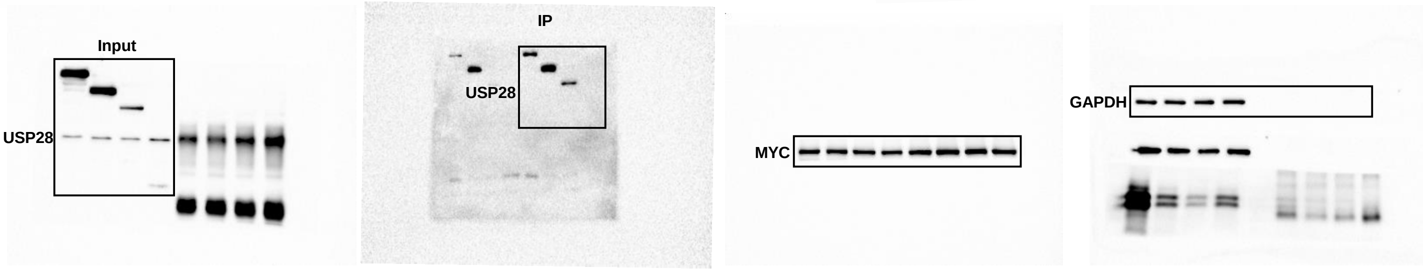

Full unedited gel for Figure 3H

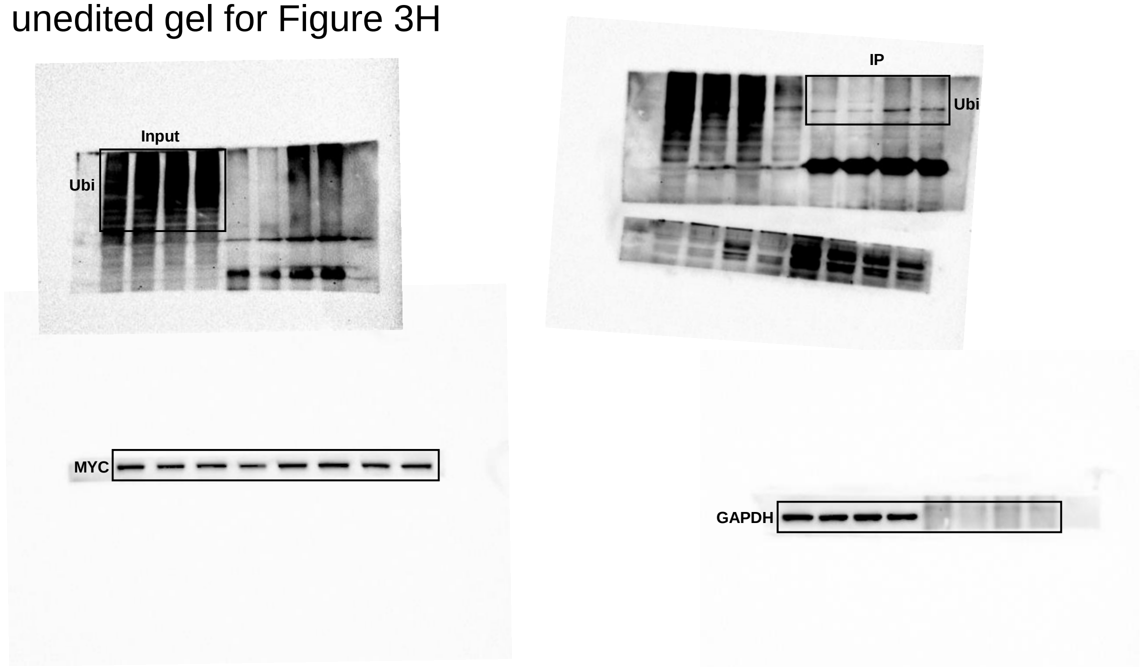

Full unedited gel for Figure 4D

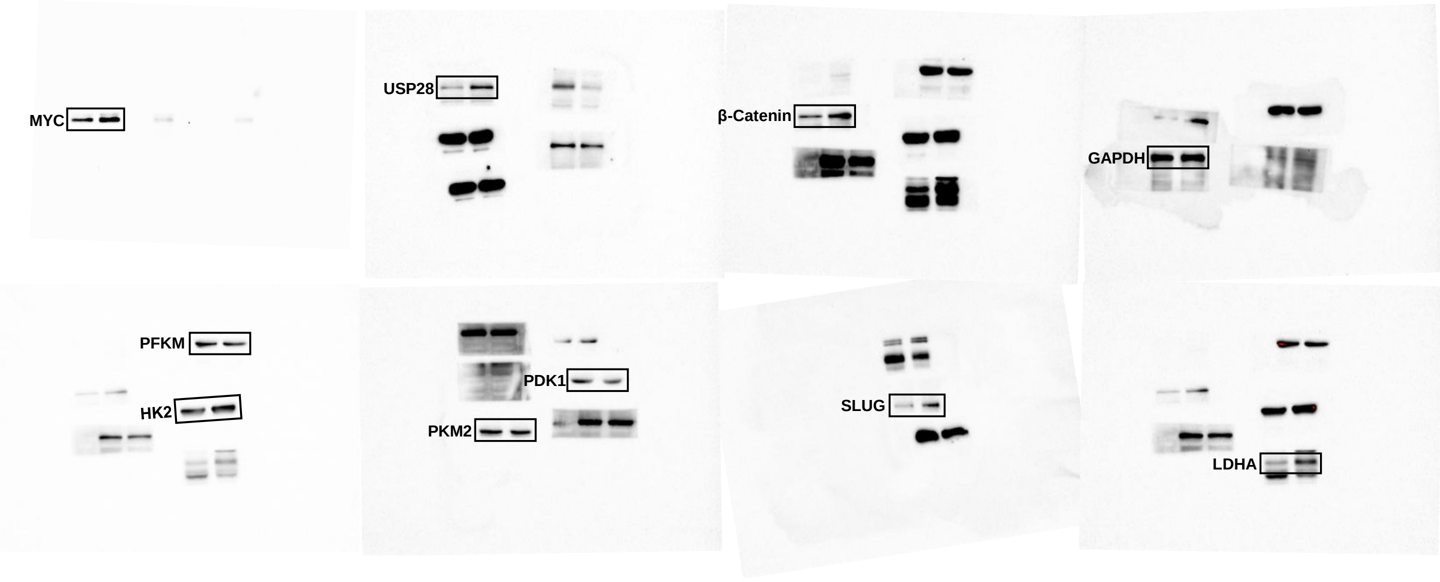

Full unedited gel for Figure 4E

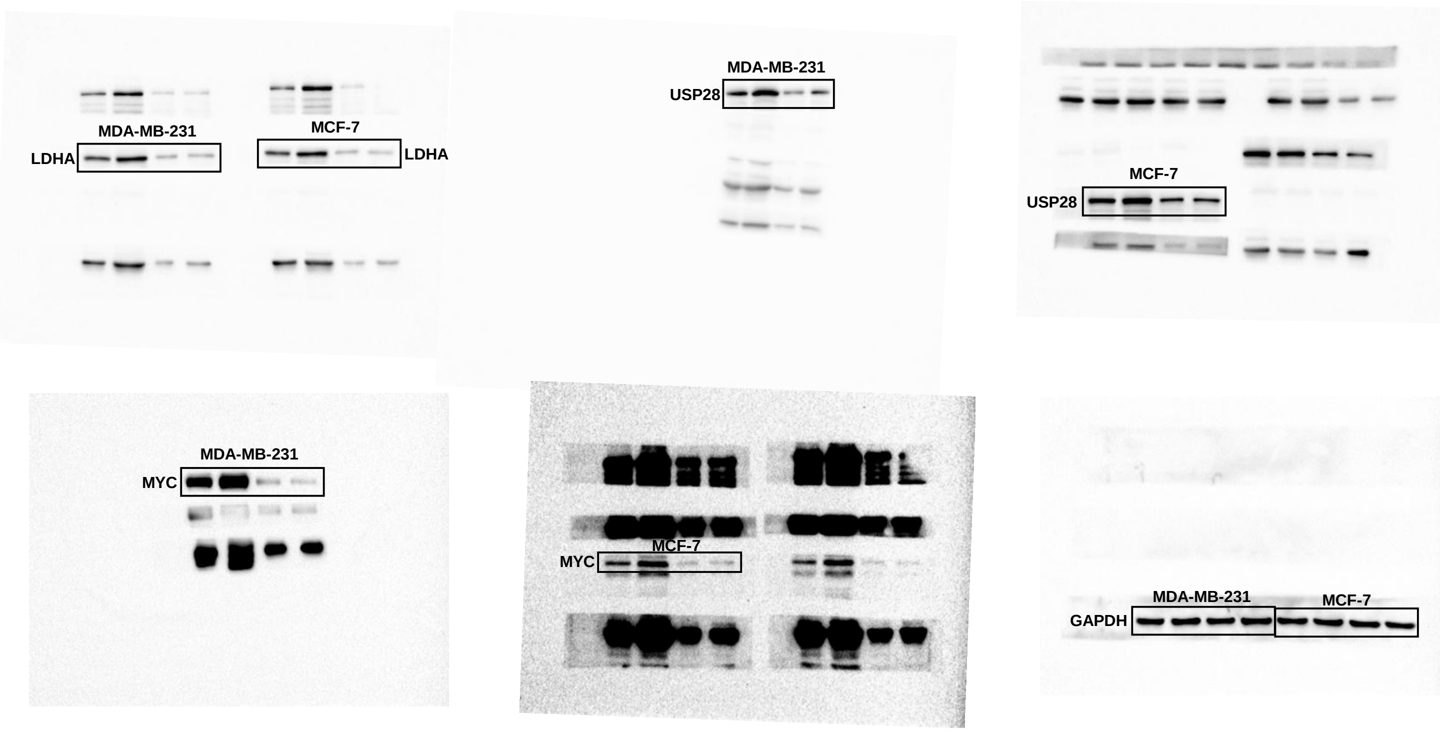

Full unedited gel for Figure 5A

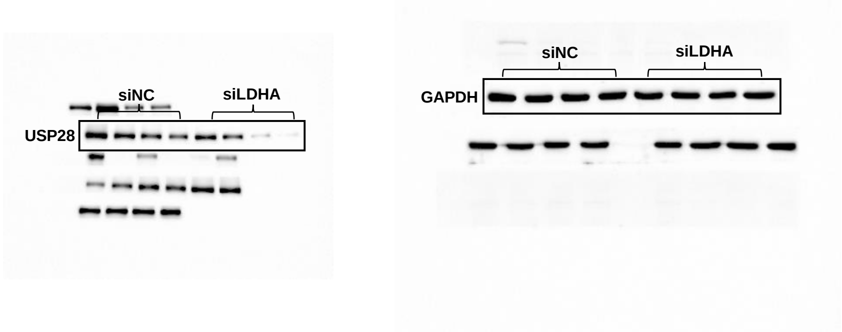

Full unedited gel for Figure 5B

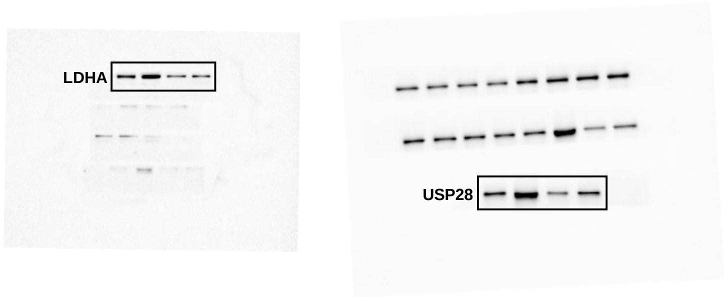

Full unedited gel for Figure 5C

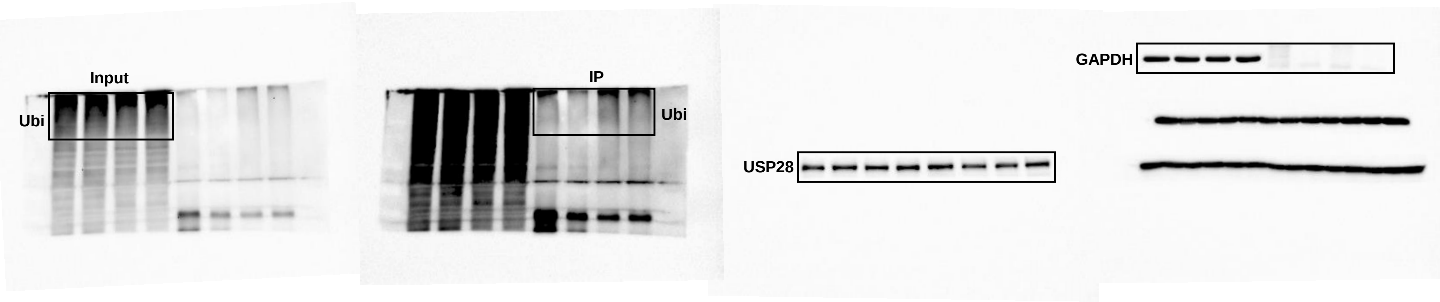

Full unedited gel for Figure 5D

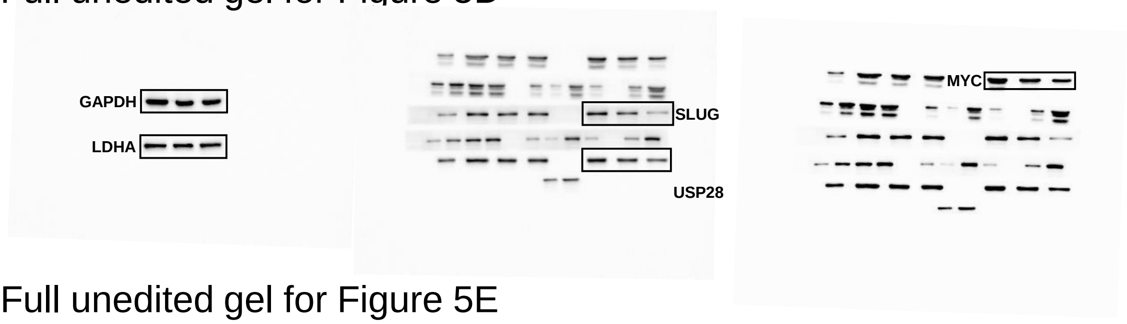

Full unedited gel for Figure 5E

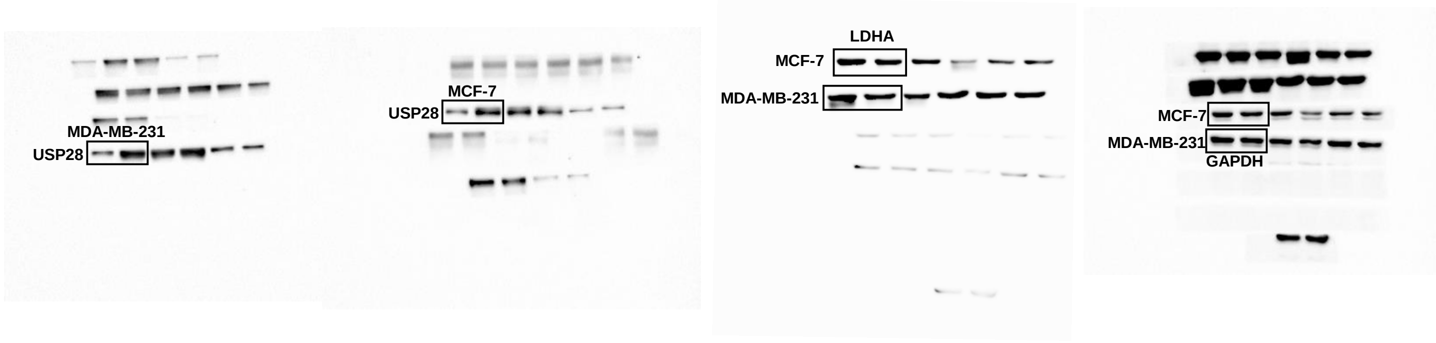

Full unedited gel for Figure 5F

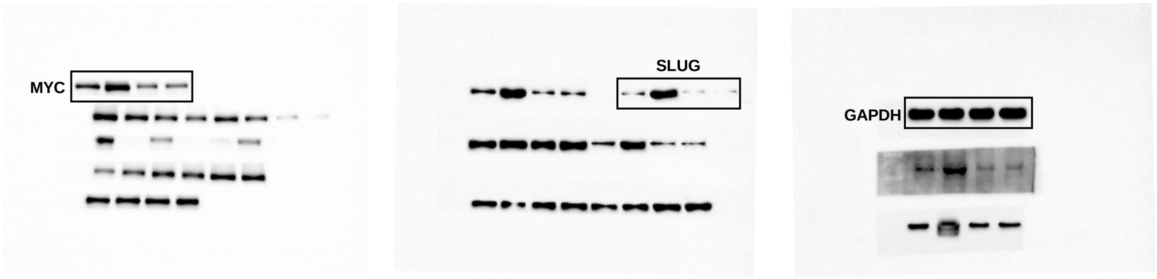

Full unedited gel for Figure 5G

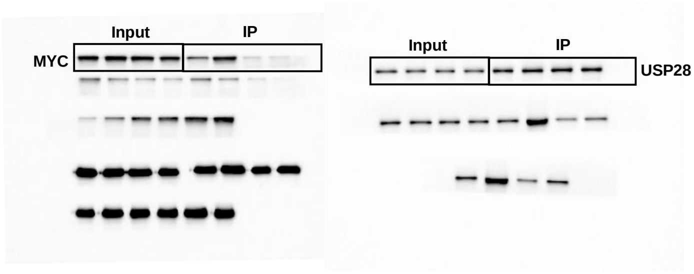

Full unedited gel for Figure 5H

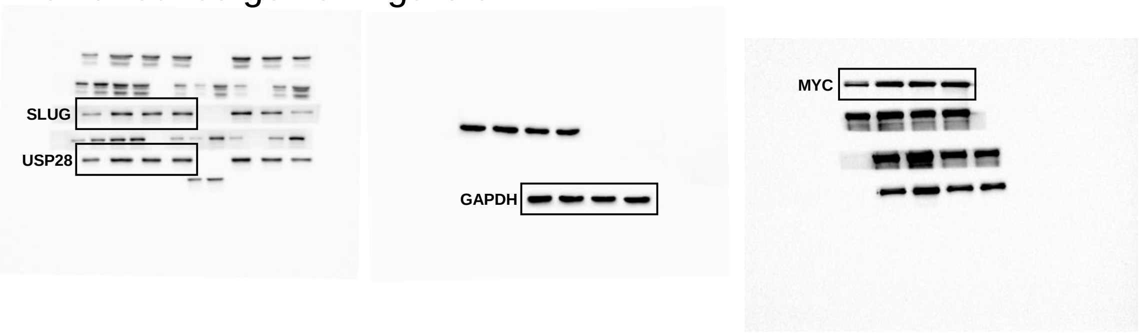

Full unedited gel for Figure 6B

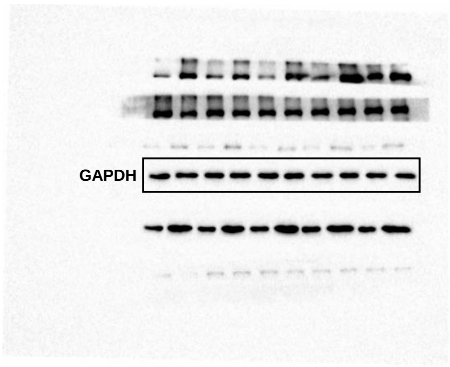

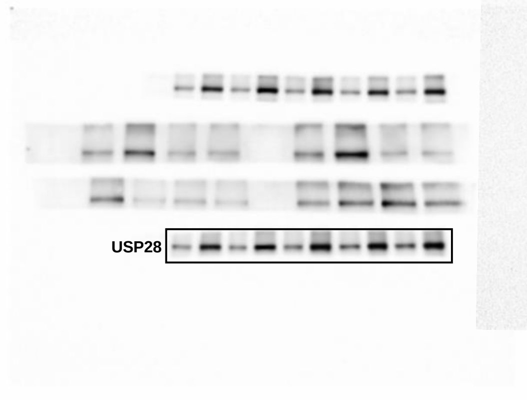

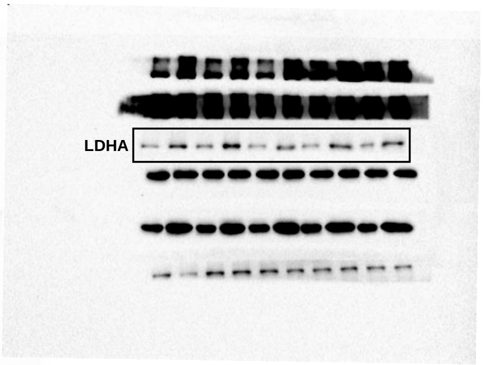

Full unedited gel for Figure 7C

MDA-MB-231

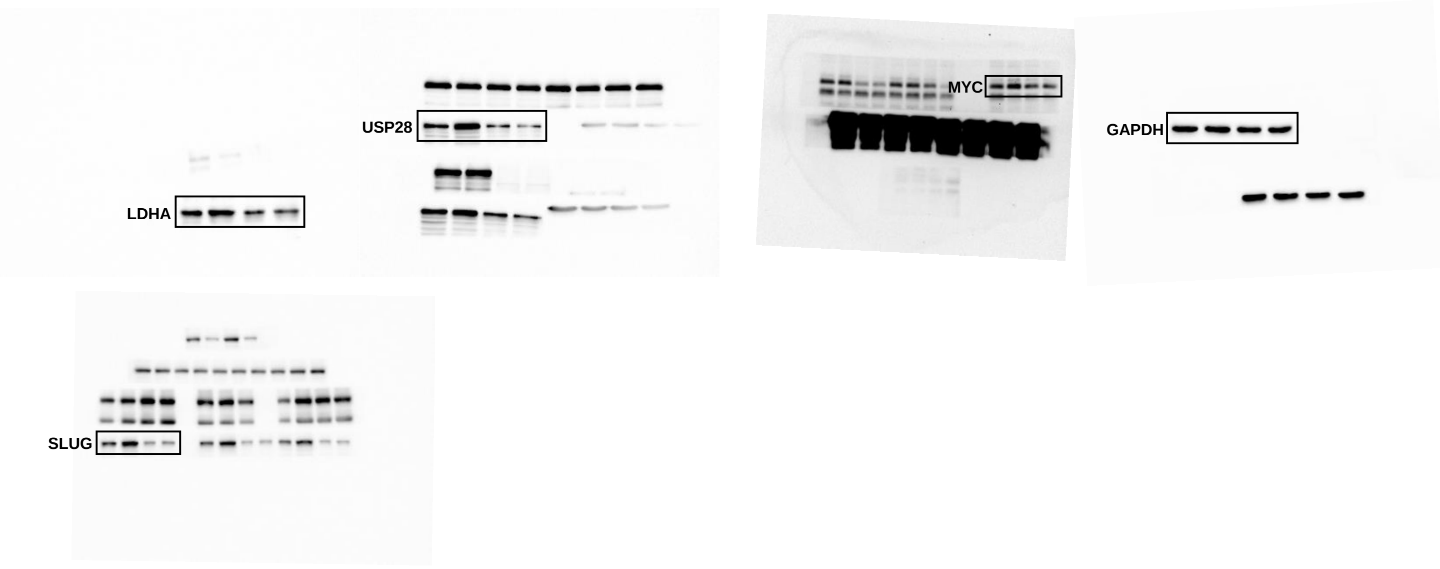

MCF-7

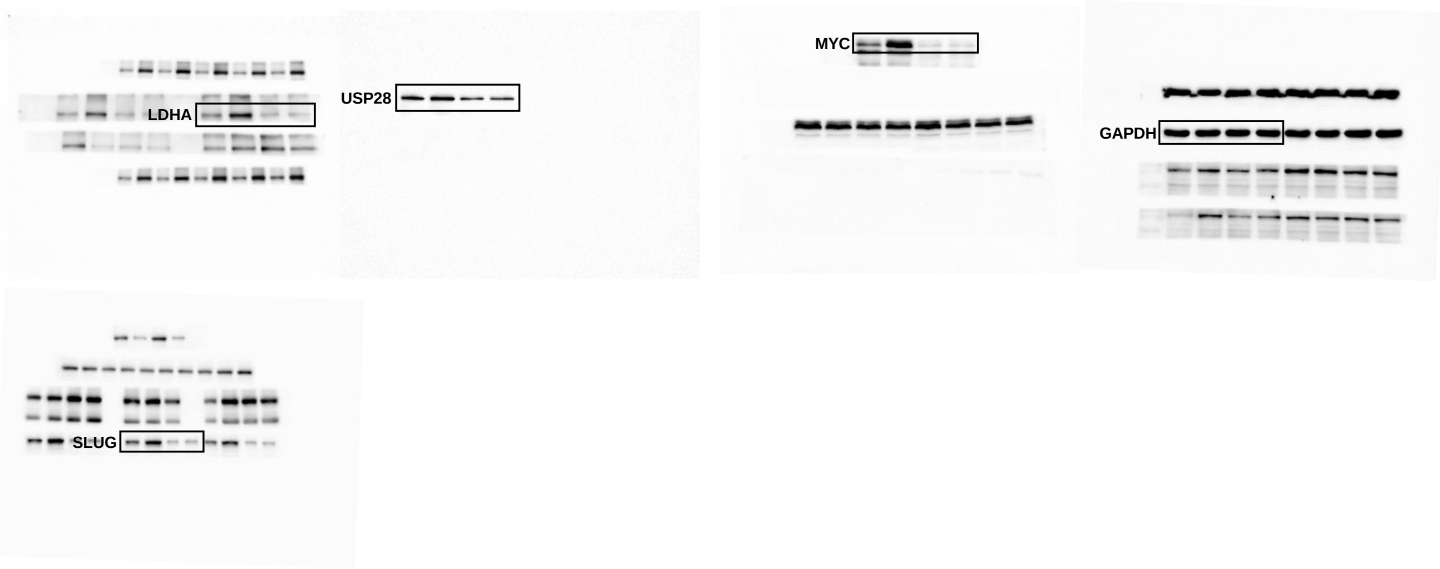

Full unedited gel for Supplemental Figure 2C

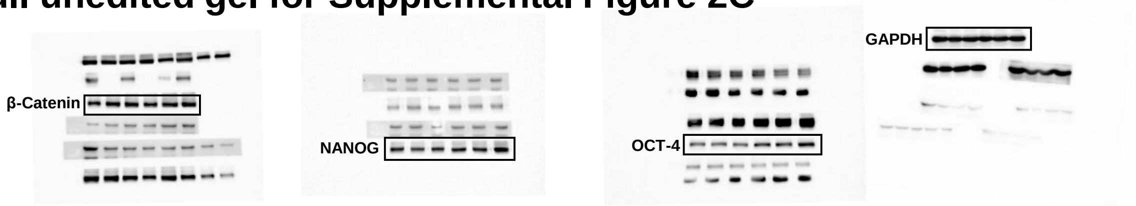

Full unedited gel for Supplemental Figure 2D

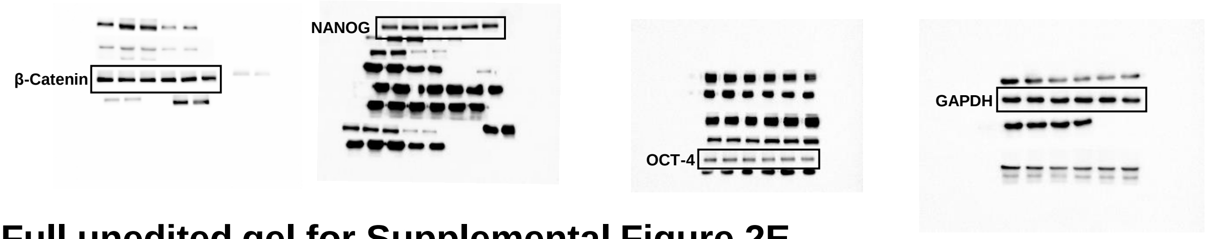

Full unedited gel for Supplemental Figure 2E

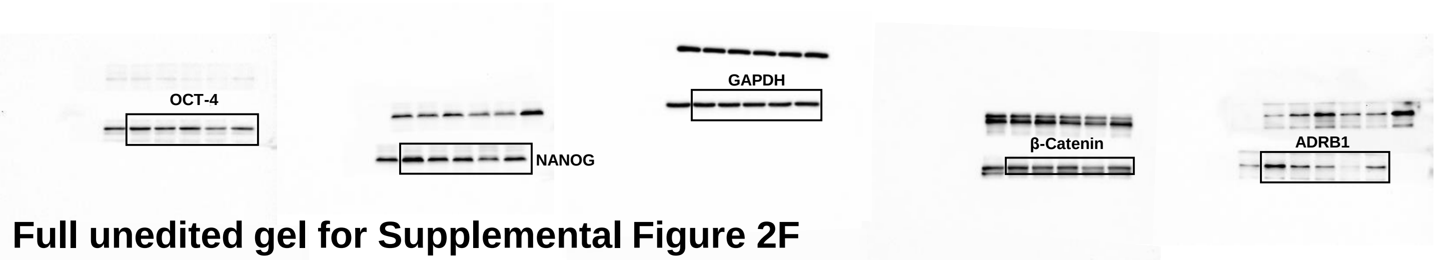

Full unedited gel for Supplemental Figure 2F

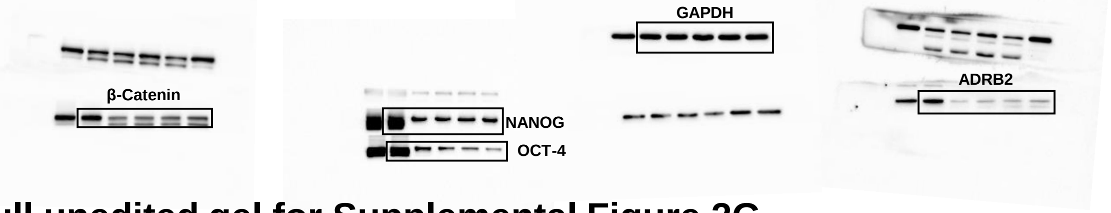

Full unedited gel for Supplemental Figure 2G

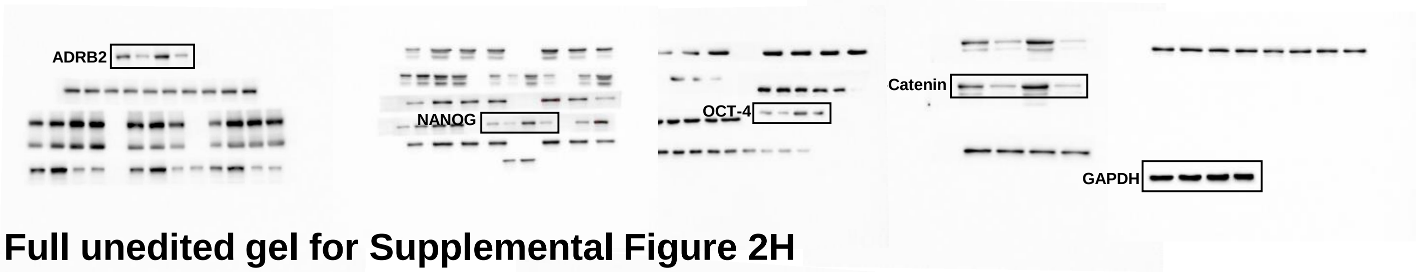

Full unedited gel for Supplemental Figure 2H

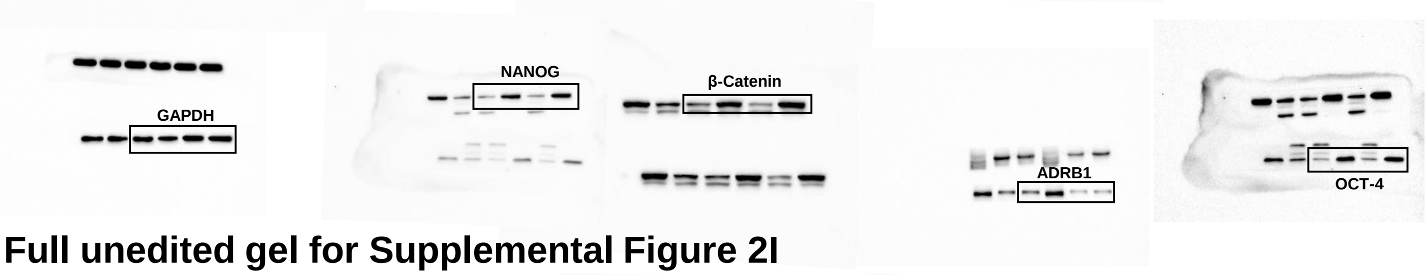

Full unedited gel for Supplemental Figure 2I

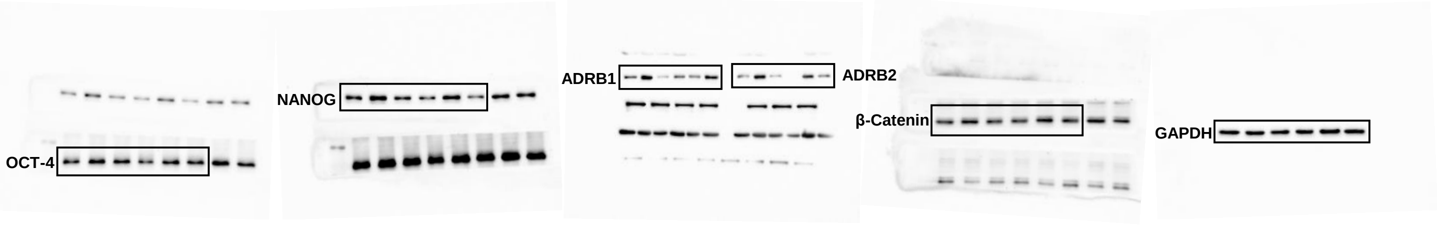

Full unedited gel for Supplemental Figure 3B

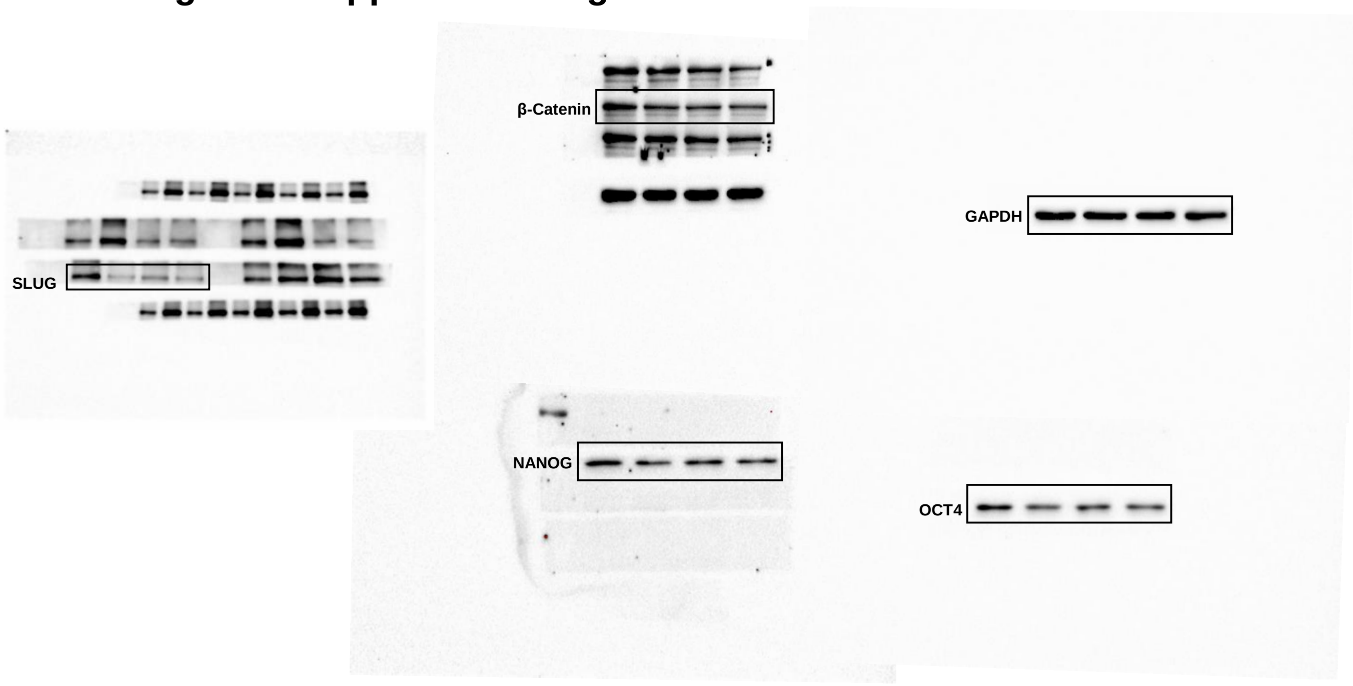

Full unedited gel for Supplemental Figure 3D

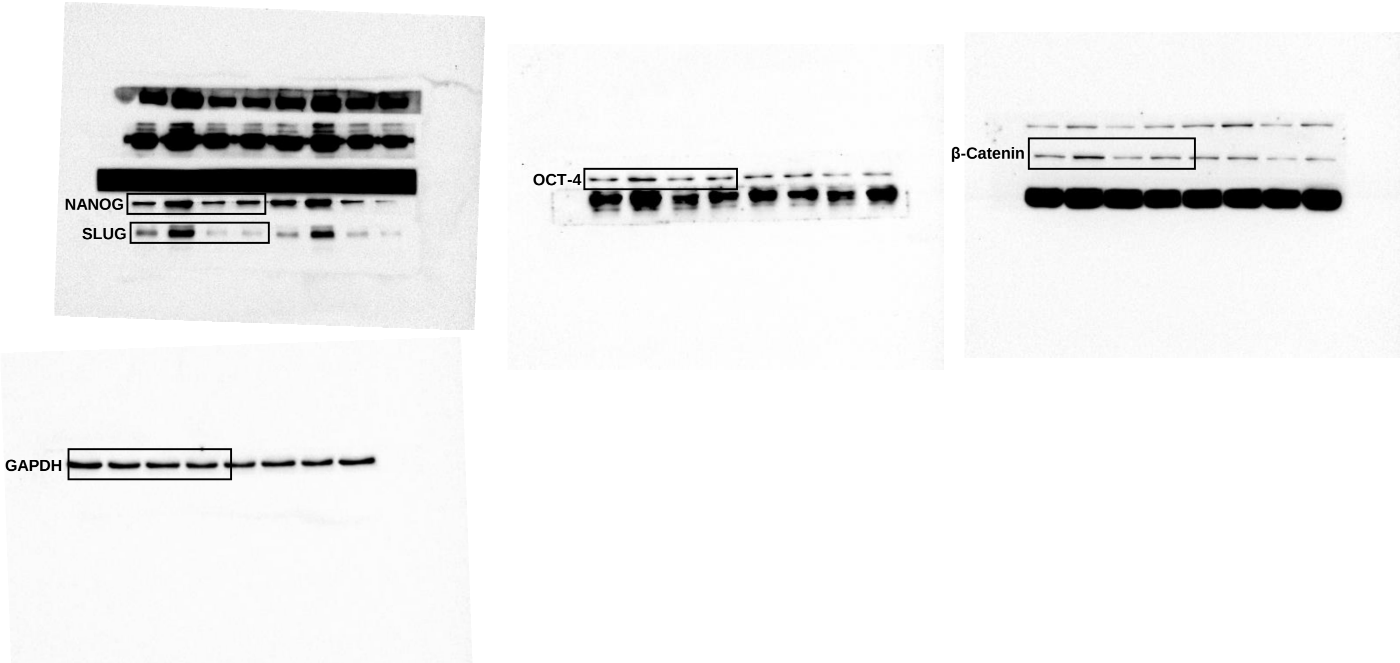

Full unedited gel for Supplemental Figure 3H

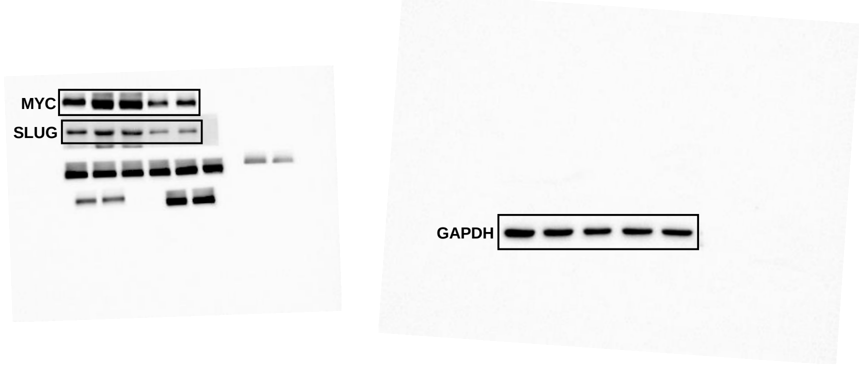

Full unedited gel for Supplemental Figure 4B

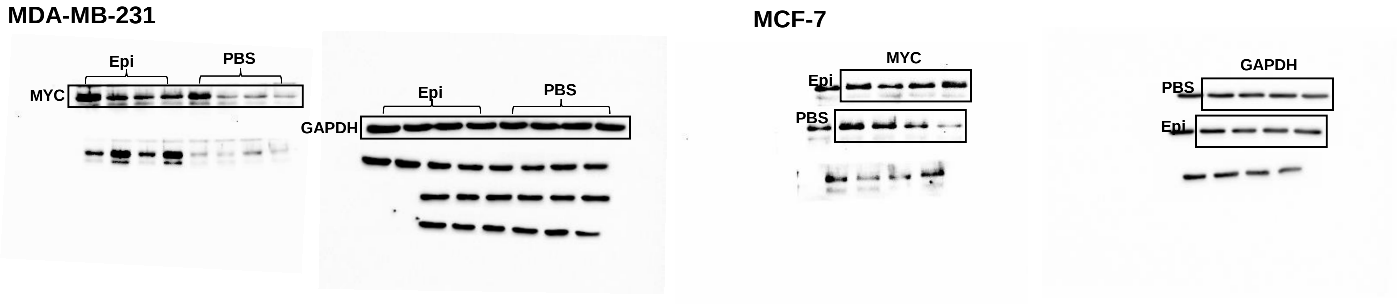

Full unedited gel for Supplemental Figure 4C

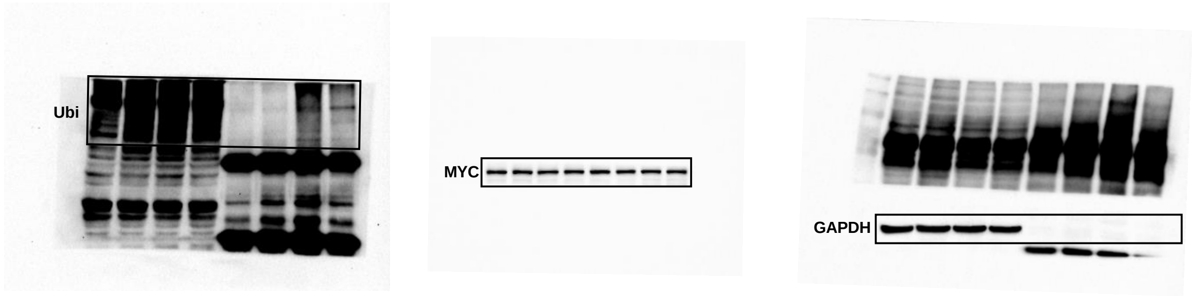

Full unedited gel for Supplemental Figure 4D

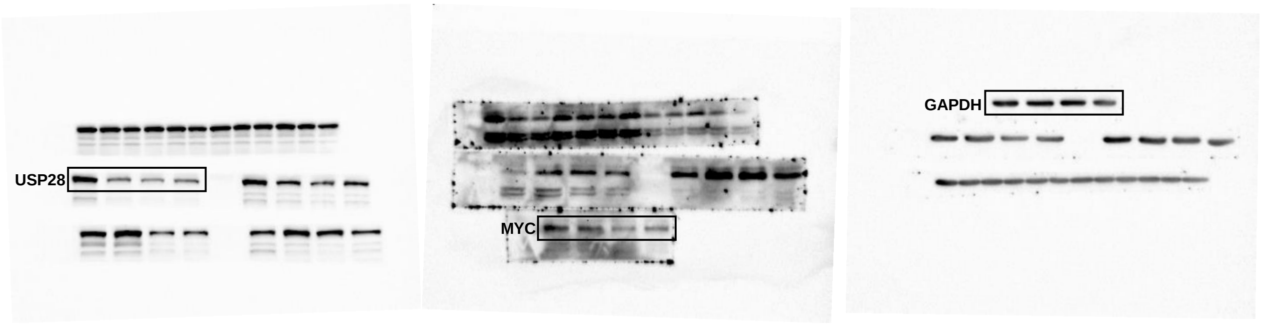

Full unedited gel for Supplemental Figure 4E

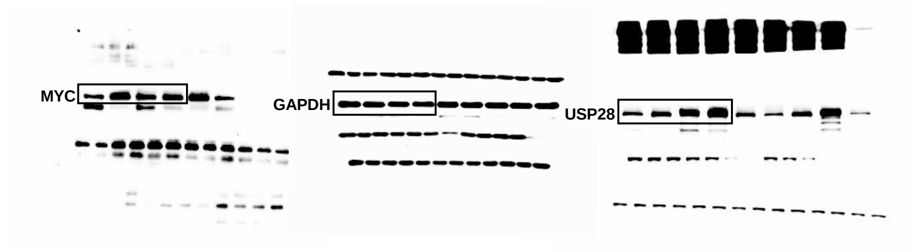

Full unedited gel for Supplemental Figure 4F

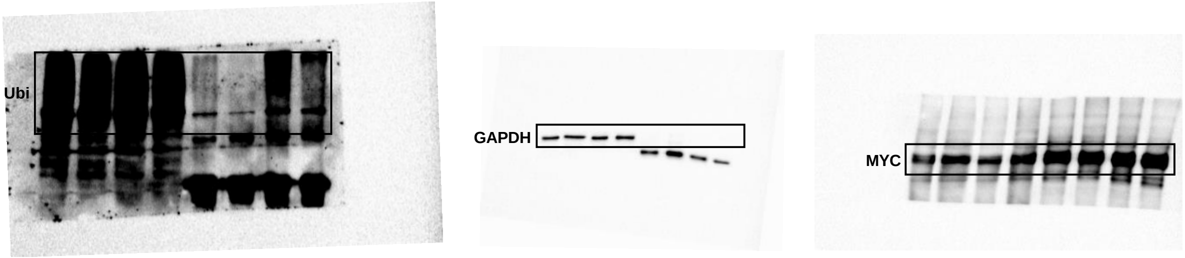

Full unedited gel for Supplemental Figure 4G

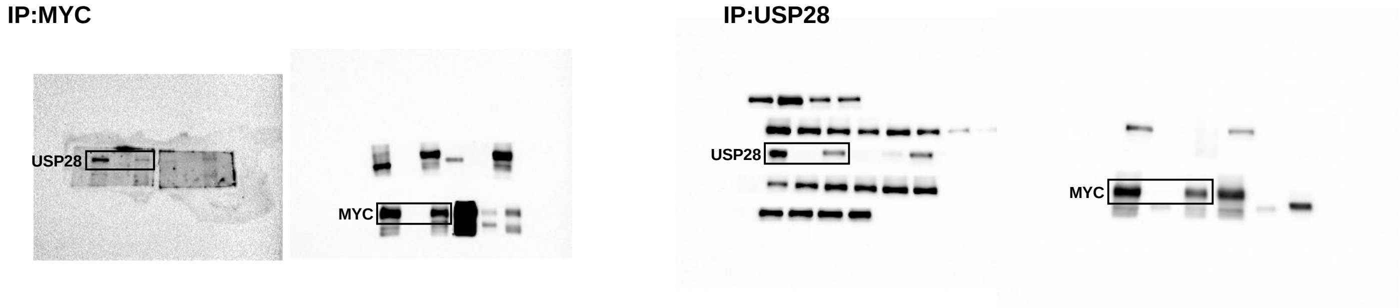

Full unedited gel for Supplemental Figure 4J

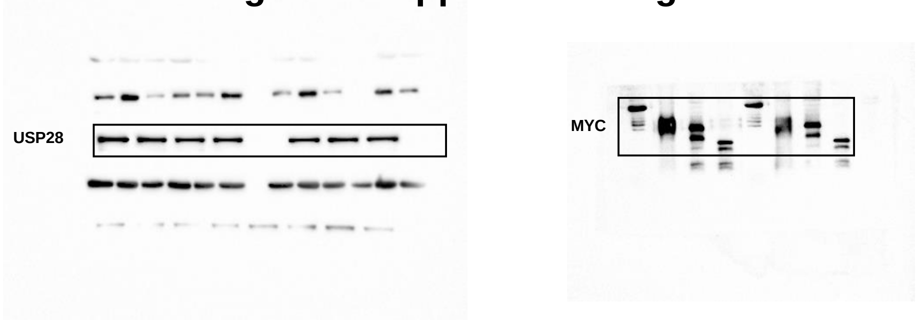

Full unedited gel for Supplemental Figure 4K

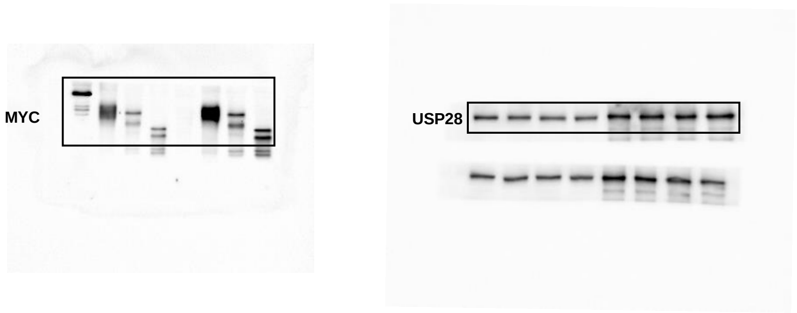

Full unedited gel for Supplemental Figure 5C

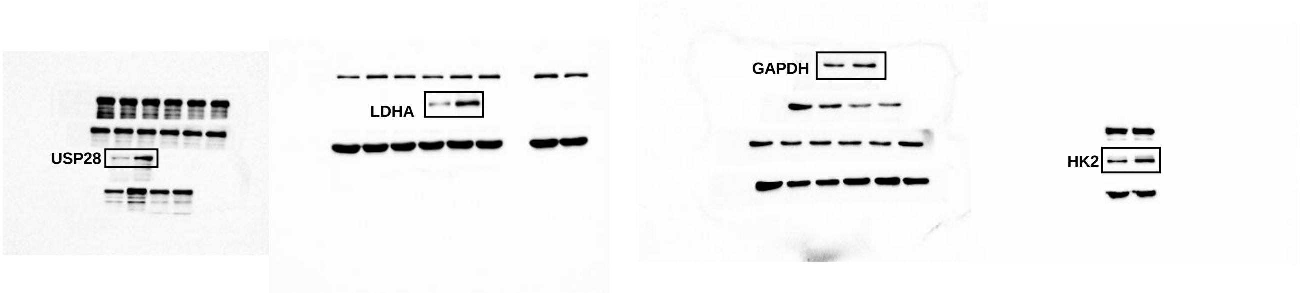

Full unedited gel for Supplemental Figure 5D

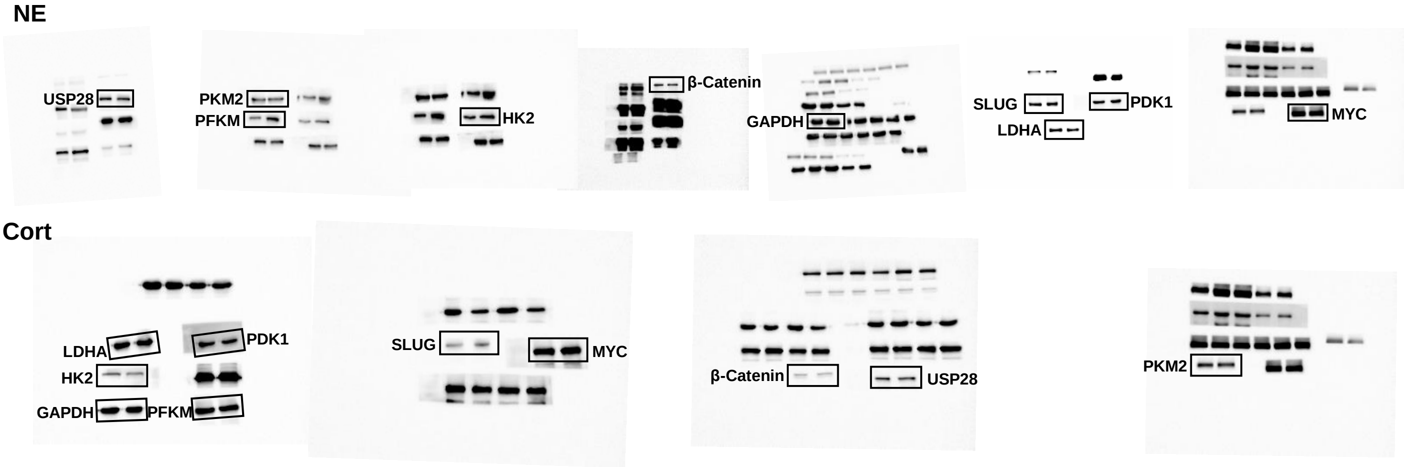

Full unedited gel for Supplemental Figure 5E

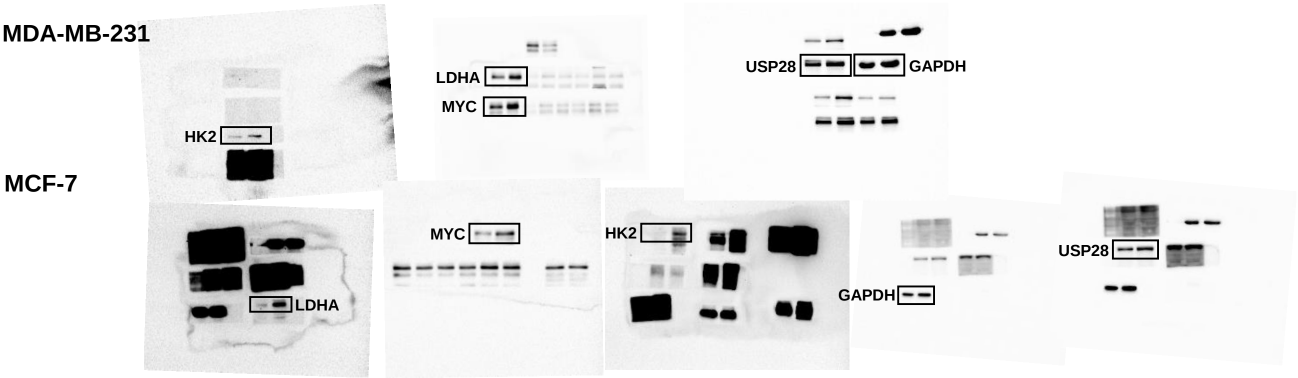

Full unedited gel for Supplemental Figure 5F

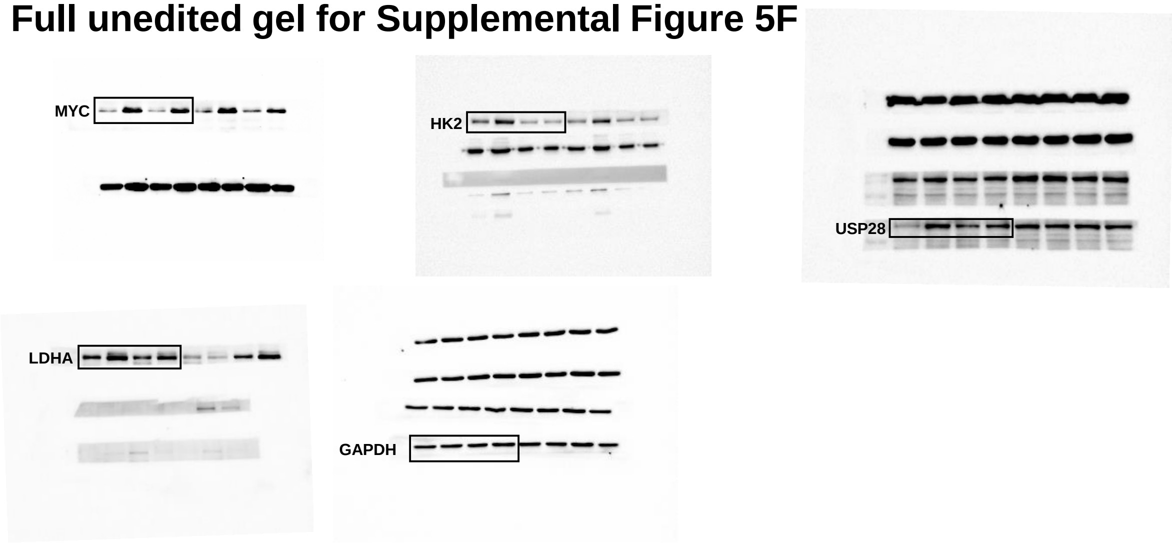

Full unedited gel for Supplemental Figure 6B

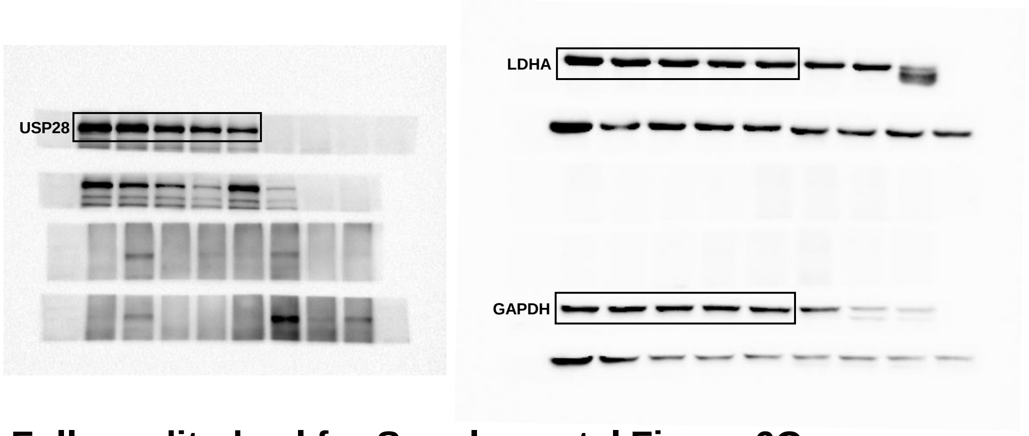

Full unedited gel for Supplemental Figure 6C

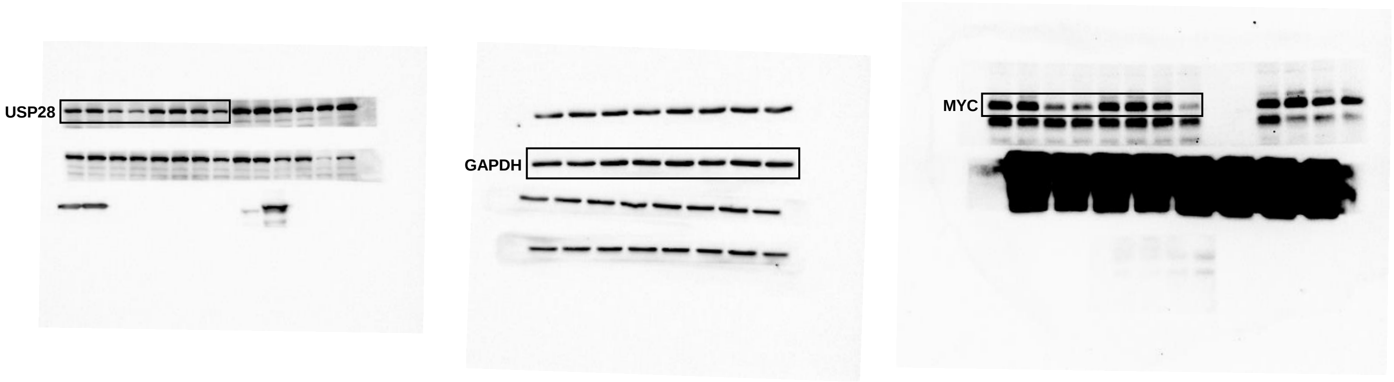

Full unedited gel for Supplemental Figure 6D

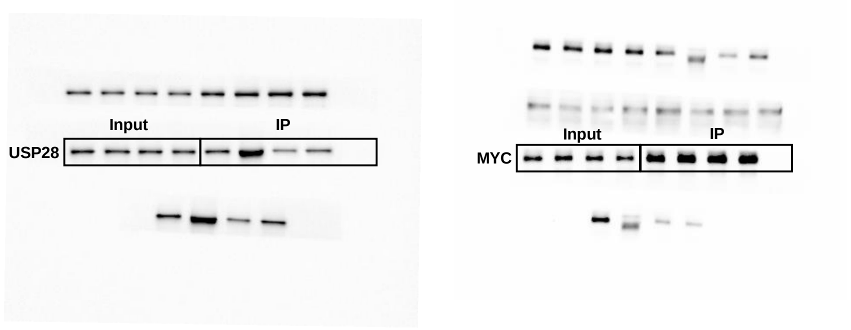

Supplement: Supplemental data [file jci-129-121685-s068.pdf]
